# Graph-based models of the *Oenothera* mitochondrial genome capture the enormous complexity of higher plant mitochondrial DNA organization

**DOI:** 10.1093/nargab/lqac027

**Published:** 2022-03-31

**Authors:** Axel Fischer, Jana Dotzek, Dirk Walther, Stephan Greiner

**Affiliations:** Max Planck Institute of Molecular Plant Physiology, Am Mühlenberg 1, 14476 Potsdam-Golm, Germany; Max Planck Institute of Molecular Plant Physiology, Am Mühlenberg 1, 14476 Potsdam-Golm, Germany; Max Planck Institute of Molecular Plant Physiology, Am Mühlenberg 1, 14476 Potsdam-Golm, Germany; Max Planck Institute of Molecular Plant Physiology, Am Mühlenberg 1, 14476 Potsdam-Golm, Germany

## Abstract

Plant mitochondrial genomes display an enormous structural complexity, as recombining repeat-pairs lead to the generation of various sub-genomic molecules, rendering these genomes extremely challenging to assemble. We present a novel bioinformatic data-processing pipeline called SAGBAC (Semi-Automated Graph-Based Assembly Curator) that identifies recombinogenic repeat-pairs and reconstructs plant mitochondrial genomes. SAGBAC processes assembly outputs and applies our novel ISEIS (Iterative Sequence Ends Identity Search) algorithm to obtain a graph-based visualization. We applied this approach to three mitochondrial genomes of evening primrose (*Oenothera*), a plant genus used for cytoplasmic genetics studies. All identified repeat pairs were found to be flanked by two alternative and unique sequence-contigs defining so-called ‘double forks’, resulting in four possible contig-repeat‐contig combinations for each repeat pair. Based on the inferred structural models, the stoichiometry of the different contig-repeat-contig combinations was analyzed using Illumina mate-pair and PacBio RSII data. This uncovered a remarkable structural diversity of the three closely related mitochondrial genomes, as well as substantial phylogenetic variation of the underlying repeats. Our model allows predicting all recombination events and, thus, all possible sub-genomes. In future work, the proposed methodology may prove useful for the investigation of the sub-genome organization and dynamics in different tissues and at various developmental stages.

## INTRODUCTION

Plant mitochondrial genomes (PMGs) vary enormously in complexity, size and structure (1,2). Early experimental evidence based on pulse-field gel electrophoresis suggested a prevailing presence of linear mitochondrial genomes in plants, with circular forms representing a minority, albeit found present (3–7). To reconcile the identification of linear or multiple sub-genome circular genomes (8–11) with the still prevailing perception that PMGs exist as master circles, just like the much smaller animal mitochondrial genomes, the creation of alternative molecular variants was suggested to be realized by pairs of repetitive elements (known as recombinogenic repeat pairs or RRPs), which can lead to two different recombination events, depending on the relative orientation of both mates of an RRP to each other (12). Such events result in different genome configurations and can generate a population of sub-circular and linear variants within the mitochondrion. Nevertheless, PMGs are typically still represented as a single circular genome, thereby not reflecting the complexity of a population of master- and sub-circles within the mitochondria of plant cells.

### Sequence-structure conversion—from sequence to graph

**Figure 1.**
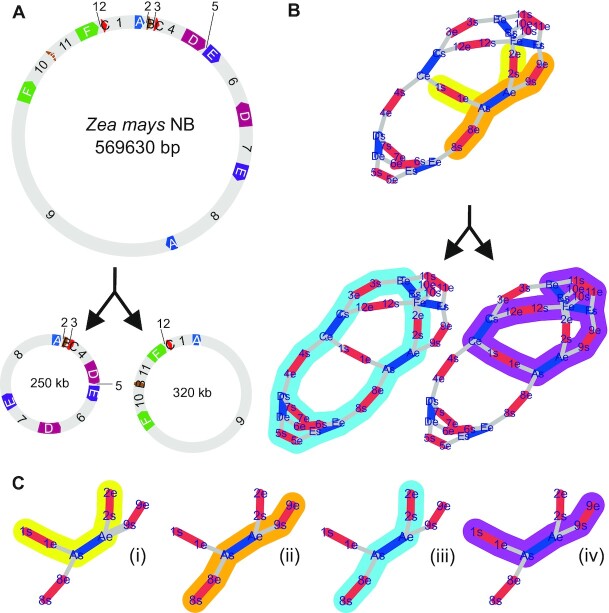
Graph-based visualization of circular maps. (**A**) Circular visualization of *Zea mays* NB, (NC_007982.1) redrawn using AngularPlasmid. Contigs between repeats are indexed numerically starting at 12 o’clock going clockwise; same holds for the RRPs for their first occurrence but using letters. The same letter was used for the second mate of an RRP when going through the master circle sequence. Both mates of RRP_A are flanked by unique sequences as follows: 1-a-2 and 8-a'-9. A recombination event at RRP_A within the master circle leads to the formation of two sub-circles in which RRP_A is now flanked by 1-a-9 and 8-a'-2. (**B**) Same information content as (A) but visualized as graphs with the following translations: Contig ends are respectively represented by two vertices linked by a red edge. Repeat ends are also represented by two vertices linked by a blue edge. Each transition between two different contigs (independent of its type, contig or repeat) is represented by a gray edge between their respective vertices. Vertices of repeats must be connected to more than one other non-repeat vertex. Color-marked paths highlight used contigs and RRPs to their corresponding circular equivalents in (A). (**C**) Shown is only RRP_A with its unique flanking sequences on both sides ( = ‘double fork’). (i–iv) all four possible contig-repeat-contig (CRC) combinations derived from the graphs in (B) for RRP_A. Again, colors highlight their origin from each of their corresponding graphs in (B).

Capturing the complexity of a variable sub-circularizing genome using current sequencing technologies proves challenging. Piecing short sequence reads together and considering alternative topologies requires specialized approaches, as typically, assembly programs tend toward assembling reads into the largest possible contig. Furthermore, the combinatorial complexity of possible assembly paths poses computational challenges. These problems can be addressed by graph-based approaches (13–15), illustrated in Figure 1. In Figure 1A, a sub-circularization event is illustrated that creates two sub-circles derived from the master circle of the *Zea mays* mitochondrial DNA (NC_007982.1) using circular representations. These circular representations can be converted into a graph-based representation as shown in Figure 1B. Comparing the circular and graph-based representation, the interpretability of the graph is much easier than that of a circular representation, as all sub-circles can be readily generated by their corresponding path through the graph (Figure 1B). Importantly, graph-based representations avoid creating the very likely misleading impression of the prevailing existence of mitochondrial genomes as single circular genomes. Also, the graph illustrates that a repeat is flanked on both sides by a set of two unique sequences, henceforth named ‘double fork’, resulting in four different contig-repeat-contig (CRC) combinations (Figure 1C).

### Study system

Historically, the evening primroses genus (*Oenothera*) represents one of the first plant models analyzed for its mitochondrial DNA (16,17). The reason for choosing this model genus already during the early days of modern molecular biology lies in its major importance for the study of extranuclear inheritance (18,19). In a now classical cross between *Oenothera berteriana* and *Oenothera odorata*, evening primrose geneticists could show as early as in the 1930s that a genetic determinant in the cytoplasm influences floral traits (20). The determinant was later called the mitochondrion, and in fact, the *Oenothera* system is now well known for the possibility to separate the genetic effects of chloroplast and mitochondria from each other (18,21). This is in contrast to commonly investigated model or crop species. Those display maternal co-inheritance of their cellular organelles (19), and in those systems, it is difficult, if not impossible, to genetically separate cytoplasmic effects of the chloroplast from that of the mitochondria (22). In *Oenothera*, however, biparental inheritance of chloroplasts, but a uniparental inheritance of mitochondria has been observed (19,23,24). Cytoplasmic effects in reciprocal crosses can therefore be attributed unequivocally to one of the two organellar genomes. This is one of the reasons, why *Oenothera* has developed into a model system for organelle genetics and population biology, in which, for example, aspects of hybrid incompatibility, organelle-mediated adaptation, speciation or organelle inheritance are being studied (e.g. (25–27)). *Oenothera* is one of the few examples, for which plastid-borne cytoplasmic male sterility (CMS) could be demonstrated (28), and is currently developing as a model to study organelle signaling involved in plant development. For these reasons, and also because putative extrakaryotic inheritance patterns of unknown origin have been described in *Oenothera* species (29–32), a high-quality mitochondrial genome sequence—that also includes structural information—is highly desirable.

The aim of this study was the assembly and annotation of the mitochondrial genomes of three major experimentally investigated species of the genus *Oenothera*, representing the species *O. villaricae* (referred to as *O. berteriana* in the genetic literature, see above), *O. biennis* and *O. elata*. The latter two are closely related and belong to the North American subsection *(Eu)Oenothera*, whereas *O. villaricae* is a member of South American subsection *Munzia*, the sister subsection of subsection *(Eu)Oenothera* (33). Assembling PMGs can lead to a set of discontinuous and unconnected contigs, especially when recombinogenic repeat pairs, RRPs, are present. Typically, insert sizes of Illumina paired-end reads are shorter than the repeat size and therefore cannot span the repeats entirely. Since, usually, it is desired to generate, ‘the one and only’ mitochondrial genome (configuration), this is considered a disadvantage. However, this perceived ‘disadvantage’ of discontinuous contigs as the outcome of a *de novo* assembler can, in fact, be turned into an advantage. As we will demonstrate here, it allows to highlight and investigate the true complexity of plant mitochondrial genomes. Instead of trying to deduce a circular configuration from a single contig, we are performing a assembly-to-graph-to-sequence conversion (i.e. deducing a much more complex sequence organization from a graph, Figure 1). For this, we developed and have employed our newly developed Semi-Automated Graph-Based Assembly Curator (SAGBAC) bioinformatics data-processing pipeline. At the core of this pipeline, the novel Iterative Sequence Ends Identity Search (ISEIS) algorithm identifies contigs with identical sequences at their ends from a short-read *de novo* assembly. We assign them by blasting all contigs from the *de novo* assembly against each other. Then, an adjacency list is created holding information on which contig ends overlap. This adjacency list is then used to construct an undirected graph, which can be visualized. The obtained genome graph model is then employed to identify all possible genome configurations (circular or linear), produced by the recombinogenic repeat pairs (Figure 1). This new assembly and visualization approach offers a solution to the assembly of the highly complex higher plant mitochondrial DNA. Its graph-based visualization allows for a better interpretability than the classical mini and master circle model. The proposed methodology may prove useful for the investigation of the sub-genome organization and dynamics of PMGs.

## MATERIALS AND METHODS

### Plant material

Plant material used here was derived from the *Oenothera* germplasm collection harbored at the Max Planck Institute of Molecular Plant Physiology, Potsdam-Golm, Germany (34). *Oenothera biennis* strain suaveolens Grado (named hereafter *O. biennis*) (35) and *O. elata* ssp. *hookeri* strain johansen Standard (named hereafter *O. elata*) (36) belong to subsection *Oenothera*. *Oenothera villaricae* strain berteriana Schwemmle (*syn*: *O. berteriana* Erlangen, named hereafter *O. villaricae*) (20) is part of subsection *Munzia*. As abbreviations for the strains/species, the following code was used: berS = *O. villaricae*, suavG = *O. biennis*, johSt = *O. elata*). The line reassembles the original material used by Julius Schwemmle and Axel Brennicke. For details on their taxonomy, see (33,37,38).

### Plant cultivation

Seeds were germinated in Petri dishes on wet filter paper supplemented with 0.05% (v/v) of Plant Preservative Mixture (Plant Cell Technology Store, Washington, DC, USA) at 27°C and 100–150 μE m^–2^ s^–1^ To obtain etiolated seedlings, Petri dishes were wrapped with aluminum foil immediately after germination when root tips became visible. After 3 days, material was harvested and frozen in liquid nitrogen. If older material was needed, plants were grown to the appropriate developmental stage in a glasshouse at 22°C and 300–400 μE m^–2^ s^–1^ in a 16 h photoperiod.

### Isolation of mitochondria

Mitochondria were isolated from mature rosette leaves following a modified protocol from (39,40): First, our homogenization buffer was supplemented with 25 mM boric acid and 10 mM EGTA. Both compounds effectively liquefy viscous homogenates from *Oenothera* leaf tissue (Peter Westhoff, personal communication). While boric acid reacts with 1,2-dihydroxy groups of polysaccharides (41), EGTA specifically chelates Ca^2+^ ions. Those are often associated with gelling properties of mucilage (42). In addition, in an essential mitochondria purification step, a triple Percoll density gradient (18%, 23%, 50%) was employed.

During the isolation procedure all steps were performed at 4°C. About 100 g of leaves tissue were incubated for approximately 30 min in ice water and dried using a salad spinner. Afterward, 1 l of BoutHomX homogenization buffer (0.4 M sucrose, 50 mM Tris, 25 mM boric acid, 10 mM EGTA, 10 mM KH_2_PO_4_, 1% [w/v] fat free BSA, 0.1% [w/v] PVP-40, pH 7.6 with KOH, and 5 mM freshly supplemented β-mercaptoethanol) was added and leaves ground 5 × 5 s in a razor blade grinder (Waring® Blender 8010E, Waring Commercial, New Hartford, NY, USA). The homogenate was filtered in 100 ml aliquots through two layers of mull (Verbandmull ZZ, Hartmann, Heidenheim, Germany) and one layer of Miracloth (Merck, Darmstadt, Germany), respectively. Then it was centrifuged in three 250 ml aliquots for 15 min at 5000 × *g*. Chloroplast containing pellets were discarded and the supernatants centrifuged again for 20 min at 22 000 × *g*. Mitochondria pellets were then resuspended in 20 ml BoutWashY (0.4 M mannitol, 10 mM KH_2_PO_4_, 0.1% [w/v] fat free BSA, pH 7.6 with KOH) each, using a 30 cm^2^ Potter homogenizer (0.1–0.15 mm mill chamber tolerance; Wheaton, Millville, USA). Afterward, solutions of resuspended mitochondria were combined, dispensed into four 50 ml centrifugation tubes and volumes adjusted to 50 ml with BoutWashY. Following a centrifugation at 3000 × *g* for 5 min the supernatant was used for further purification and centrifuged at 18 000 × *g* for 15 min. The obtained pellets were re-suspended with a brush in all together 8 ml of 0% Gradient Medium (0.3 M sucrose, 5 mM KH_2_PO_4_, 0.1% fat free BSA), tubes rinsed with 2 ml 0% Gradient Medium and the two solutions combined. Still unresolved mitochondria were homogenized in a 15 cm^2^ Potter homogenizer (0.1–0.15 mm mill chamber tolerance; Wheaton, Millville, USA). After this procedure, an additional centrifugation step at 3000 × *g* for 5 min was performed. Then, the supernatant of the sample was split into halves and carefully loaded on two three-step density gradients (5 ml of 50% Percoll, 10 ml of 23% Percoll, and 5 ml of 18% Percoll in 0.3 M sucrose, 10 mM KH_2_PO_4_, and 0.1% fat free BSA, pH 7.6 with KOH; freshly prepared in a 30 ml Corex tube). Gradients were centrifuged with decreased acceleration at 10 000 × *g* for 40 min and decelerated without use of the centrifuge brake. Intact mitochondria were extracted with a pipette from the bottom of the 23–50% interphase. For washing, the mitochondria fraction was dissolved in 50 ml BoutWashY and centrifuged four times while reducing the volume in each centrifugation step. The pellet was finally diluted in 400 μl TENTS buffer for further analyses. Purity of the isolated mitochondria fraction were directly assessed by confocal microscopy (employing MitoTracker and DAPI staining to visualize mitochondria and broken nuclei, respectively; chloroplasts were detected based on their autofluorescence) and western blot analyses of marker proteins for the individual genetic compartments (COXII, CF1α/β, and H3ab). Real-time PCR on isolated mtDNA (see below) with appropriate marker probes showed that by applying our protocol, an enrichment of mtDNA from ∼1.5% in total DNA isolations to up to ∼95% was achieved. For details, see (24).

### Mitochondrial DNA extraction

Mitochondria pellets from above were resuspended in TENTS buffer (100 mM Tris/HCl at pH 8.0, 50 mM EDTA, 0.5 M NaCl, 0.2% [v/v] Triton X-100; 1% [w/v] SDS) and incubated for 15 min at 60°C while shaking at 400 rpm. After adding 100 μl of a 10 mg/ml RNase A solution (50 U/mg; Roche Diagnostics GmbH, Manheim, Germany) samples were incubated for 1.5 h at 37°C. Subsequently, 100 μl of Proteinase K solution (10 mg/ml; Sigma-Aldrich, Steinheim, Germany) were added and samples placed over night at room temperature. Then, 630 μl phenol/chloroform/isoamyl alcohol (25:24:1) was added, probes incubated for 10 min at room temperature and then centrifuged at 18 000 × *g* for 10 min. After this, the supernatant was removed, 630 μl chloroform added and samples were centrifuged again at 18 000 × *g* for 10 min. Precipitation of mitochondrial DNA (mtDNA) was performed with 1/10 vol of 5 M NH_4_-acetate and 1 vol isopropanol overnight at −20°C. After centrifugation for 45 min at 20 000 × *g*, the pellet was washed two times with 70% v/v and 100% v/v ethanol and re-suspended in 10 μl of 5 mM Tris/HCl, pH 7.6.

### Extraction of total DNA

Total high-molecular weight DNA for PacBio sequencing was obtained from etiolated seedlings with a CTAB/phenol-based method. For Southern blotting, an IGEPAL/phenol-based DNA isolation protocol was applied to plants at the early rosette stage (34). Both procedures, as well as subsequent purification of the DNA with anion-exchange columns, were published previously in (43). For PCR reactions, we used total DNA obtained with the DNeasy Plant Mini Kit (Qiagen, Hilden, Germany) applying minor modifications to the manufacture’s protocol as reported in (44).

### Extraction of total RNA

Total RNA from the emerging fourth leaf of *O. elata* was isolated using TRIzol (Invitrogen, Thermo Fisher Scientific, Waltham, MA, USA) in a protocol adjusted to the specific needs of *Oenothera* tissue that is rich in mucilage and phenolic compounds. In contrast to the previously published RNA isolation protocols for evening primrose (43,45), the procedure described here omits silica membrane columns and allows direct precipitation of RNA from aqueous solutions. Following this protocol, depending on tissue age, 25–75 mg leaf material are frozen in liquid nitrogen and ground using a mixer mill. Then 800 μl of IDS buffer (120 mM Tris/HCl at pH 8.0, 120 mM EDTA at pH 8.0, 2.4% IGEPAL [v/v], 1.2% SDS [w/v], 1.2% PVP [w/v]) and 200 μl of β-mercaptoethanol are added and the sample vortexed until the powder has completely dissolved. Subsequently, the homogenate is incubated for 10 min at 60°C under medium shaking and cell debris removed by centrifugation at room temperature. Subsequently, the supernatant is mixed with 1.0 ml of TRIzol (Invitrogen, Thermo Fisher Scientific, Waltham, MA, USA) and incubated for 10 min at 60°C under medium shaking. Than the sample is incubated on ice for 5 min and centrifuged at 12 000 × *g* for 5 min at 4°C. The upper phase is collected, treated with chloroform:isoamyl alcohol (24:1) once, than repeatedly with acidic phenol:chloroform (5:1) at a pH of 4.5 until the interphase was clean, and then again with chloroform:isoamyl alcohol (24:1) twice. RNA is precipitated with 1 vol of isopropanol and washed in 75% of ethanol. To resolve the pellet in ddH_2_O, RNA is incubated for 10 min at 60°C under medium shaking.

### Standard polymerase chain reaction

PCR reactions were performed from total DNA using standard methods employing DreamTaq polymerase (Thermo Fisher Scientific, Waltham, MA, USA). All primers used in this work are listed in Supplementary Table S1 and were obtained from Eurofins MWG Operon (Ebersberg, Germany).

### Detection of radiolabeled DNA via Southern blot

About 3 μg of total DNA per sample was digested over night with appropriate restriction enzymes and subsequently separated on a 1% agarose gel. DNA was then transferred by a capillary transfer to a nylon membrane (Amersham Hybond-XL, GE Healthcare, UK) using 10× SSC buffer (1.5 M NaCl, 0.15 M sodium citrate, pH 7.0). After crosslinking, the membrane was prehybridized with Church buffer (1% BSA, 1 mM EDTA, 7% SDS, 0.5 N NaHPO_4_, pH 7.2) for 1 h at 65°C. Radiolabeled DNA probes derived from PCR products were used for detection of the corresponding DNA sequences. Labeling with ^32^P dCTPs was performed using the Maxiscript Kit (Ambion, Darmstadt, Germany) according to the manufacturer’s protocol. Radioactive probes were transferred into hybridization tubes containing the nylon membrane and Church buffer and incubated over night at 65°C. After three washing steps, once for 20 min in Wash Solution I (2× SSC, 0.1% SDS) and twice for 20 min in Wash Solution II (0.5× SSC, 0.1% SDS), the radioactive signal was detected with the Radioisotope Image Analyser Typhoon Trio (GE Healthcare, UK) after 1 day incubation. For detecting very low signals, the membranes were incubated for 24–168 h at −80°C on Amersham HyperfilmTM-ECL (GE Healthcare, UK).

### Sanger sequencing

Sanger sequencing of PCR products was done at Eurofins MWG Operon (Ebersberg, Germany).

### Next-generation sequencing

Next-generation sequencing technologies, libraries and nucleotide origin used in this work are summarized in Supplementary Table S2 and detailed in the following paragraphs.

### Roche 454 sequencing

454 sequencing was performed at Eurofins MWG Operon (Ebersberg, Germany). About 100 ng of isolated mtDNA were pre-amplified using the GenomiPhi HY DNA Amplification Kit (GE Healthcare, Chalfont St Giles, UK). Then, samples were nebulized, emulsionPCR performed and single-end read libraries sequenced on a Roche/GS FLX Titanium platform (Roche Diagnostics GmbH, Manheim, Germany).

### Illumina paired-end sequencing of isolated mtDNA from *O. elata*

Library preparation and sequencing was performed at the Max Planck Genome Centre Cologne, Germany. In brief, 100 ng mtDNA were initially fragmented by sonication (Covaris S2; Covaris, Woburn, MA, USA), followed by library preparation with NEBNext Ultra Directional DNA Library Prep Kit for Illumina (New England Biolabs, Ipswich, MA, USA). The latter included 9 cycles of PCR amplification. At all steps, quality and quantity were assessed via capillary electrophoresis (TapeStation; Agilent Technologies, Santa Clara, CA, USA) and fluorometry (Qubit; Thermo Fisher Scientific, Waltham, MA, USA). Libraries were immobilized and processed on to a flow cell with cBot (Illumina, San Diego, CA, USA) and subsequently sequenced on a HiSeq 3000 system (Illumina, San Diego, CA, USA) with 2 × 150 bp paired-end reads.

### Illumina paired-end sequencing of isolated mtDNA from *O. biennis* and *O. villaricae*

Creation of shotgun libraries was done by using a commercially available kit (NEBNext DNA Sample Prep Master Mix Set 1; New England Biolabs, Ipswich, MA, USA). In brief, genomic DNA was fragmented using a Covaris E210 Instrument (Covaris, Woburn, MA, USA). Then end-repair, A-tailing and ligation of indexed Illumina Adapter, agarose gel size selection and amplification was performed. The resulting fragments were cleaned up, pooled and sequenced on a HiSeq 2000 at Eurofins MWG Operon (Ebersberg, Germany) with 2 × 101 bp paired-end reads.

### Illumina mate-pair sequencing of isolated mtDNA from *O. elata*

A mate-pair library was generated from mtDNA for paired end sequencing according to the protocol of the Nextera Mate Pair Library Prep Kit (Illumina, San Diego, CA, USA). Due to the limited input DNA amount of 1 μg, the library was not additionally size-selected by, for example, Blue Pippin or SAGE Science. Sequencing-by-synthesis was performed on a HiSeq 3000 with 2 × 150 bp paired-end reads at the Max Planck Genome Centre Cologne, Germany.

### Illumina paired-end sequencing of ribosomal-depleted cDNA from *O. elata*

Around 1 μg DNase treated total RNA of *O. elata* was sent for sequencing to the Max Planck Institute for Molecular Genetics (Berlin, Germany). The library preparation was done using Roche KAPA RNA HyperPrep with RiboErase (Roche Diagnostics GmbH, Manheim, Germany). Sequencing was performed on an Illumina HiSeq 4000 system (Illumina, San Diego, CA, USA) with 2 × 75 bp paired-end reads.

### PacBio sequencing of total DNA from *O. elata*

PacBio sequencing of total DNA of etiolated seedlings of *O. elata* was performed on a PacBio RS II sequencer (Pacific Biosciences, Menlo Park, CA, USA). For this, 5 μg of high molecular weight DNA (between 20 kb and 200 kb in size; see above) were used without further fragmentation to prepare five SMRTbell libraries with PacBio SMRTbell Template Prep Kit 1 (Pacific Biosciences, Menlo Park, CA, USA) according to the manufacturer’s recommendations. The libraries were additionally size-selected with BluePippin (Sage Science, Beverly, MA, USA) to enrich for molecules >10, 11 or 15 kb. Recovered libraries were again damage repaired and then sequenced on a total of 138 SMRT cells with P4-C2 or P6-C4v2 chemistry and by MagBead loading on the PacBio RSII system (Pacific Biosciences, Menlo Park, CA, USA) with 360 min movie length.

### *De novo* assembly of isolated mtDNA

Illumina paired-end reads were trimmed with SeqtrimNext v2.0.62 using the plugins ‘PluginIndeterminants’, ‘PluginLowQuality’ and ‘PluginSizeTrim’ (https://rubygems.org/gems/seqtrimnext). Before and after trimming, read quality was evaluated with FastQC (http://www.bioinformatics.babraham.ac.uk/projects/fastqc). Uncalled and low-quality bases were removed. The sff_extract software v0.3.0 (https://bioinf.comav.upv.es/sff_extract) was utilized for Roche 454 data to trim 454-specific sequencing adapters, remove low-quality bases and to convert sequence reads from SFF to FASTA format. Then, both pre-processed data sets (Illumina and 454) were used as input for four different *de novo* assemblers namely CLC, IDBA, MIRA and Newbler, with Newbler operating on 454 data as input only. CLC v6.00 (part of the CLC Genomics Workbench, https://digitalinsights.qiagen.com/) and Newbler version 2.9 were executed using their graphical user interfaces with default parameters. IDBA_UD (from now on named ‘IDBA’) v1.1.1 was performed with Illumina Paired-end reads (-r option), Roche 454 single-end reads (-l option) and a k-mer range between 30 (–mink) and 90 (–maxk) incremented by 10 (–step) (46). MIRA 4.0.2 was run with a reduced Illumina data set (4 mio. pairs), using job mode ‘genome, denovo, accurate’, setting the parameters option to ‘-GE:not = 20 -DI:trt = /scratch_local -NW:cac = warn -OUT:rtd = yes’, adjusting the template_size option to ‘200–450’ and incorporating the ancillary xml file generated by sff_extract (47).

For the *de novo* assembler evaluation, the definition of high-confidence contigs (HCC) differs to the one for the final pipeline: Sequences of a specific *de novo* assembler were blasted against the sequences of all other three *de novo* assemblers and needed to be found by all assemblers. In detail, a sequence was called found if 90% of the contig bases were covered by any number of (sub)-sequences of another assembler contigs having an e-value <1e-40. For the final pipeline, high-confidence contigs were identified based on read-mapping statistics and length criteria as follows, Illumina data were mapped against the assembled contigs using BWA v0.7.15 (48). SAMtools v1.4 was used to create, sort and index the alignment data in BAM format. Contig-wise coverage was estimated with coverageBed (49,50). HCCs were then defined as those with contig size >1 kb and coverage >3000x; for details see ‘Results’ section and Figure 3). This change of the HCC definition is necessary to be independent of an inter-assembler comparison and is possible by the upstream mitochondrial isolation protocol.

Afterwards, to build the mtDNA assembly graph, the Iterative Sequence Ends Identity Search (ISEIS) pipeline, developed here, was applied to the assembled contigs. The ISEIS core algorithm takes an all against all BLASTN search result (51) of the assembled contigs (all contigs including high and low quality contigs) and filters for significant end-to-end hits (hitting at the very end of contigs; for the *de novo* assembly evaluation a range of 300 bp at the ends for MIRA, IDBA and CLC, 600 bp for Newbler were tolerated) with at least 49 bp with the respective termini and orientation/strand combinations within the sequence alignments as mentioned in Supplementary Figure S1. By this, an adjacency list of linked contig ends is created. As entry points for an iterative breadth-first search for connected components, HCCs from above were allowed only. By starting the search on HCCs only, low-confidence contigs will be integrated into the connected component only if connected to HCCs. Otherwise, they are discarded. The obtained graph consists of contig termini as vertices connected via edges, (i) when on the same contig, and (ii) when connected other contigs via overlapping contig ends. As detailed in the text, from this undirected graph (from now on named ‘IDBA graph’) consecutive recombinogenic repeat pairs can be identified and the mitochondrial genome sequence reconstructed semi-automatically in the FASTA format. The R-package igraph was used for graph visualization (52).

### Manual graph curation

To obtain final graphs for the three investigated *Oenothera* species, a number of curation steps, also exemplarily illustrated in Supplementary Figure S2, were conducted within ISEIS as follows: (i) Removal of plastidial subgraphs: Plastidial contigs were identified by a BLASTN search against available *Oenothera* plastomes. Afterwards, plastidial vertices in direct contact to the mitochondrial subgraph were removed, rendering the remainder of the plastidial subgraph unavailable for the ISEIS algorithm. (ii) Contig correction: Blast outputs were inspected for contigs of which vertices have only one or three or more edges. Illumina raw reads were screened for reads harboring contig ends’ blast hit sequences and contig sequences were corrected accordingly. (iii) Removal of small and low-coverage contigs: Contigs, which are so small (smaller than doubled k-mer size used for assembly) that they are fully covered by their neighboring contigs or have a low coverage and therefore likely originated from the nucleus, were removed. (iv) Correction of falsely connected contigs with Illumina Mate Pair and PacBio data: Grey edges in *O. elata* were removed, where the Illumina Mate Pair and PacBio data do not corroborate the connectivity of the participating contigs. (v) Simplified graphs: To clearly differentiate between repetitive and non-repetitive structural units within the graphs, stretches of contigs where both vertices of each contig have only two edges to other vertices (from which one edge goes to the second vertex of the same contig) were collapsed.

### Naming conventions

*De novo* assembly contigs generated by IDBA were renamed using the abbreviations berS (*O. villaricae*), suavG (*O. biennis*) and johSt (*O. elata*) for the different strains as introduced at the beginning of the Methods section. Contigs of IDBA were sorted by length in descending order and indexed in ascending order. PCR primers and Southern blot probes were named using the indices of the corresponding contigs for which they were designed for.

### Validation of mtDNA enrichment

To assess the level of nuclear and chloroplast DNA contamination in the isolated mtDNA samples, paired-end data were mapped with BWA v0.7.12 (53) against the complete set of contigs generated by the IDBA *de novo* assembler of all three species respectively. For coverage analysis, SAMtools v1.4 was used to create, sort and index the alignment data in BAM format as well as to generate read counts per contig statistics using idxstats (54). Contigs that were part of the final IDBA graphs were defined as mitochondrial. Plastidial contigs were identified via BLASTN (55) by comparing them to the available chloroplast sequences (*O. elata*, *O. biennis* and *O. villaricae*; GenBank accessions: AJ271079.4, EU262889.2 and KX118606.1, respectively). The remaining contigs were determined as derived from the nucleus.

### Pairwise BLASTN alignments to identify sequence homologies

Pairwise BLASTN alignments were performed using Circoletto v15.10.12 (56), a wrapper program executing legacy NCBI blastall v2.2.26 with default parameters (https://www.ncbi.nlm.nih.gov/books/NBK279671) and visualizing the BLASTN outcome with Circos v0.62.1 (57).

### Genome-wide repeat analysis

In parallel to the identification of repeats resulting from double forks, genome-wide repeat analysis was conducted relying on ROUSfinder v2 (58). Recommended parameter settings were adopted and repeats of length ≥50 bp were identified and collected for all three *Oenothera* species. To determine size ranges for long, intermediate, and small-sized repeats, k-means clusters were computed using R, applying Euclidean distance and three centers for each *Oenothera* species, separately.

### Stoichiometric analysis of recombinatoric repeats at the individual double forks

Nextera tagmentation adapters were removed from the mate-pair data of the isolated *O. elata* mtDNA using Nextclip v1.3 (59). Remaining clipped reads were aligned afterwards with BWA v0.7.15 (53) against the contigs of the *O. elata* IDBA graph. SAMtools v1.4 (54) was then used to create, sort and index the alignment data in BAM format. Only pairs, for which both mates map to different contigs, were counted and kept for further analysis. As the contigs within the IDBA graph can vary in size between hundreds of bases to many dozens of kilobases, the mate-pair fragments can span more than one contig by their large insert size. To overcome this issue, so-called contig chains were defined by extending the contig-repeat-contig (CRC) at both ends till the next occurring recombinogenic repeat pair (RRP) within the graph (Supplementary Figure S3). With this approach, it is possible to count the number of reads spanning one of the four CRC combinations for each identified ‘double fork’ within the IDBA graph.

In addition to the stoichiometric analysis of the mate-pair data set, PacBio long-reads generated from a total DNA library from *O. elata* (see above) were taken to calculate the stoichiometric distribution among the different CRC combinations. PacBio-specific bax files containing the information of the polymerase reads were converted with bax2bam to generate a SMRT Link pipeline v5 (Pacific Biosciences, Menlo Park, CA, USA) or higher compatible input. PacBio circular consensus sequences (CCS) were called with pbcss from the polymerase reads to correct for PacBio-specific errors (mostly 1 bp indels) with the following relaxed parameters: –minPredictedAccuracy 0.75, –maxDropFraction 0.5, –minPasses 0. CCS bam files were then converted to FASTA format using bam2fasta to obtain a BLAST compatible sequence format. The programs bax2bam, pbccs and bam2fasta are part of SMRT Link v5.0.1 program suite (Pacific Biosciences, Menlo Park, CA, USA) used in this approach. Afterward, all CCS reads were blasted against all sequences included in the IDBA graph of *O. elata* employing NCBI blastall v2.2.26 (https://www.ncbi.nlm.nih.gov/books/NBK279671) with default parameters. Three different data sets were filtered from the overall BLASTN outcome remaining only hits >100, 170 and 180 bp, respectively, for further analysis. These different subsets of BLASTN hits is necessary to reduce short hit contamination (100 bp) and to get an estimator of cross-mappings to contig ends of the other CRC combinations (170 and 180 bp) in the tab-delimited output table.

To identify and count CCS reads, which are consistent with our model of the IDBA graph and, in particular, fit our predicted CRCs, three steps were essential: (i) As a CCS read can be hit by more than one IDBA-graph contig, resulting in an unsorted multi-row entry, the BLASTN outcome was sorted by the CCS read identifier and the start position of the query sequence where the hit was positioned; bash command: sort -k1,1 -k7,7n blastfile > sorted.blastfile. (ii) To facilitate a straightforward search for each CRC string (e.g. ‘contig_1,repeat_4,contig_2’), BEDTools groupBy v2.20.0 (50) was applied to group the BLASTN outcome by the identifier of each CCS read. By this, the multi-row entry can be condensed into one row by summarizing the following columns with a specific operation in brackets: hit id (collapse), hit id (count_distinct), alignment length (sum) and length of IDBA graph contig (distinct); bash command: groupBy -i sorted.blastfile -g 1 -c 2,2,4,13 -o collapse, count_distinct,sum,distinct > collapsed.blastfile. (iii) Finally, a custom Perl script was implemented to collapse identical neighbored IDBA-graph contig identifiers, as both, the ‘collapse’ and the ‘distinct’ operation of BedTools cannot conduct this task. ‘collapse’ on the one hand just concatenates the identifiers (even if neighboring identifiers are the identical) whereas ‘distinct’ does not preserve the order of contigs, which is essential for CRC identification in our approach. Lastly, the percentage of covered CCS sequence by the IDBA graph contigs was calculated. Only those CCSs were kept whose sequences fully align to the IDBA graph contigs, and, to deal with PacBio sequencing errors, have at least 95% identity and are 5 kb long. CCS reads considered to be of nuclear origin we excluded from further analysis (Supplementary Figure S4). The final, condensed output was then screened by using the Linux command ‘grep’ for the comma-separated CRC-identifier string (example see above) and counted.

### Mitochondrial genome annotation, visualization

Mitochondrial genome sequences were annotated with a complex annotation scheme, illustrated in Supplementary Figure S5 and organized in three stages: (i) initial data generation, (ii) filtering and cross validation between the generated datasets, and (iii) merging filtered and validated data. Tools, which are available at chlorobox.mpimp-golm.mpg.de, assisted in the process of annotating organellar genomes (GeSeq), converting between different file formats (GBSON, a GenBank JSON converter), drawing organelle genome maps (OGDraw) as well as preparing GenBank files for NCBI submission (GB2sequin).

The first stage of the annotation scheme corresponded to the creation of the different datasets which focus on different genome feature types (protein coding genes, pseudogenes, tRNAs, rRNAs as well as open reading frames) from distinct organelle origin (plastid and mitochondrion). First, GeSeq v1.82 was applied on the mitochondrial input sequences in two different ways (60). For the annotation of the mitochondrial genes (GeSeq Mt run), six land plant species were selected covering a variety of angiosperms from within the rosids clade (*Arabidopsis thaliana* NC_037304.1, *Geranium maderense* NC_027000.1 and *Vitis vinifera* NC_012119.1), including the only two species of the myrtales order from which the mitochondrial genomes are known (*Eucalyptus grandis* NC_040010.1, *Lagerstroemia indica* NC_035616.1) as well as a gymnosperm (*Cycas taitungensis* NC_010303.1) as an outgroup. In the same GeSeq run, sequences of the recombinogenic repeats were uploaded as FASTA Nucleotide and tRNA *de novo* prediction with tRNAscan-SE v2.0.5 (Lowe and Chan, 2016) was activated. Plastidial pseudogenes were identified in a second run of GeSeq (GeSeq Pt run) using the respective plastidial genome of the three investigated *Oenothera* species (*O. villaricae* KX118606.1, *O. biennis* EU262889.2 and *O. elata* AJ271079.4) as database. rRNAs were identified by a simple BLASTN search with default parameters but allowing only the best hit using the NCBI entries X61277.1 (rrn5 and rrn18) as well as X02559.1 (rrn26) as queries. An rRNA-depleted Illumina paired-end RNA-seq dataset of *O. elata* was used to construct an RNA editome, to evaluate all exon-exon boundaries of the GeSeq-based gene predictions as well as to generate an expression profile. For that, the RNA-seq data were mapped against the final mitochondrial sequence of this species using STAR v2.7.0a (61). To generate an unbiased mapping result, the previously GeSeq-generated annotation was not included during the genome indexing step of STAR (–genomeSAindexNbases 8 –genomeChrBinNbits 18) nor used in the mapping step itself. Nevertheless the coverage of each GeSeq-determined exonic position was computed applying samtools mpileup with the -l option on the exon entries within the GeSeq annotation file only. In parallel, instead of counts per gene, the mitochondrial genome was segmented into 250 bp pieces using windowMaker from the BEDTools suite (50). Afterward, coverageBed was applied on the generated 250 bp segments bed file and the alignment bam file to count reads per segment. Single-nucleotide polymorphisms (SNPs) were called using freebayes v1.0.2 with default parameters, annotated with snpEff v4.3k (62) and filtered exclusively for C > T and G > A SNPs. Open reading frames were predicted using ORFfinder v0.4.3 with default parameters but allowing only ATG as start codon (-s 0).

In the second stage of the annotation scheme, the focus lay on filtering of and on cross-validation between the generated datasets. For single-exonic genes predicted open-reading frames were intersected with the CDS entries of the GeSeq Mt run using intersectBed allowing only those intersections that have the same start and end positions (-f 1 -F 1). Genes that fulfill these criteria were instantly tagged as verified protein-coding genes. For all other genes, where the ORFs were longer or shorter than the BLAT hits, these ORFs were fed into a BLASTP search on NCBI and in parallel examined within the IGV viewer v2.5.3, for example, if stop codons were present within the BLAT hit regions (63). This visualization approach was also used for the evaluation of the exon-exon boundaries of the multi-exonic genes taking the mapped RNA-seq data into account. For the trans-spliced genes, additionally available data at NCBI as well as the information within the corresponding papers were considered as follows: *nad1* (AH003143.2, (17)), *nad2* (AH003694.2, (64)) and *nad5* (exon a & b: X07566, exon c: X60046.1, exon d and e: X6004691, (65)). Because of the shortness of exon c of nad5, this sequence was separately searched on the mitochondrial genomes using BLASTN v0.2.26 with an e-value cutoff of 1e-03. Within the tRNAscanSE-predicted tRNAs, only those were considered to be true, which have a score ≥30, have no undetermined anti-codon (trnNull-NNN) and have not a fragmented BLAT hit by GeSeq. To distinguish between plastidially and mitochondrially originating tRNAs, tRNA entries of both GeSeq runs were intersected via intersectBed. Those intersections, where the plastidial locus is covered 100% by the BLAT search itself, were defined to be derived from plastidial origin and all other tRNAs were considered to be of mitochondrial origin. A similar approach was used to discriminate between mitochondrial and plastidial pseudogenes. Only those BLAT hits from the GeSeq Pt runs that do not overlap with genes from the GeSeq Mt run or are shorter than there corresponding BLAT hits from the GeSeq Mt run were designated to be plastidial pseudogenes. Intersected BLAT hits, where the BLAT hits coverage is low in both GeSeq runs, were tagged as mitochondrial pseudogenes.

In the third stage of the annotation pipeline, filtered and cross-validated datasets were collected and re-processed in different ways: All decisions made by all the different investigations within both GeSeq runs were collected and used as input in a custom-developed Perl script in order to manipulate the initial json files that were generated during the GeSeq runs. Likewise, for the RNA editome, a custom Perl script was written to extract all relevant information from the VCF file created by snpEff to generate the Supplementary Table S5 as well as a json file. All three json files (GeSeq Mt and Pt run as well as RNA editome) were combined to serve as input for the GBSON to GenBank converting tool.

The finalized annotation was used to create a mitochondrial genome map using OGDRAW v1.3 (66,67) with a user-defined configuration XML file to include identified plastidial pseudogenes. In parallel, the final GenBank annotation file was again processed using GB2sequin (68) for the NCBI submission.

## RESULTS

### The metagenome assembler IDBA yielded best results for the mtDNA assemblies performed in this study

To reduce the complexity of the input DNA, we generated our sequencing data from highly pure mitochondrial DNA (mtDNA), enriched by cell fractionation (Materials and Methods section, and below). The intent was to reduce possible contaminations by nuclear mitochondrial DNA (NUMTs; (69)), i.e. segments of mitochondrial genomes, translocated into the nucleus that are commonly present throughout the plant kingdom. Two next generation sequencing (NGS) data sets were generated, Illumina paired-end and Roche 454 single-end. The sequencing data of *O. villaricae* were used to perform a *de novo* assembly evaluation of four different assemblers: CLC (Qiagen), IDBA (46), MIRA (47) and Newbler (Roche). Those were chosen to cover a wide range of implemented algorithms (OLC (Newbler) and De Bruijn graph (IDBA)), data input options (stand-alone (Newbler) or hybrid assemblers (CLC, IDBA and MIRA)), availability (open source (MIRA and IDBA) and commercial software (CLC and Newbler)), and area of application (i.e. meta-genomics, IDBA). The raw assembly output ranged from 14 contigs (Newbler; cumulative genome size of 424 082 bp) to 847 contigs (CLC; 969 165 bp), 883 contigs (IDBA, 1 057 763 bp) and up to 1540 contigs (MIRA; 1 243 023 bp).

To assess, which assembler creates valid contigs, i.e. contigs whose sequences overlap at their ends, an all-against-all sequence alignment was generated with BLASTN. The BLAST output then served as input for our ISEIS algorithm that represents the first step of the SAGBAC pipeline. The starting points for the first iteration were so-called high confidence contigs (HCC), which (for the *O. villaricae* data set) we defined as contigs produced by all assemblers. We identified these contigs in the BLAST-result table and compared their termini to the termini of the remaining ones to find sequence overlaps. In the second iteration, we then searched for overlaps of these new contigs with the contigs left in the BLAST table. The iterative process proceeded until no overlaps to any new contigs were found. As a result, an adjacency list of linked contig termini was created, which can be visualized as a graph with suitable R packages, such as igraph (52). It should be emphasized that the only exit criterion for the data-driven ISEIS algorithm is the absence of any new overlaps between contig ends. Thus, the outcome can be a linear/chromosome-like or a circular graph. The resulting graphs, obtained from the four *de novo* assembly raw outputs, differ considerably (Supplementary Figure S6). CLC yielded almost exclusively isolated contigs (HCCs without any sequence overlap with other contigs). Using Newbler, all contigs were connected at least once, although some contig termini remained unconnected. MIRA harbors most contigs within its graph. However, it yielded a large number of unconnected contig termini, leading to a complexity that can no longer be inspected by eye. Only IDBA, conceptually designed to assemble meta-genomes, was able to generate a set of contigs that was fully connected to each other. Strikingly, it produced a circular graph. We, therefore, continued to work with IDBA and further generated the IDBA assemblies for the other two *Oenothera* species *O. biennis* and *O. elata*.

### *Oenothera* mtDNA sequence reads assemble into circular graphs

**Figure 2.**
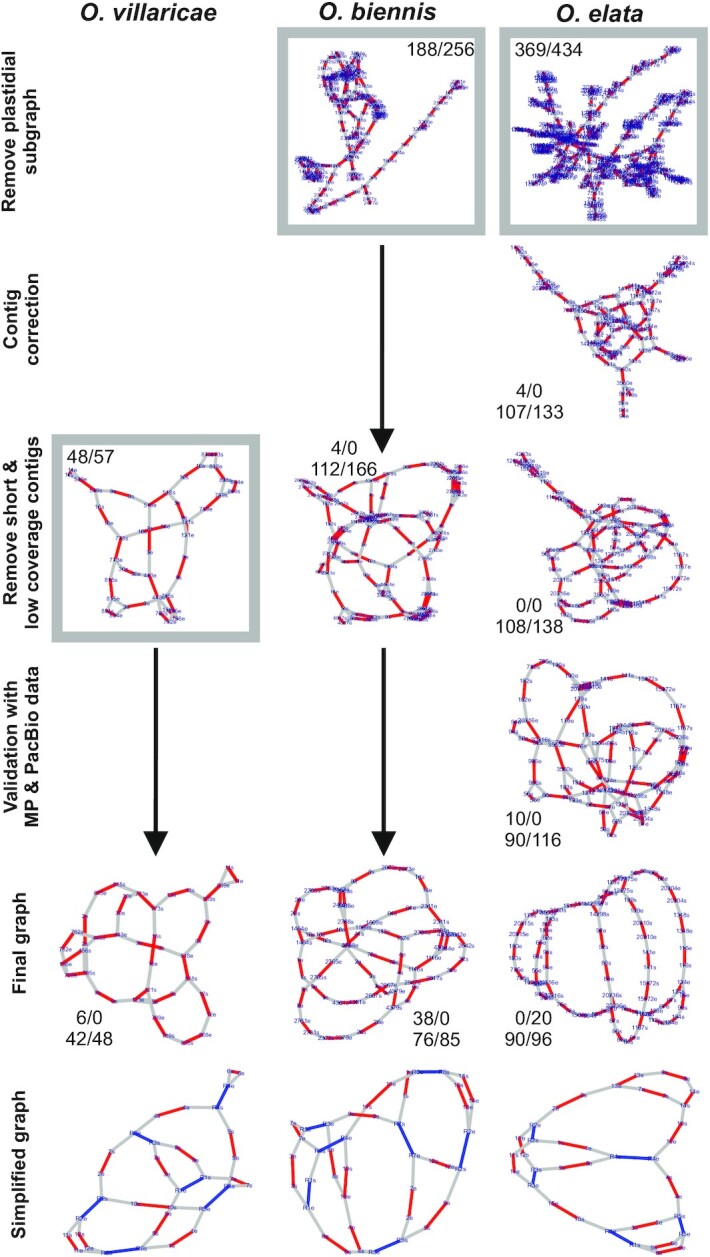
Graph curation pipeline from raw to final graphs for the IDBA assemblies of three *Oenothera* species. For each IDBA assembly, an adjacency list is generated by the ISEIS algorithm that can be used for the construction of an undirected graph. Each contig is defined by two vertices (s = start, e = end) linked by a red edge. An overlapping event between two different contig ends is represented by a gray edge. Blue edges represent the identified recombinogenic repeat pairs. Gray boxes show the start point at which each raw IDBA graph is starting within the curation pipeline. Count pairs within the gray boxes reflect the number of vertices/edges before any curation step. Black arrows illustrate the skipped steps, which are not necessary for that species. First count pair at each curation step shows the number of vertices/edges that were excluded from the step before, resulting in the second count pair of vertices/edges.

Comparing the raw assemblies of the three *Oenothera* species, it is conspicuous that the number of contigs ranges considerably, from 883 for *O. villaricae*, to 4381 for *O. biennis*, to 20 317 contigs for *O. elata*. The sum of all contig sizes ranged from 1 057 763 bp to 2 346 337 bp and 18 986 762 bp, respectively (Table 1). In both assembly metrics, the numbers differed by one order of magnitude for *O. elata* compared to *O. villaricae* or *O. biennis*. As discussed below, this is a result of a much higher contamination of the initial DNA in *O. elata*, containing segments of the nuclear and chloroplast genomes. Unlike the case for *O. villaricae*, the initial IDBA assemblies of *O. elata* and *O. biennis* (this time HCCs were defined by read coverage (3000×) and contig length (1 kb); see Materials and Methods for details), no circular graph structure was obtained. We therefore compared the IDBA graphs to their respective plastid genomes available from NCBI (*O. elata*, *O. biennis* and *O. villaricae*; GenBank accessions: AJ271079.4, EU262889.2 and KX118606.1, respectively), as well as to the contigs that are part of the IDBA graph of *O. villaricae*. From this, it was evident that plastidial contigs were integrated/connected within/to the two graphs and that some misassemblies have occurred. Curation of the IDBA graphs included (i) removal of plastidial subgraphs, (ii) correcting obvious misassemblies, (iii) removing small and low coverage contigs and (iv) falsely connected contigs by removing affected edges from the graph. The filtering steps are illustrated in Figure 2. After curation, the number of contigs were 21 for *O. villaricae*, 38 for *O. biennis* and 45 for *O. elata*, respectively, with respective cumulative length of 408 260, 424 132 and 418 451 bp, respectively, in line with published size estimations of the *Oenothera* mitochondrial genome of approximately 400 kb (70).

**Table 1.** Descriptive statistics for the assembly, annotation and RNA editing outcomes

	Metric	*O. villaricae*	*O. biennis*	*O. elata*
**Assembly**	raw assembly [# contigs]	883	4381	20 317
	raw assembly [bp]	1 057 763	2 346 377	18 986 762
	IDBA graph [# contigs]	21	38	45
	IDBA graph [size]	408 260	419 446	418 451
	Reconstructed Master circle [bp]	408 744	424 132	449 216
	GC content	46.66%	46.70%	46.83%
**Annotation**	Protein coding genes	35		
	tRNA	14 (Mt origin)		
		7 (Pt origin)		
	rRNA	3 (3 unique)		6^a^ (3 unique)
	Coding genes with introns	8		
	of which are trans-spliced	3		
**RNA editing^1^**	# RNA editing sites	NA	NA	681
	on genes	NA	NA	511
	Non-synonymous	NA	NA	472
	of which have gained a pre-mature stop codon	NA	NA	3
	on double edited codons	NA	NA	22 (11)
	synonymous	NA	NA	39

^1^RNA editome available for *O. elata* only, as only for that species, rRNA-depleted Illumina data were available.

^a^All three mitochondrial rRNAs are localized on the large repeat, which was identified in *O. elata*. NA = not available, Mt = mitochondrial, Pt = plastidial.

### Read coverage of the IDBA graph supports mitochondrial origin

**Figure 3.**
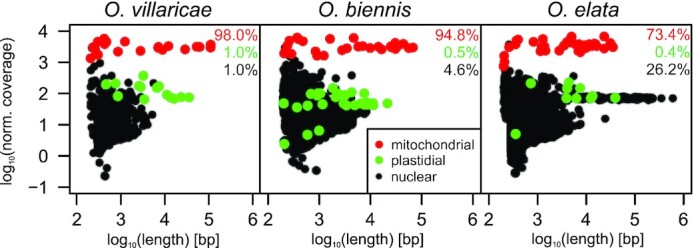
Length-normalized coverage distribution among IDBA contigs for three *Oenothera* species. Shown are the contig-length-normalized coverage distributions for the IDBA contigs after mapping the Illumina paired-end reads to them and as a function of contig length. Colored percentages indicate the overall distribution of reads to each genomic compartment, Black = nuclear; green = plastidial; red = mitochondrial.

As previously mentioned, the NGS libraries used for the assemblies were derived from highly pure mtDNA obtained by cell fractionation. Due to an improved triple Percoll sucrose gradient, the employed mitochondrial fractions were largely devoid of chloroplast and nuclear DNA contaminations ((24) and Materials and Methods for details). Consequently, the contigs integrated into the final IDBA graphs have one or even two orders of magnitude higher coverage than most of the remaining contigs of the assemblies, which, therefore, likely represent the still present residual contamination that was not integrated into the graphs. For the chloroplast genomes, this was verified by BLAST analyses against the corresponding plastome sequence present in NCBI (AJ271079.4, EU262889.2, and KX118606.1; also see above). All other contigs that were neither found in the IDBA graph nor mapped against the plastid genome, were considered nuclear DNA contamination (Figure 3).

### IDBA graph contains the earlier published *Oenothera* mtDNA sequences

As another checkpoint of our analyses, we checked to what extent already published sequences of the *Oenothera* mtDNA were found within the IDBA graph. Already historically very early, particular efforts were invested into the mitochondrial genome of *O. villaricae* (71) and first mitochondrial genes were identified therein (72,73). Therefore, a comparison between these sequences and our IDBA contigs of *O. villaricae* was performed. After downloading available mitochondrial sequences for *O. villaricae* from NCBI (nuccore database entries for taxonomic ids 3941 and 3950), a BLASTN search was performed to compare the IDBA contigs of *O. villaricae* to the 44 retrieved sequences. Strikingly, all of them could be mapped partly or completely to the contig sequences, which are included in the IDBA graph (Supplementary Figure S7). Contigs berS_2 (110 274 bp), berS_3 (109 856 bp) and berS_5 (61, 574 bp) were covered most, which are the largest contigs within the IDBA *de novo* assembly. The sequences AH003143.2 and AH003694.2 map to more than one locus and on different contigs, which is not surprising, as they both represent the trans-spliced *nad1* and *nad2* genes, for which the exonic sequences were concatenated by stretches of 100-bp-long Ns (17,64). Additionally, four of the six repeat pairs, identified in *O. villaricae*, could be found among the NCBI sequences.

### Identification and comparison of repetitive elements in the mitochondrial genome of *Oenothera* species

Using the obtained simplified graphs shown in Figure 2 as a starting point, the recombinogenic repeat pairs (RRPs) could now be easily identified upon visual inspection. Their number varies between the three species. *O. biennis* harbors seven and *O. biennis* six repeat pairs, while *O. elata* contains only five repeat pairs (Table 2). RRPs were assigned to large, intermediate and small-size repeat categories by applying a k-means clustering on a genome-wide repeat analysis output (Supplementary Table S3). Three RRPs were assigned to the long-size repeat category ranging between 825 and 1625 bp, 13 RRPs were grouped to the intermediate-size repeat category ranging between 239 and 479 bp, and two RRPs were related to the small-size repeat category with 171 and 179 bp. In the three species, the contigs berS_121, suavG_152, and johSt_3550 represent the long-size repeats. Those are considered to confer frequent and reversible recombination events that lead to a simultaneous presence of master- and smaller sub-circles (74). Each of the three *Oenothera* species has its own unique long-size repeat. Furthermore, we found seven intermediate-size repeats of which three are shared among all species (Table 2, marked bold). Mitochondrial intermediate-size repeats have been found to recombine infrequently but are believed to be part of the break-induced replication pathway (BIR) and can lead to increased complexity of mtDNA (75). Next, we validated the proposed structure by independent wet lab and bioinformatics analyses.

**Table 2.** Recombinogenic repeat pair sets of three *Oenothera* species

Repeat type	*O. villaricae*	Length [bp]	*O. biennis*	Length [bp]	*O. elata*	Length [bp]	gene^c^
LSR	berS_121	1337	suavG_4381 ^a^	1316	johSt_1348 ^b^	1352	*atp9*
	p.o.c.		p.o.c.		johSt_1564	1151	*atp6*
LSR	p.o.c.		suavG_152	1625	p.o.c.		
**LSR^1^/ISR^2^**	**berS_443^2^**	**475**	**suavG_1116^2^**	**479**	**johSt_3550^1^**	**825**	*nad6*
	**berS_795^2^**	**239**	**suavG_2311^2^**	**239**			
ISR	berS_518	432	p.o.c.		p.o.c.		
**ISR**	**berS_539**	**421**	**suavG_1464**	**397**	**johSt_12875**	**397**	
ISR^2^/SSR^3^	berS_773^2^	300	suavG_2483	201	johSt_20315	201	
			suavG_2788^3^	179			*rpl2*
			suavG_2457	206	johSt_20310	206	
ISR	p.o.c.		suavG_1599	370	johSt_14298	370	
ISR	p.o.c.		suavG_4379	260	johSt_20236	261	*atp1*
SSR	p.o.c.		p.o.c.		johSt_20316	171	*nad5*
sum	1 LSR +		1 LSR +		1 LSR +		
	5 ISR		5 ISR +		3 ISR +		
			1 SSR		1 SSR		

Listed are all recombinogenic repeat pairs (RRP) identified in three different *Oenothera* species and their occurrence within the three species. Underlined: sequence remained only once in the IDBA-graph as a singleton contig; Bold rows: shared repeats among all three *Oenothera* species. LSR = Long-size repeat; ISR = Intermediate-size repeat; SSR = Small-size repeat; p.o.c. = sequence is present only once, which is part of another contig. ^a,b^Note, contig suavG_4381 and johSt_1348 originally consist of four and two contigs, respectively, that were concatenated to be comparable to the other two respective species in this table. ^c^listed are genes, which are extending into the RRP, or the RRP is, as a whole, part of the gene.

### PCR and Southern blots validate the *in silico* model

**Figure 4.**
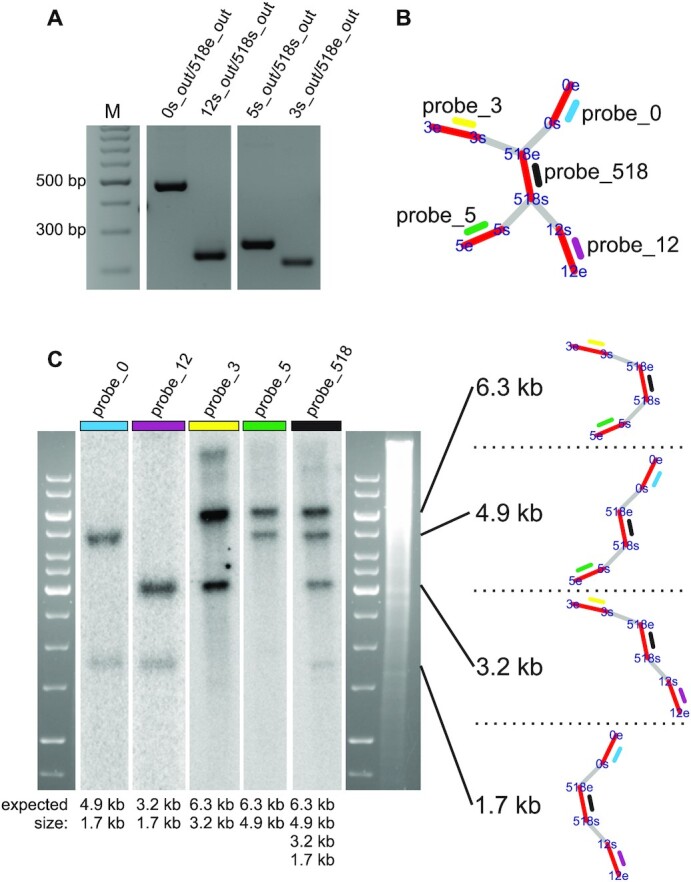
PCR and Southern blot analysis for verification of the predicted mitochondrial genome structure. (**A**) PCR experiment; *in vitro* fragments for the repeat berS_518 and its adjacent contigs are matching the expected sizes (from left to right: 465 bp, 226 bp, 266 bp and 219 bp). This example is representative for all tested contig overlaps. (**B** and **C**) Southern blot experiment; Genomic DNA was digested with HindIII for berS_518. Probes used for hybridization and where they are located within the IDBA graph are illustrated in (B). From which CRC each DNA fragment is derived after digestion, including their sizes, is shown to the right of the Southern blot. Probe_0, _3, _12, and _5 result in two expected fragments, whereas probe_518 appears in all four possible variants as expected.

As introduced in Figure 1, each RRP in the IDBA-graph model is connected to four different contigs, two at either terminus, together building a ‘double fork’. Rearrangement events can potentially lead to four different variants of combined contigs, so-called contig-repeat-contig (CRC) combinations for every RRP (Figure 1C). To test the existence of all four possible CRC variants in *O.villaricae*, we performed PCR experiments targeting all six identified RRPs, and, in addition, Southern blot experiments analyzing one of the RRPs. Exemplary results of both, PCR and Southern analysis, can be found in Figure 4 for the ‘double fork’ berS_518.

In more detail, for the PCR experiments, primer pairs were designed that either span one of the four contig-repeat boundaries (CRC), or both primer mates target the unique sequences of a CRC, fully spanning the repeat (CRC) (Supplementary Table S1). In all cases, the PCR amplification was tested via gel electrophoresis and resulted in distinct DNA bands that correspond well to the expected fragment lengths. These results show that the overlaps between contigs and repeats (CRC) as well as all adjacent contig combinations (CRC) exist *in vitro*.

For the Southern blots, and as calculated from the *in silico* model, we expected fragments of 1.7 and 4.9 kb when berS_518 was analyzed in a HindIII digest. The two fragments were detected by hybridization to the two CRCs to probe 0: HindIII - berS_0 – berS_518 – berS_12 - HindIII (1.7 kb) and HindIII - berS_0 – berS_518 – berS_5 - HindIII (4.9 kb; Figure 4C). In the first lane of the blot, two bands of the expected size were obtained (Figure 4C). For probes 3, 12 and 5, sizes of expected and verified sequence variants aligned as well. Lastly, probe 518 was designed directly on the repeat and should detect all four possible sequence combinations (1.7, 3.2, 4.9 and 6.3 kb), which is indeed the case. In summary, both wet lab techniques confirmed the graph-derived *in silico* model of the *O. villaricae* mtDNA.

### Recombinogenic repeat pairs exist in different stoichiometries

To further substantiate the experimental validations of the predicted CRC configurations of all RRPs, and to bring them into a stoichiometric context, we performed the following analyses. We used mtDNA-enriched Illumina mate-pair (5 kb insert size) and total DNA PacBio RSII (size selection > 5 kb) data that were available for *O. elata*. To be able to calculate read counts for the four CRCs of each RRP, a developed custom data processing pipeline was used to overcome the peculiarities of both datasets after mapping them to the contigs in the final IDBA graph of *O. elata* (for details see Materials and Methods).

Table 3 summarizes the stoichiometric analysis and statistics, specifically assessing the relative abundance of all four alternative CRCs for every given RRP. Theoretically, two groups would be possible, known from the literature: Group 1, in which all four CRCs occur in equal or gradual proportions, and group 2, in which two out of four are equally abundant, with the other two CRC configurations being underrepresented, but still existing to an appreciable degree. These two groups normally correspond to large frequent and intermediate-sized infrequent recombining repeats respectively. In *O. elata*, we only observed ‘group 2’ RRPs, regardless of their size (Table 3).

**Table 3.** Stoichiometric contig-repeat-contig configuration statistics

Contig ID		johSt_3550	johSt_12875	johSt_14298	johSt_20236	johSt_20316
Length [bp]		825	397	370	261	171
Conf 1	Illumina	346	3293	569	28 545	731
	[%]	1	5	1	47	2
	PacBio	60	12	5	943	0
	[%]	4	1	0	52	0
Conf 2	Illumina	15 538	27 852	28 789	829	24 142
	[%]	49	45	50	1	50
	PacBio	579	882	958	8	585
	[%]	39	47	52	0	52
Conf 3	Illumina	14 865	29 759	26 064	717	23,248
	[%]	47	48	45	1	48
	PacBio	736	994	890	5	545
	[%]	50	52	48	0	48
Conf 4	Illumina	966	556	2,606	30,799	255
	[%]	3	1	4	51	1
	PacBio	93	6	4	872	0
	[%]	6	0	0	48	0
Read Sums	Illumina	31 715	61 460	58 028	60 890	48 376
	PacBio	1468	1894	1857	1828	1130
Usage	Illumina	1.5^a^	2.8	2.7	2.8	2.2
Factor	PacBio	2.2	2.8	2.8	2.7	1.7

Total read statistics as well as the read frequency distributions for all contig-repeat-contig (CRC) configurations (conf) of each recombinogenic repeat pair (RRP) found in *O. elata* spanned by Illumina Mate-Pair and PacBio long reads. Highlighted green are the two most abundant (dominant) CRCs of each RRP. Usage factor is defined by dividing each sum of total reads by the lowest number observed across all CRC-candidates for each data set (Illumina = 21 865; PacBio = 665; Note that two CRC-candidates were dismissed as false-positives.). ^a^Illumina usage factor is 1.5 instead of close to 2 for johSt_3550, as two contig chains consist of only one contig, after which, an RRP immediately follows (which is, by definition, the end of a contig chain).

Second, by summing up the reads of all four CRC configurations of each RRP, we could calculate the overall usage of each RRP. Depending on the RRP, it was found to range between 21 865 and 61 460 reads for the mate-pair dataset and between 665 and 1,894 reads for the PacBio dataset, respectively (Table 3). To determine whether recombination at some RRPs was more frequent than at others, read counts were normalized by dividing the total read number by the lowest read count that occurred in each dataset. This yielded the so-called ‘usage factor’. Overall usage showed an up to threefold difference between the RRPs, which allowed a grouping into two fractions (factor 2 and 3 means that this particular RRP is used twice or three times as often as other contigs). The usage factors are presented in Table 3 and are consistent for both data sets (Illumina and PacBio) for all RRPs.

### Mitochondrial genome reconstruction and genome comparison

As already alluded to in the Introduction and addressed already here as an important point of discussion needed for an understanding of our rationale, there is much debate over the existence of mitochondrial genomes of so-called master circles, with evidence mounting against their actual existence, at least not as a dominating variant (3–7). However, the master circle may still be a useful concept in that it represents a path through the network (consisting of contig and repeat termini as vertices, and edges representing contig-sequences or sequence overlaps) that, if possible based on sequence-overlaps, contains all contigs, thus representing the longest possible path through the network. This master circle can thus be seen as a maximum contents path through the network. It may not be, and we will encounter this situation below, a ‘simple path’ in graph-theory terminology, as vertices may have to be visited repeatedly in order to traverse all contigs in one path. Importantly, our approach is purely data-driven. Thus, if it is possible to trace out a path through all contigs and which leads to a closed, circular topology, then, at least in theory, such a master circle genome is possible. Therefore, we wish to qualify all results regarding master circle reconstruction to be seen in the light of primarily establishing the possibility of the master circle as a theoretical construct, irrespective of whether or not this master circle genome actually exists in reality.

**Figure 5.**
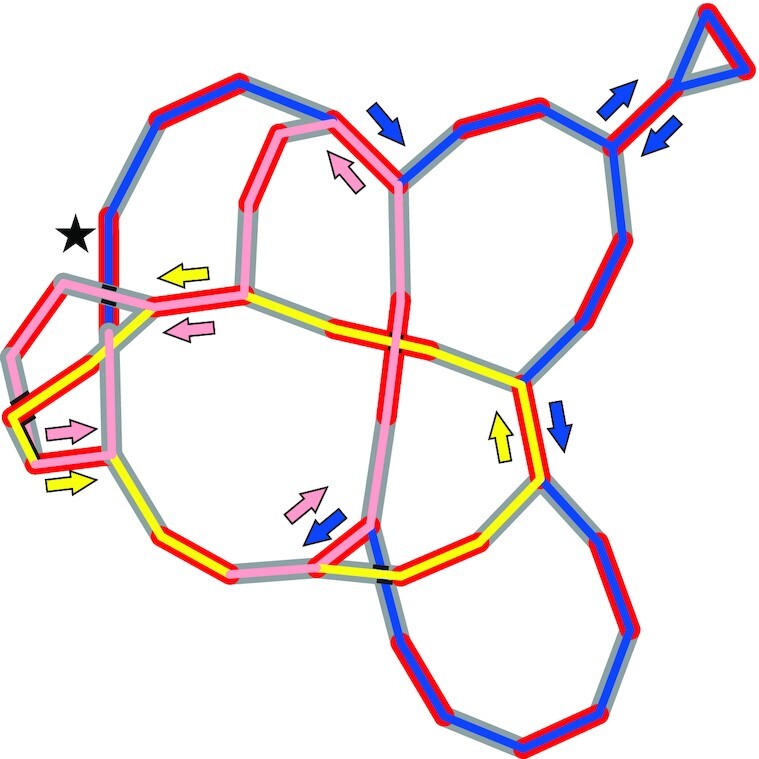
Master circle reconstruction in *O. villaricae*. Manual detection of paths through the graph in three steps following the arrows and using two rules: First, end the path at the same contig as you started and, second, each repeat should be traversed only twice. Path: Starting at the star-tagged contig following the blue path, then the yellow and finishing with the pink path ending at the second vertex of the star-tagged contig.

With that in mind, to obtain a master circle for the mitochondrial genome from the curated IDBA graph, two rules for traversing the graph were set initially. (i) While imposing circularity, start and termination vertex should be identical. (ii) Each repeat should be traversed at least twice. This can be easily realized for the graph of *O. villaricae* (Figure 5). However, the graphs of *O. biennis* and *O. elata* appeared to be more complex. To close the path for the *O. biennis* graph, a large contig needs to be traversed twice (suavG_41; 4703 bp). It was not identified as a repeat by the ISEIS algorithm because it is not embedded within a double, but within a single (one-sided) fork. In addition, one repeat (suavG_1599) was traversed thrice. For the graph of *O. elata*, it was necessary to pass a stretch of three contigs twice (johSt_124, johSt_67, johSt_126). In all cases, involved contigs have the highest read coverage when mapping the initial Illumina paired-end reads to them that were used for the *de novo* assembly (Supplementary Table S4). We are therefore confident that the proposed genome models are valid.

The sizes of the three reconstructed mitochondrial genomes range from 409 to 449 kb with an GC content between 46.66% and 46.83% (Table 1). This places them among the top-20 GC-rich species of the 323 mitochondrial land plant genomes published thus far (NCBI as of March 2022). A sequence alignment of all three genomes using AliTV (76) shows a nearly 100% sequence identity between *O. biennis* and *O. elata*, not taking structural variation into account, however. A comparison of *O. villaricae* with *O. elata* reveals that 12 kb (3.0%) of *O. elata* are unique to it, whereas 27 kb (6.0%) of *O. villaricae* are not present in *O. elata* (Supplementary Figure S8).

### Mitochondrial genomes contain the canonical set of mtDNA genes—gene identification by homology search, gene expression, and RNA editome

**Figure 6.**
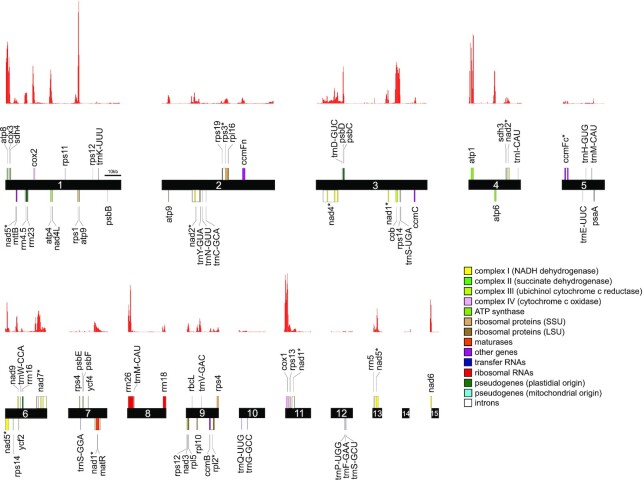
Gene map and gene expression atlas of the *O. elata* mitochondrial genome. A linear representation of the contigs from the simplified graph of *O. elata* is shown, generated by OGDRAW. All types of essential mitochondrial complexes, as well as mitochondrial and plastidial pseudogenes are described in the legend along with their color code. Histograms above illustrate gene expression along the mitochondrial contigs (coverage for each 250 bp genome segment).

Following the mitochondrial genome reconstruction, we employed GeSeq (60) for annotating all genomes. Besides determination of coding regions, this proved an important step to validate genome completeness. For this, a complex multi-staged annotation scheme was applied (Supplementary Figure S5) and annotations were visualized with OGDRAW (67) (Figure 6). First, we could show an even distribution of genes over the whole sequence of all three genomes and on both strands. Conserved genes, considered essential for the mitochondria genomes of most land plants, were found in all three investigated *Oenothera* species. Specifically, genes encoding the oxidative phosphorylation complexes (*nad1-6*, *4L*, *7*, *9*; *sdh4*; *cob*; *cox1-3*; *atp1*, *4*, *6*, *8*, *9*), all three rRNA genes (*rrn5*, *rrn18* and *rrn26*), and several ribosomal proteins (*rps1*, *3*, *4*, *13*, *14*, *19* and *rpl2*, *5*, *10*, *16*) were identified. Also, *mttB* (a membrane transport protein), *matR* (maturase), *ccm* (cytochrome *c* biogenesis), and additionally, 16 plastidial pseudogenes, psaA, psbB,psbC, psbD, psbE, psbF, rbcL, rps4, rps11, rps12, rps14, ycf2, ycf3, and all three plastidial rRNAs, were detected. Besides *nad1* and *nad2*, also *nad5* is trans-spliced (17,64,65). Twenty-four different tRNA genes were initially identified with tRNAscan-SE v2.0.5 (77). Four of them were identified as false-positives, reflected by their fragmented BLAT hits (see below); seven were of plastidial origin and translocated from this genome. The remaining 13 tRNA genes are of true mitochondrial origin, which could be confirmed by full-length BLAT hits, when blasted against the six chosen plant mitochondrial genomes (for details see material and methods). A list of all genes present in the *Oenothera* mitochondrial genomes is provided in Table 4.

**Table 4.** Gene content of all three investigated *Oenothera* mitochondrial genomes

Gene set	Members of the gene set				
Complex I	*nad1* (5*)	*nad2* (5*)	*nad3*	*nad4* (4)	*nad4L*
	*nad5* (5*)	*nad6*	*nad7* (5)	*nad9*	
Complex II	*sdh4*				
Complex III	*cob*				
Complex IV	*cox1*	*cox2*	*cox3*		
Complex V	*atp1*	*atp4*	*atp6*	*atp8*	*atp9*
Cytochrome C biogenesis	*ccmB*	*ccmC*	*ccmFC* (2)	*ccmFN*	
Ribosomal large subunits	*rpl2* (2)	*rpl5*	*rpl10*	*rpl16^b^*	
Ribosomal small subunits	*rps1*	*rps3*	*rps4*	*rps13*	*rps14*
	*rps19*				
Intron maturase	*matR*				
Protein translocase	*mttB^c^*				
Plastidial pseudogenes	*4.5S rRNA*	*16S rRNA*	*23S rRNA*	*rbcL*	*psaA*
	*psbB*	*psbC*	*psbD*	*psbE*	*psbF*
	*rps4*	*rps11*	*rps12*	*rps14*	*ycf2*
	*ycf4*				
rRNA	*5S rRNA (x3)^r,a^*	*18S rRNA (x2)^r^*	*26 rRNA (x2)^r^*		
Mt-originated tRNAs	*tRNA-Cys* (GCA)	*tRNA-Gln* (TTG)	*tRNA-Glu* (TTC)	*tRNA-Gly* (GCC)	*tRNA-Ile* (CAT)
	*tRNA-Lys* (TTT)	*tRNA-fMet* (CAT) (x2)^r^	*tRNA-Phe* (GAA)	*tRNA-Pro* (TGG)	*tRNA-Ser* (GCT)
	*tRNA-Ser* (TGA)	*tRNA-Tyr* (GTA)			
Pt-originated tRNAs	*tRNA-Asn* (GTT)	*tRNA-Asp* (GTC)	*tRNA-His* (GTG)	*tRNA-Met* (CAU)	*tRNA-Ser* (GGA)
	*tRNA-Trp* (CCA)	*tRNA-Val* (GAC)			

Genes with multiple exons are denoted with the number of exons shown in parenthesis, trans-spliced genes are indicated with an *. ^r^These genes are present in two copies in *O. elata* because they are located on the identified large repeat. ^a^A third copy of *5S RNA* is present outside the identified large repeat. ^b^*rpl6* carries an internal stop codon but can be transcribed using an alternative start codon GUG downstream from the ATG start codon. ^c^*mttB* lacks its normal start codon but an alternative GUG start codon can be created via RNA editing.

In parallel to the annotation based on homology searches or tRNA prediction, an rRNA-depleted Illumina paired-end RNA-seq data set was used to create an expression atlas (read coverage profile) along the mitochondrial genome of *O. elata* (Figure 6). When compared to the GeSeq annotation, all 33 779 unique exonic positions of protein-coding genes were covered by the RNA-seq data. This supports the high accuracy of the homology-search-based annotation, as well as that of the assembly.

Besides the expression profile, additionally, an RNA editome was generated (Supplementary Table S5). In total, 681 RNA editing sites were identified in *O. elata*, from which 511 are located in protein-coding genes. 472 non-synonymous editing sites lead to non-synonymous changes of amino acids, including three non-sense mutations (gain of stop codons) (*ccmF*CeU1315R*, *atp9*eU223R* and *atp6*eU844Q*). Thirty-nine synonymous RNA editing sites and 11 double-edited codons were observed.

### Prediction-based analysis reveals variability of mitochondrial genome variants

After the identification of all RRPs, the reconstruction of the master circle (see above for our comments on qualifying ‘master circle’ as possibly a hypothetical construct only), as well as resolving stoichiometry of recombinatoric events, our ultimate goal was to predict all possible rearrangements and all possible (sub)graphs. This was accomplished by using a new algorithm to estimate the structural diversity. In brief, the new algorithm is embedded into the SAGBAC pipeline and generates all paths through a graph considering the two rules, which were set previously, but with a slight difference. (i) Start and end the path at the same vertex, but use a different edge, and (ii), traverse each repeat no more than twice. Hence, the difference between this and the original algorithm to reconstruct the master circle from the graph is that it is not obligatory to traverse all repeats twice. Instead, sub-circles that lack some repeats and/or harbor only one repeat just as a singleton can be generated. Applying these rules, as many as 70 different graphs were predicted for *O. villaricae* (Supplementary Data S1, Supplementary Data S2). Of those 44 graphs represent the master circle in different configurations (all repeats twice, each of all other contig once), six small graphs/sub-circles (no repeat twice; some, but not all of the other contigs once) and 20 intermediate-sized graphs/sub-circles (all repeats at least once; some, but not all of the other contigs once).

**Figure 7.**
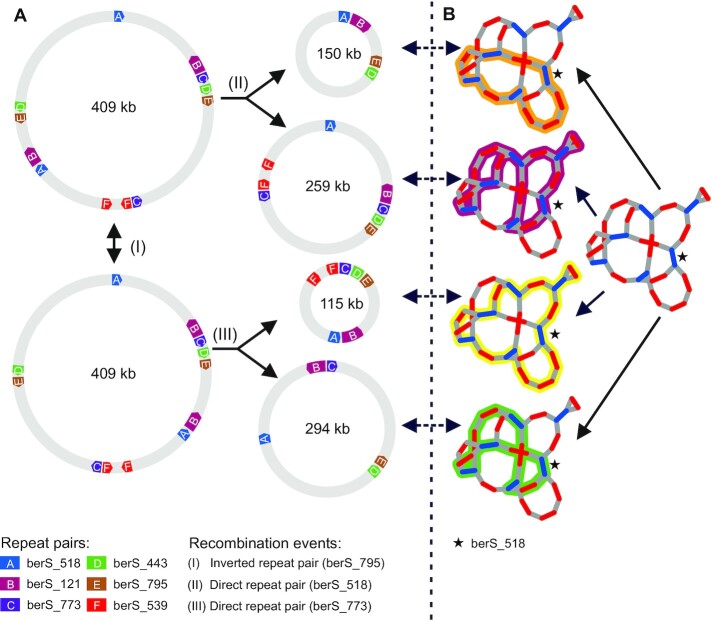
Examples for rearrangement events and their corresponding sub-paths within the IDBA graph of *O. villaricae*. Recombination at inverted repeats, like berS_795 (RRP_E), can lead to an inversion of the sequence between the two mates of the repeat (event I) and therefore to a new orientation of all mates of repeat pairs, which lie on that inverted sequence, which is true for berS_518 (RRP_A), berS_121 (RRP_B) and berS_773 (RRP_C) in that particular case. Direct repeats (here exemplary for berS_518 and berS_773) lead to the formation of two smaller sub-circles in event II (259 kb and 150 kb) and event III (115 kb and 294 kb). Circular maps were generated using AngularPlasmid. Proportions of repeat and contig sizes was altered to give a proper view of participating RRPs. (**B**) Illustration of the usage of the four different contig-repeat-contig (CRC) combinations for the identified repeat berS_518 (RRP_A) within the IDBA graph of *O. villaricae* leading to four different sub paths which correspond to one of the sub-circles (dashed arrows) generated in (**A**). Within the graphs, a pair of two vertices defines a contig (red edge) or a repeat (green edge) and an overlapping event between two different contig ends is represented by a gray edge. Black star: contig representing the identified repeat berS_518 (RRP_A).

How master- and sub-circles can emerge from each other is illustrated exemplarily in Figure 7. Sub-circles that derive from the four CRC configurations of berS_518 are displayed in Figure 7B and which recombination events are necessary to obtain them (Figure 7A): Sub-circularization of original master circle at berS_518 (event I), inversion of sequence between the berS_795 repeat pair (event II) of original master and down from that a sub-circularization at berS_773 (event III) leads to two other sub-circles.

### The *Oenothera* mitochondrial genome might contain loci for cryptic cytoplasmic male sterility

**Figure 8.**
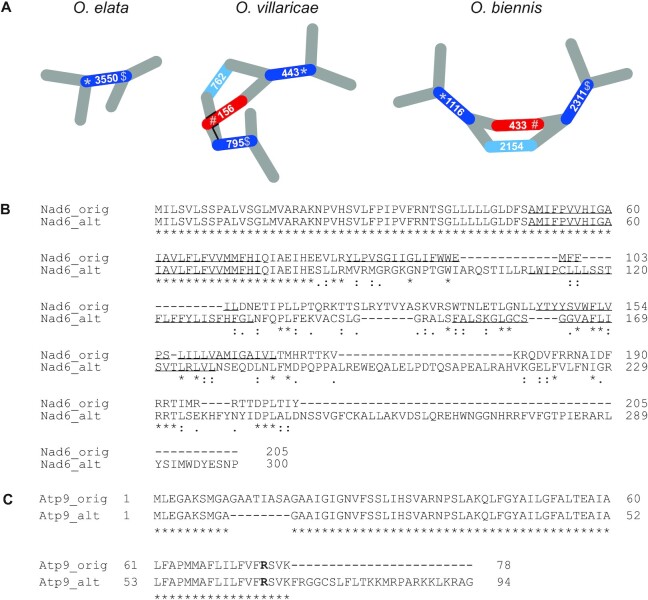
Putative cryptic CMS loci. Illustrated are two mitochondrial genes, for which, besides their fully functional version, another alternative version was observed. (**A**) Occurrence of an alternative sequence (contig) within the *nad6* gene locus, which is directly apparent from the IDBA graph itself. Visualized are the contigs making up the *nad6* gene locus within the final, curated IDBA graphs of the investigated *Oenothera* species. The fully functional *nad6* gene consists of the contigs in blue (representing the RRPs) and light blue, whereas the contig in red represents an alternative sequence, which is present in *O. villaricae* and *O. biennis*, but is absent in *O. elata*, where only the native *nad6* gene as RRP exists. (*) indicates shared start codon of both *nad6* isoforms, whereas ($) and (#) mark different stop codons of the fully functional and alternative *nad6* isoform, respectively. (**B**) Protein sequence alignment of the two Nad6 variants. Transmembrane domains as predicted by Phobius are underlined. (**C**) Protein sequence alignment for Atp9 for which, besides the original version (atp9_orig), a putative second version (atp9_alt) was identified in *O. elata*. Bold-face amino acids highlight the RNA editing site, leading to a truncation of both versions.

Beyond mitochondrial genome reconstruction, our ISEIS algorithm unveiled a potential locus for cryptic cytoplasmic male sterility (CMS). An alternative *nad6* gene is generated that is present in the IDBA graphs of two of the three *Oenothera* species (Figure 8A). The alternative sequence is present in *O. villaricae* (berS_156) and *O. biennis* (suavG_433) but is lost in *O. elata*. In comparison to the native Nad6 protein (Figure 8B), the recombinant versions share their N-terminus (80 amino acid residues), whereas further downstream, the protein sequences differ. Here, the stop codon lies in the alternative contig sequence instead of the next contig with identical sequence. The relatively extensive differences between the recombined version and the the non-recombined protein may result in differences in structure and function of the Nad-complex (Figure 8B).

Besides this locus, which is directly discernible from the IDBA graph, another locus was identified as a duplication of *atp9* gene in all three investigated *Oenothera* species. With its original version, the alternative one shares the same start codon. The translated alternative protein sequence, in turn, displays a deletion of eight amino acid within the N-terminal sequence and should be 16 amino acid residues longer than the original *atp9* gene. However, the 16-amino acid extension is seemingly not translated, because, as is the original gene, also the alternative *atp9* gene becomes edited at *atp9*eU199R*. This leads to a premature stop codon after amino acid 66 (Figure 8C). If the editing factor of the premature stop codon acts as fertility restorer locus is an interesting hypothesis, which deserves future investigation.

Lastly, 40 bp upstream of the *atp9* variant, a 789 bp long ORF is present. The amino acid sequence of this ORF has a high similarity to ORF873 in *Helianthus annuus*, where it is associated to the MAX1 type of cytoplasmic male-sterility in sunflower.

## DISCUSSION

Starting with the publication of the *Arabidopsis thaliana* mitochondrial genome in the year 1994 (78,79), and after more than a quarter-century of plant mitochondrial genome (PMG) research, there is still an obvious imbalance of published land plant organelle genomes. As of March 2022, the NCBI holds 24 times more chloroplastidial than mitochondrial genomes of land plants (7708 chloroplastidial versus 323 mitochondrial ones). This can be explained by a research focus on chloroplast genomes, especially for phylogenetic studies, but also by technical obstacles associated with sequencing mitochondrial genomes. Genome Skimming is the most popular approach to sequence plastid genomes (80–82) as even whole genome sequencing at low coverage contains enough reads to assemble them. Many popular reference-based or *de novo* assemblers can be used afterwards to generate the desired chloroplast genome as a single contig, as they are in almost all cases structurally conserved and repeat-poor (83–85). However, such a straightforward workflow is not applicable for PMGs, as they exhibit a high degree of complexity. Our success, reported in this study, relied on an adapted and optimized workflow and the developed methodology. By approaching the post-assembly task from a graph-based perspective, we were not only successful in constructing a structural model that captures the complexity of PMGs but also in predicting a defined spectrum of alternative PMG variants.

As already argued above (see paragraph ‘Mitochondrial genome reconstruction and genome comparison’), we followed the rationale that searching for the possibility to create a ‘master circle’, a circularly closed path through the graph that traverses all sequence segments, is worthwhile, despite disputes over the actual existence of such master circles (3–7). (i) Our approach is data-driven. If such a path is possible based on sequence data, it may possibly exist. (ii) Such a master circle is useful construct, as it captures the whole of mitochondrial genomic contents. While this study cannot present conclusive evidence for the existence of master circle genomes, our sequencing data at least allow them, in principle. Furthermore, in part, our data corroborate the existence of an ‘un-rolled’ path through the network. If the master circle path requires a repeated traversal of a particular segment, correspondingly increased read coverage was observed (Supplementary Table S4).

Our initial test of four *de novo* assemblers revealed that only one (IDBA) was capable to generate a closed circular graph, despite using NGS libraries of mainly mitochondrial origin and a purity level of mtDNA with up to 95%. The IDBA assembler has been designed for sequencing data with highly uneven sequencing depth and is therefore capable of distinguishing between nuclear, plastidial and mitochondrial sequences as these are very unevenly distributed in mitochondria-enriched samples. Of note, IDBA is frequently used in metagenomic sequencing projects (86–91). As mitochondrial genomes can be regarded populations of polymorphic genomes and furthermore may be mixed in sequence-read sets with the other cellular genomes (plastidial and nuclear), the suitability of IDBA in our setting may reflect its design scope. The strength of IDBA is supported by the observation that almost the complete plastid genome exists within the IDBA assembly. Newbler assemblies most likely failed to construct a circular graph, because the coverage of the Roche 454 data with approximately 35× and 74× was possibly too low, i.e. causing the Newbler algorithm to misassemble and/or introduce scaffolding errors. MIRA assemblies have failed to create circular graphs as well, which is surprising, as it is explicitly mentioned in the manual that it can handle repetitive sequences. Given its exorbitant runtime for ultra-deep coverage data sets, MIRA should rather be avoided for such data. It also turned out that only IDBA was able to split assemblies into structural units of repetitive and non-repetitive sequences, which was essential for our post-assembly methodology and is explainable by the implemented De Bruijn graph algorithm (46).

There are two limitations of IDBA that were uncovered in our graph-based visualization and necessitated manual curation of assemblies and/or graphs. One is related to assembler accuracy, reflected by the occurrence of unconnected vertices. This can be biologically relevant as, on the one hand, they can represent linear variants of PMGs, but on the other hand can also be explainable by false-positive contig breaks/ends. The latter seems to have been the case for *O. biennis* and *O. elata*, as in all instances, a high number of read pairs were identified within the sequencing data used for the assembly, which went beyond the contig termini. The second limitation can be traced back to technical and biological noise within the sequencing data itself. The technical noise is explainable by the impurity of the fraction taken from the Percoll-sucrose gradient, which was especially observed in the *O. elata* mtDNA dataset. Biological noise can be attributed to so-called promiscuous DNA resulting from inter-compartmental DNA exchange. DNA transfer from the plastid into the nucleus (NUPT) and mitochondria (MIPT) occurred often during plant evolution (92–96), and in some cases, even from the nucleus to the mitochondrion, as also described very early within the evening primrose (97,98). This contamination resulted in the interconnection of plastidial and mitochondrial subgraphs, introgression of single contigs within the mitochondrial graph, and/or false-positive sequence overlaps in regards of true mitochondrial origin. Hence, it made it necessary to remove sets of vertices and edges (subgraphs), pairs of vertices (contigs) or single edges (sequence overlaps) respectively. As the underlying reasons from which these problems arise are manifold, their corrections can only be done manually or in some cases semi-automatically by the user, as it is challenging to translate human experience and intuition into computational algorithms.

Obviously, promiscuous DNA does interfere with assembly strategies that start from whole genome sequencing data generated from total DNA. An obvious solution to identify mtDNA reads by unmapped reads, i.e. mapping total sequencing data first against available plastid and nuclear genomes of the species of interest, should be avoided. This would inevitably result in unconnected contigs, as sequences, which are present in both organellar genomes would be removed beforehand and could be falsely be interpreted as biologically relevant. Available reference-based, such as mitoBIM (99) and NOVOPlasty (100), as well as k-mer-based assemblers, such as Norgal (101) assert to be able to assemble mitochondrial genomes. The wrapper program mitoBIM uses MIRA to reconstruct mitochondrial genomes (99). It employs an iterative two-tiered approach, by first baiting perceived mitochondrial reads from total DNA sequence with known mitochondrial genes and/or genomic sequences using MIRA in mapper mode. In a second step, it assembles those reads using the MIRA in assembler mode. As, in our hands, MIRA cannot generate a circular graph for mtDNA-enriched DNA (see above), our assumption is that it will most likely run into problems for a total DNA data set as well. However, and probably more importantly, all reference-based assemblers falsely integrate nuclear-mitochondrial DNA (NUMT) reads. By definition, they do not belong to the mitochondrial genome, have higher mutation rates, and, most importantly, different recombination events (102–104). This can lead to uncorrectable/wrong graphs and introduces SNPs/indels, which do not actually belong to the PMG itself. The authors of the Norgal program explicitly mention that assembly outcomes from NGS data, in which k-mer distributions are inseparable, should be handled with caution (101). Nevertheless, a k-mer pre-filtering of the sequencing data before the assembly step can be evaluated for each project/case using KAT (105) or equivalent software.

The need for sequence assembly visualization is as old as sequence assembly itself. For example, Bandage, a tool that has been established as a standard program to investigate graph-based assembler outputs, is frequently used even for large nuclear genomes, but was also used to visualize the assembly of *Sitka spruce* to identify the PMG (14,106). In most cases, it is employed to investigate and solve problematic issues such as loops or bubbles. The Contiguity software can help in visualization of non-graph-based assembler outputs, as a reverse mapping step is implemented to find links between different contigs (15). However, this is very time-consuming as one needs to arrange the resulting linked contigs manually, which might take a long time. Also, Contiguity is not orientation/strandedness-aware, which, in the context of sequence overlaps (Supplementary Figure S1) is important to be considered. In our study, accounting for orientation did lead to the identification of real, contiguous stretches of contigs. IDBA, our assembler of choice, is a true De Bruijn graph-based assembler, but lacks any kind of supported graph-based output format. Consequently, with our SAGBAC pipeline, we implemented those missing features including semi-automatically graph output curation that reduces necessary hands-on time.

The final graphs, generated here, can be best described as circularized models of PMGs, in which the repetitive contigs are easily identifiable by the occurrence of ‘double forks’ (Figure 1C). Repeats are commonly categorized in large, intermediate-, and small-sized repeats depending on their sizes and can present in different amounts and proportions within the plant kingdom (58). All three categories are present in the three investigated *Oenothera* species. While sharing three RRPs, each of the species has its own RRPs (especially a unique long-size repeat). This is in line with previous findings in which repetitive sequences can differ between closely related species (12,107).

The qualitative validation of our model for *O. villaricae* by molecular biology techniques using PCR confirmed all contig-repeat-contig combinations for all RRPs and one RRP was verified by a Southern blot. Additionally, we employed two more advanced technologies, Illumina Mate-pair and PacBio RSII data, not only to verify the qualitative, but also quantitative status of all RRPs of *O. elata*. Our stoichiometric analysis based on these two datasets revealed that none of the five RRPs in *O. elata*, regardless of their size, show a near equal occurrence among all four CRCs (Table 3). Thus, none of the RRPs seem to recombine frequently. This is in contrast to literature reports that found a balanced occurrence at large repeats in various plant species (e.g. (10,78,79,108,109)) while unbalanced occurrences have been reported to be less frequent (110). By contrast, our data suggest that for *Oenothera*, not only for intermediate-size repeats, but also for large-size repeats, unbalanced variant frequencies were observed, which is an indicator for presence of a dominating genomic conformation (considering johSt_3550 to be an LSR, which is, to some degree, an arbitrary, albeit k-means-based call). Future investigations in other *Oenothera* species are needed to resolve that apparent discrepancy.

Many genomic studies used paired-end sequencing data to assess stoichiometry of various repeat conformations (e.g. (108,110–112)). Of note, while our approach necessitates the removal of false-positive edges (clean up), in many studies, read mapping information is used to scaffold contigs further, or in our terminology, to restore (‘false-negative’) edges to obtain a master circle (in terms of CRCs: 2 CRCs for each RRP, see also Figure 1) or, if one master circle cannot be found, two or more autonomous circles were reported (8,113,114). But as, for the sake of generating a submission-ready assembly, they generally focused less on underrepresented configurations (the other two CRCs for each RRP), and thus, ‘double forks’ needed to be excluded. However, as long as the real abundance of all types of variants cannot be determined quantitatively (each mtDNA molecule as one DNA sequence), all topological variants should be kept, as accomplished implicitly by considering genome assemblies as graphs.

The feasibility of stoichiometric repeat analyses depends directly on read length and/or library insert size of generated NGS data. Both ends of long reads need to be anchored or both mates of read pairs need to be mapped entirely to the unique flanking sequences of repeats to be analyzed. While paired-end library insert size is limited to up to 600 bp, PacBio and mate-pair libraries (sometimes falsely named paired-end) can span up to several dozens of kilobases. But there are good reasons for not using PacBio data directly for initial assemblies. Available assembly programs used for PacBio data, such as Falcon or Canu (115,116), are not designed to work with the smallest structural components of an assembly. Consequently, those assemblers could not resolve complex nested repeats and are incapable to generate all distinct variants, which may exist in PMGs and will report only the most frequent variant. The same is true for incorporating mate-pair data within *de novo* assembly or scaffold processes itself.

Stoichiometric analyses offer valuable clues with regard to recombination frequencies and variant stoichiometries. While a frequent recombination at RRPs is strongly connected to a balanced variant stoichiometry (10,117), an infrequent recombination at RRPs should be associated with appreciable differences in variant stoichiometry. Our observations suggest that RRPs in *O. elata* appear in quantitatively different amounts (usage factors with approximately 2× and 3×, see Table 3) consistent with a low recombination rate. In 2020, it was reported that knockout mutants for *msh1*, *recA3* and *polIb* in *Arabidopsis thaliana* showed distinct patterns of large shifts in abundance of the mitochondrial genome (2). All three genes are involved in cytoplasmic DNA replication, recombination and repair. As msh1 is also involved in controlling recombination activity in mitochondria (118), it appears plausible that the stoichiometry is directly or indirectly influenced by this gene, too. This hypothesis is further corroborated by several other studies, which reported changing amounts of mitochondrial genome variants (107) and tight temporal control of stoichiometry (119) during plant development as well as changing copy number of mitochondrial genes that differ between various genes and tissues (120).

As introduced in Figure 1, with the concept of RRPs embedded in ‘double forks’ and CRC combinations, all master- and sub-circles can be inferred from our graph-based model. The urgent need for such a model was adamantly recommended by Kozik *et al.* (10), as >50% of the PMGs between the years 2016 and 2019 were presented and published just as circles without even mentioning and considering that other models or variants are possible. This resistance to surrendering the one-master-molecule view is surprising, as experiments often failed to recover large circular molecules and studies postulated and provided evidence for the existence of alternative models, including linear configurations (3–6,), various independent mtDNA molecules (121–123), branched structures, circularly permuted linear fragments or even more complex structures (11,12). Yet, despite the inadequate reflection in databases, it is now commonly accepted that alternative forms of PMGs most likely co-exist within each cell. With this study we proposed a coherent, graph-based framework that offers a unified view for the complexity of PMGs.

Of note, our results were obtained using eight weeks old mature leaves, a choice driven primarily by the needs to obtain sufficient mtDNA quantities. As it has been reported that leaf maturation and exposure to light and stress conditions leads to degradation of organellar genomes (124,125), the possibility of a presence of such degradation products as part of our structural variants needs to be borne in mind. While the leaf material used in this study was still relatively young and not senescent, it remains to be studied, how populations of different mtDNA topologies evolve during aging and stress exposure. With this study, we believe to have introduced a framework for capturing and describing topological variants. In summary, the current state of plant mitochondrial genome research supports the view that mitochondria are populations consisting of different amounts of various variants at different developmental stages and tissues.

To generate PMG sequences for database submission, rules for traversing the obtained graphs were needed, as only individual linear or circular individual molecules are accepted, but not graphs with different traversal paths. To accomplish this, it was necessary to break our traversal rules (see above) as repeats can occur more than twice, as also shown before (118). Also large repeats, as identified in *O. elata* harboring *rRNAs* and *nad5* exons, had to be tolerated, which, however, is supported by their existence in other plant species within the asterid clade: *Rhazya stricta* (126) (repeat size 36.3 kb, NC_024293.1) and Daucus carota (127) (repeat size 14.2 kb, NC_017855.1). But so far, *Oenothera* is the only genus from the rosid clade, in which this kind of repeat is present or absent within a single genus.

The performed homology-based gene search revealed the presence of all essential gene families (11) and tRNA sets of mitochondrial and plastidial origin (128) found in a wide range of PMGs. But it should be emphasized that our study is one of the few (129), in which homology-based annotation was completely supported by a set of previously published genes for *O. villaricae*, but, more importantly, by an RNA-seq based gene expression atlas for *O. elata*. By a subsequent SNP analysis, we were, moreover, able to transfer the concept of RNA editing investigated in *Oenothera* (130) to a mitochondrial-wide RNA editome level, which, with 681 RNA editing sites, is as large as expected for a genus of the angiosperms clade (131).

Three unexpected findings are particularly noteworthy. Two pertain to potential loci for cryptic CMS (132–134), as they encode for alternative variants of Nad6 and Atp9 proteins. And, surprisingly, in both cases, they do not originate from a fusion event between the original gene and a downstream ORF, which is the common definition of a cryptic CMS. Only the mid-part of the *nad6* gene is exchanged causing structural and functional differences, possibly hindering the electron transport itself. So far, CMS candidates, in which *nad6* participate, have only been identified in *Mimulis guttatus* (110,135). CMS loci involving the *atp9* gene were reported many times in the literature, first in Petunia (136,137). But also here, our reported CMS locus differs from the standard CMS definition by involving an RNA editing event to neutralize it, which was also reported previously to occur (138). Regarding the third finding, we wish to highlight that some genes are extending into some of the RRPs or the RRPs are, as a whole, part of them (Table 2). As three of those six genes represent the top-3 in a ranked-by-frequency list of CMS loci in major crops (134), these RRPs are perhaps involved in the creation of cryptic CMS loci. Some mitochondria-associated phenotypes (20,139,140) were observed in crosses between phylogenetically distant *Oenothera* species, which might lead to an incompatibility of RRP sets and nuclear regulators. Such incompatibilities potentially may have played an important role in the evolution of *Oenothera* species and may act as speciation barriers.

## CONCLUSION

With the newly developed SAGBAC pipeline and its ISEIS core algorithm, it is now possible to systematically investigate the overall mitochondrial status in different *Oenothera* species as well as in various organs and developmental stages and to understand mechanistically the influence of RRPs on the creation of novel, biologically relevant/active open reading frames (cryptic CMS loci), which might explain some mitochondria-associated phenotypes observed within the *Oenothera* genus. In addition, our methodology will possibly allow to identify different mitochondrion types and integrate them as a third dimension into the nuclear-plastome-compatibility chart (141), which was created in the past, linking them to the evolutionary context of speciation.

## DATA AVAILABILITY

All NGS-related (except PacBio) data are submitted to the Short Read Archive under BioProject PRJNA757974 (see Supplementary Table S2 for details of SRR identifier assignment). Final mitochondrial genomes are available at the NCBI Nucleotide database under the following accessions: *O. villaricae* (MZ934755), *O. biennis* (MZ934756), *O. elata ssp. hookeri* (MZ934757). Raw assembly outputs are downloadable on figshare (doi:10.6084/m9.figshare.16496814) and SAGBAC scripts are accessible through github (https://www.github.com/AxelMacFoly/SAGBAC).

## Supplementary Material

lqac027_Supplemental_Files
