# Supplementary material for: Graph-based models of the Oenothera mitochondrial genome capture the enormous complexity of higher plant mitochondrial DNA organization
Source: NAR Genom Bioinform. 2022 Mar 31;4(2):lqac027. doi: 10.1093/nargab/lqac027 (PMC8969700; doi:10.1093/nargab/lqac027)
Supplement: lqac027_Supplemental_Files [file lqac027_supplemental_files.zip › Supplementary_Figures_Tables_noColoring.docx]

**Supplementary Figures**


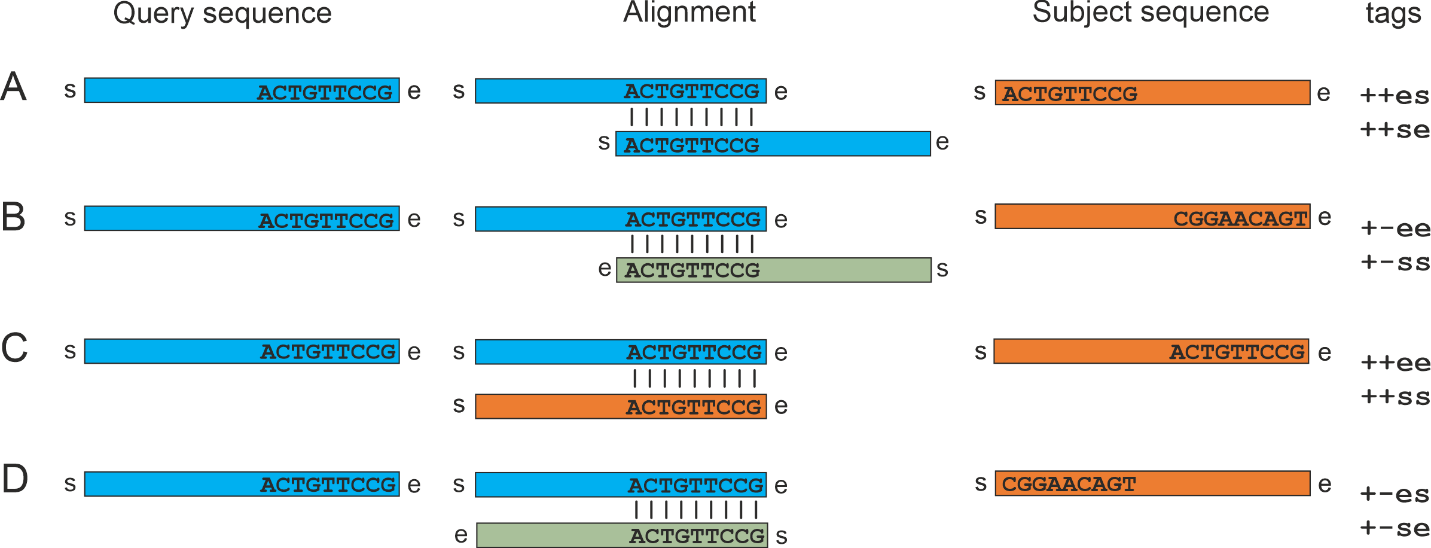


**Supplementary Figure 1.** Orientation/strand combination rules of sequence alignments allowing for contig connectivity.

Illustrated are four different sequence alignments, where query sequence and subject sequence have an identical sequence at one of their ends. (A) and (B) show a sequence alignment creating a stair-like situation. In the context of creating a complete mitochondrial genome, these two possibilities make sense in order to get a contiguous sequence, whereas in (C) and (D) both sequences look in the same direction. With that it is not possible to construct a contiguous concatenated sequence. Start (s) and end (e) of sequences is set on the basis of orientation within the original *de novo* assembly fasta files. Blue rectangles: query sequences; Orange rectangles: Unchanged subject sequences; Green rectangles: Reverse complement of subject sequence. Tags: possible combinations of orientation and strand for each sequence alignment type (A)-(D).


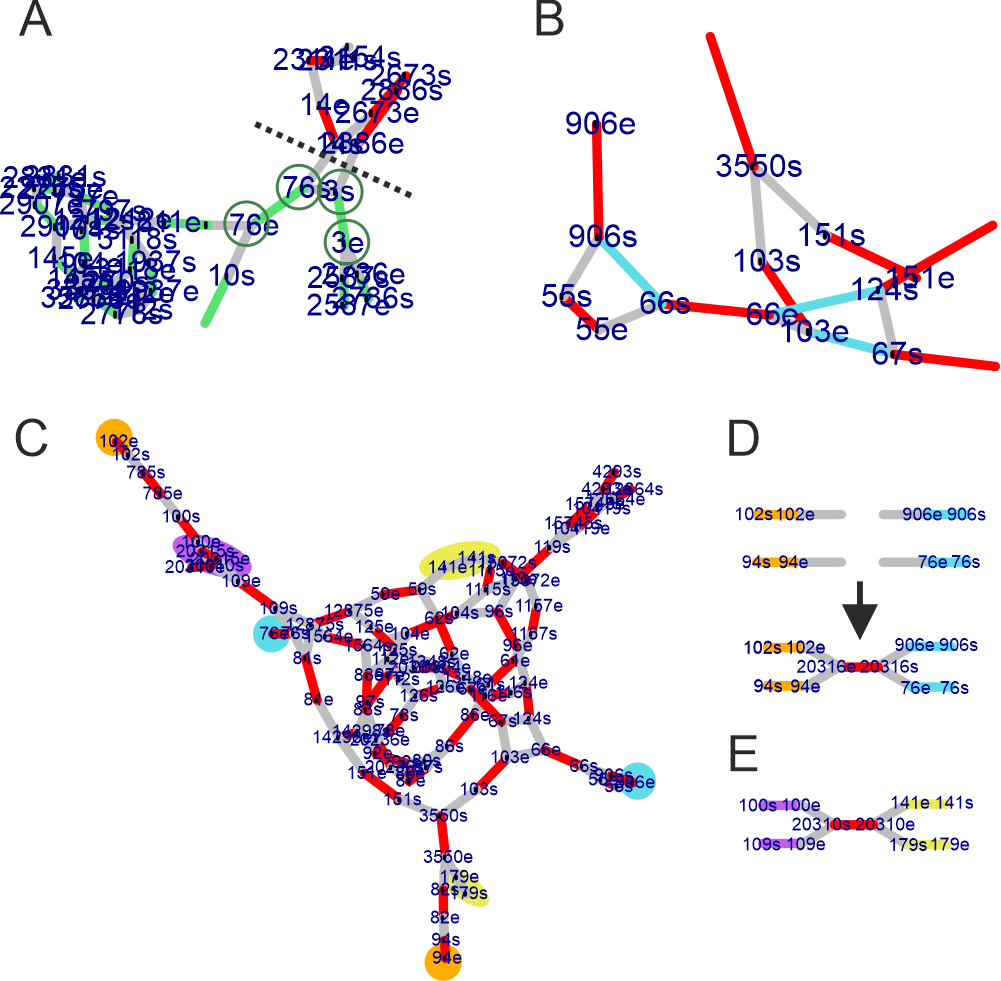


**Supplementary Figure 2.** Exemplarily illustrations of manual graph curation steps.

Within a graph, each contig is defined by two vertices (s=start, e=end), which are linked by a colored edge within the graph. An overlapping event between two different contig ends is represented by a grey edge. (A) Remove plastidial subgraph (Part of uncurated *O. biennis* graph): By identifying plastidial contigs (vertices with green edges), which are connected to the mitochondrial subgraph (vertices with red edges), a removal of vertices for contig johSt_76 and johSt_3 (parameter “76,3” within the R script) leads to inaccessibility of the remainder of the plastidial subgraph by the ISEIS algorithm. (B) Correction of falsely connected contigs (part of 4th graph of *O. biennis*): Contigs that were identified as falsely connected can be easily removed with a parameter (“906s_66s,103e_67s,66e_124s” within the R Script), which will remove the three cyan edges and reduce the connectivity of the whole graph. (C-E) Curate misassemblies: (C) *O. elata* graph after plastidial graph removal, showing some mis-/unconnected contig ends’ (colored vertices). (D) Extending unlinked contig end sequences (orange johSt_102, johSt_94 and cyan johSt_906 and johSt_76) by searching for overlapping sequences within Illumina raw read data leads to the creation of a new contig (johSt_20316), and with it, a newly formed double fork. (E) Blast output investigation reveals too short a blast hit at a contig end (johSt_179) and a blast hit not at the direct end of a contig (johSt_141), but almost at the end. Sequence extension and break of contig leads to overlaps with unlinked vertices of contig johSt_20310, also recovering here a double fork.


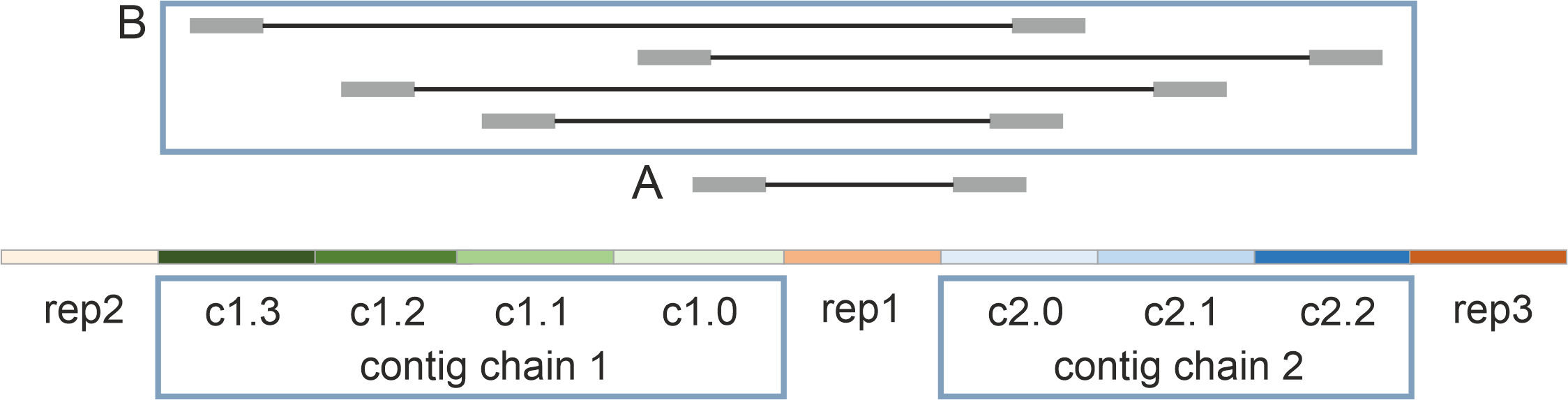


**Supplementary Figure 3.** Definition of contig chains.

As contig sizes within the IDBA graph can vary between hundreds of bases and many dozens of kilobases, mate pair fragments, because of their large insert size of 5 kb, can span more than one contig. To overcome this issue, so-called contig chains were defined as shown in the following example: (A) Repetitive sequence rep1 is flanked by the contigs c1.0 and c2.0. One mate of the pair maps to c1.0 whereas the other mate maps to c2.0 (in short c1.0-c2.0). (B) But in any other read pair, i.e. c1.1-c2.0, c1.2-2.1, c1.0-c2.2 and c1.3-c2.0 one or both mates map to contigs not connected directly to the repetitive sequence rep1, but are spanning it. To include those mate pairs into the analysis, all contigs between two repetitive sequences were grouped to one contig chain. In this example, contig chain 1 harbors the contigs c1.0, c1.1, c1.2 and c1.3, whereas contig chain 2 consists of c2.0, c2.1 and c2.2.


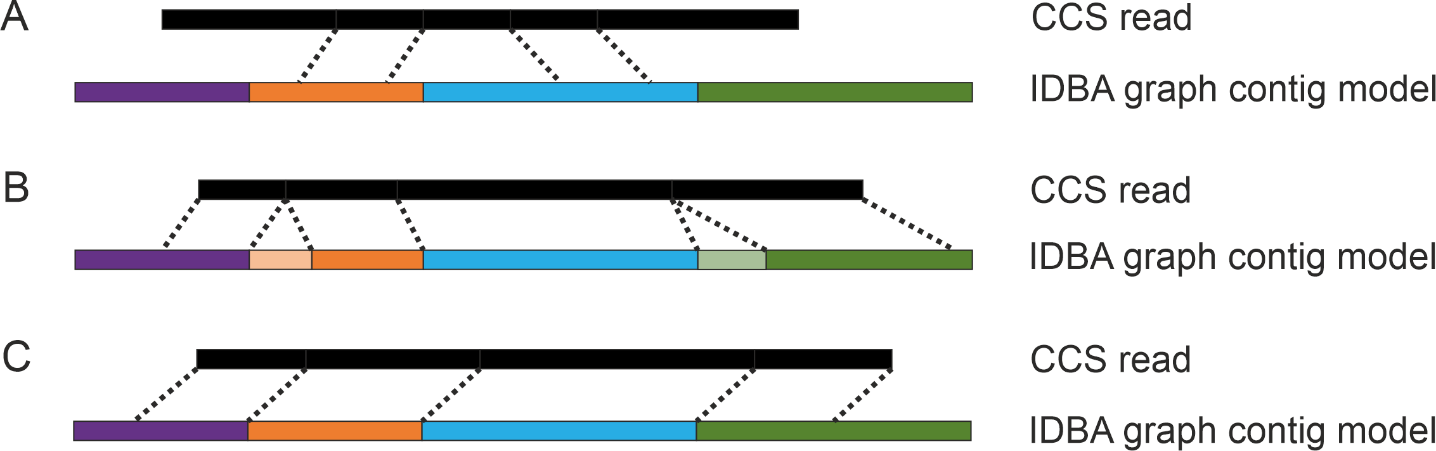


**Supplementary Figure 4.** Filtering strategy to identify nuclear and mitochondrial reads in the PacBio circular consensus sequences (CCS) reads.

Organellar origin of the CCS reads was determined by the completeness and order of their alignment to the IDBA graph: (a) only parts of a CCS read can be aligned to one or more IDBA graph contigs (dotted lines). (b) the complete CCS read can be aligned to IDBA graph contigs, but does not follow the IDBA graph contig model. Both, (a) and (b), originate very likely from mtDNA translocations to the nucleus, since the remaining sequence of the CCS reads and/or its structure is unknown from the perspective of the IDBA graph. (c), The IDBA graph contig model can be aligned completely of the CCS reads. These reads originate very likely from the mitochondrion as they are following the IDBA graph model. (a) and (b) are filtered out whereas c remains for the stoichiometric analysis.


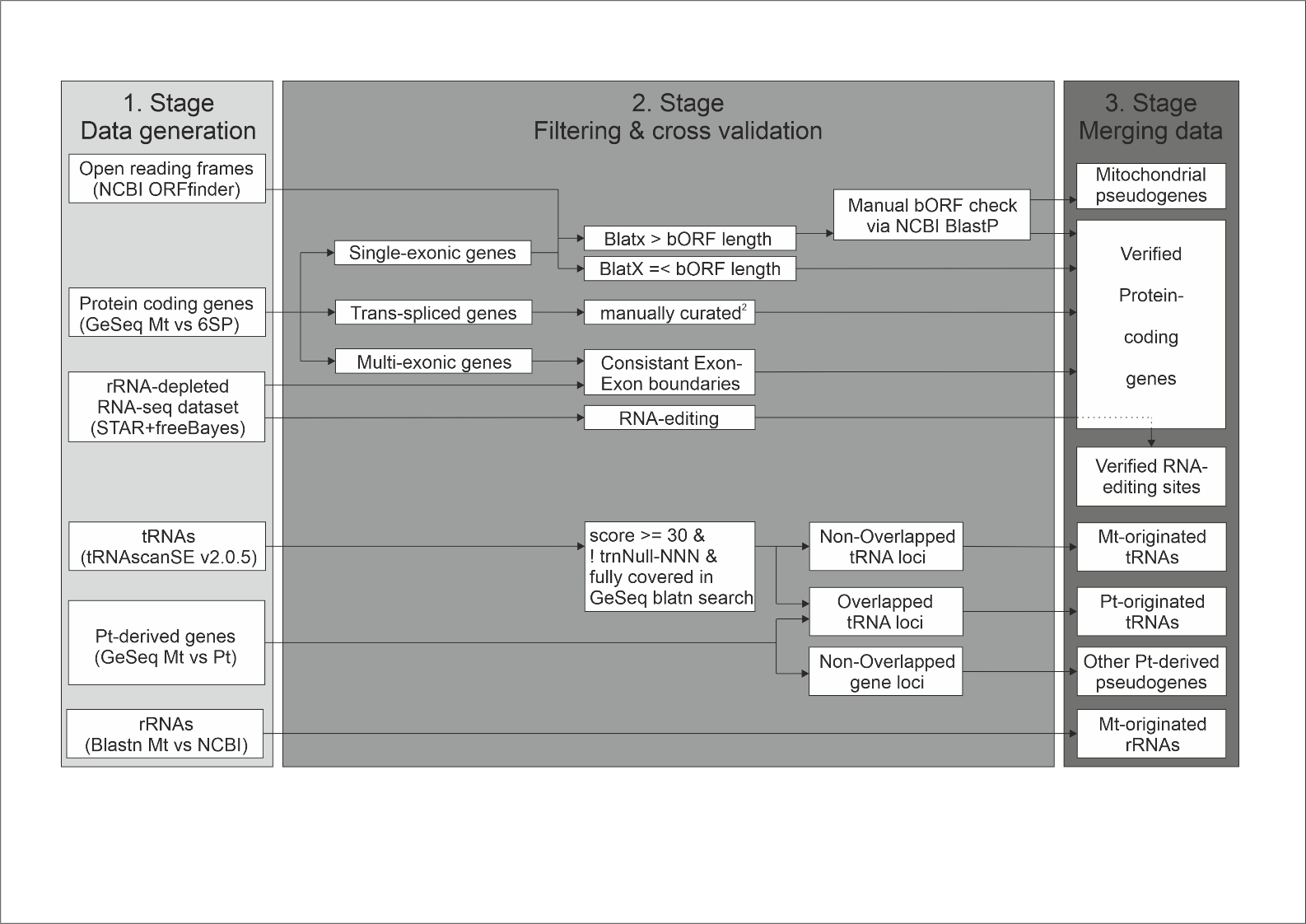


**Supplementary Figure 5.** Annotation workflow schema.

Illustrated is the annotation pipeline, which is partitioned into three stages: (1) Data generation, (2) Data filtering and cross validation between the datasets and (3) Merging of all datasets to generate a genbank ready for submission. For details, see Material and Methods.


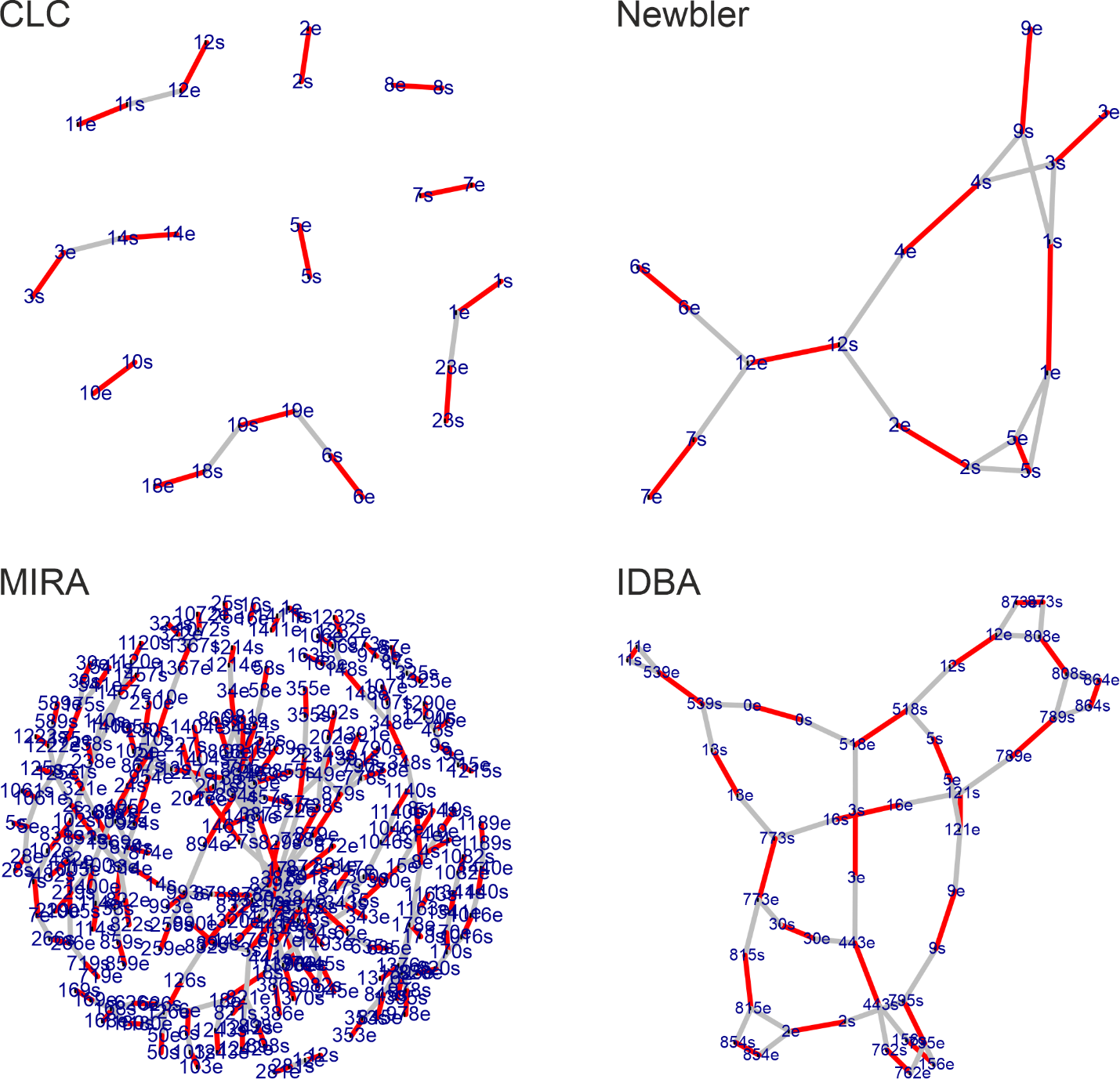


**Supplementary Figure 6.** Comparison of raw graphs for *O. villaricae* from four different *de novo* assemblers.

Exemplary uncurated contig datasets of four different *de novo* assemblers, namely Newbler, MIRA, CLC, and IDBA, were used to generate an undirected graph using our ISEIS pipeline. Within a graph, each contig is defined by two vertices (s=start, e=end), which are linked by a red edge within the graph. An overlapping event between two different contig ends is represented by a grey edge.


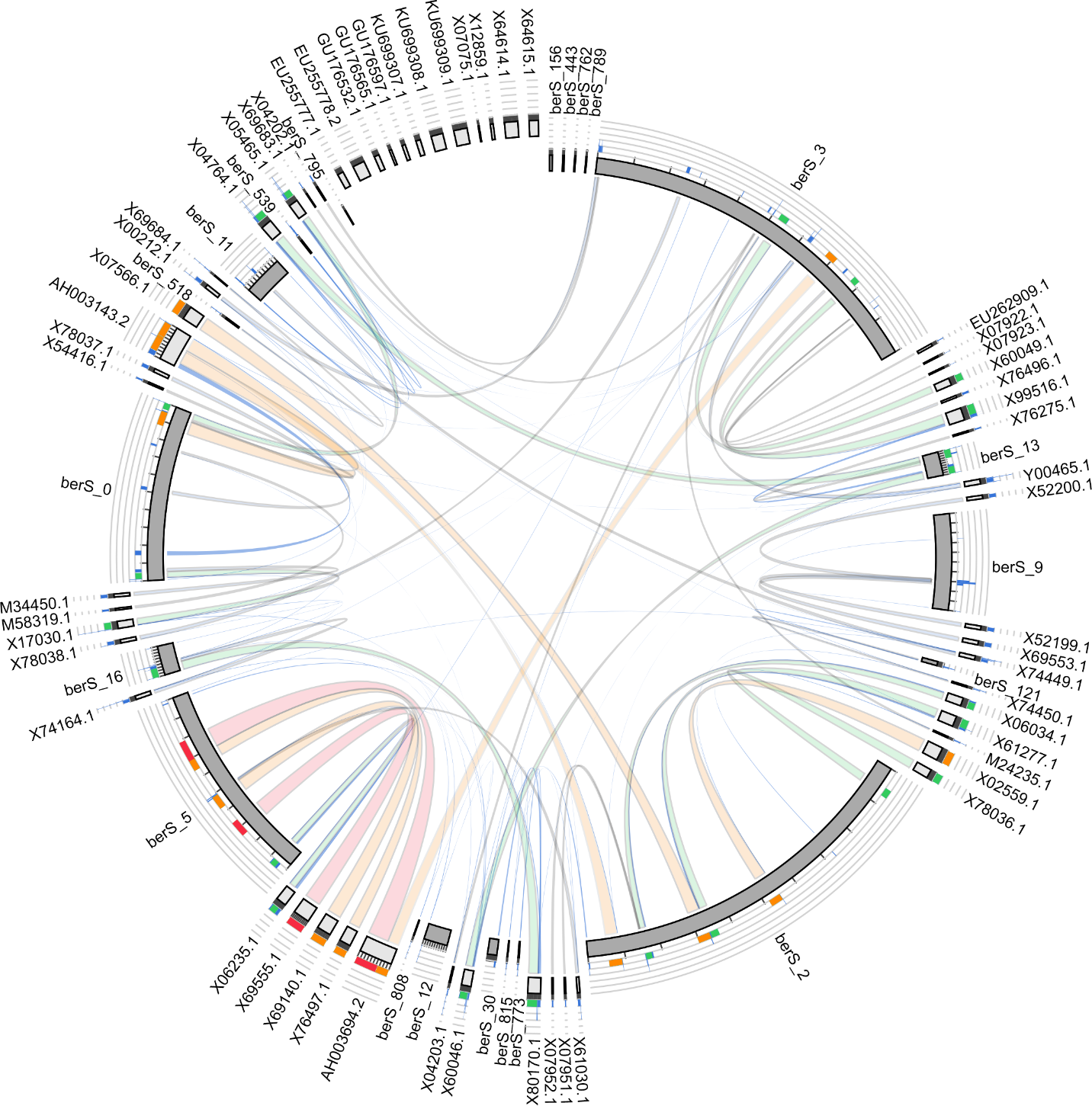


**Supplementary Figure 7.** Circos-based visualization of the BLAST result between the NCBI sequences and the contigs that are part of the final mitochondrial sequence of *O. villaricae*.

Shown is an untangled Circos plot (with the untangled option) generated by Circoletto for the BLAST search between the IDBA graph contig set of *O. villaricae* and sequences from NCBI harbouring the taxonomic ids of *O. villaricae* (3941 and 3950). The quality of the BLAST hits is represented by the link color from red (best bit scores), over orange and green to blue (worst bit score).


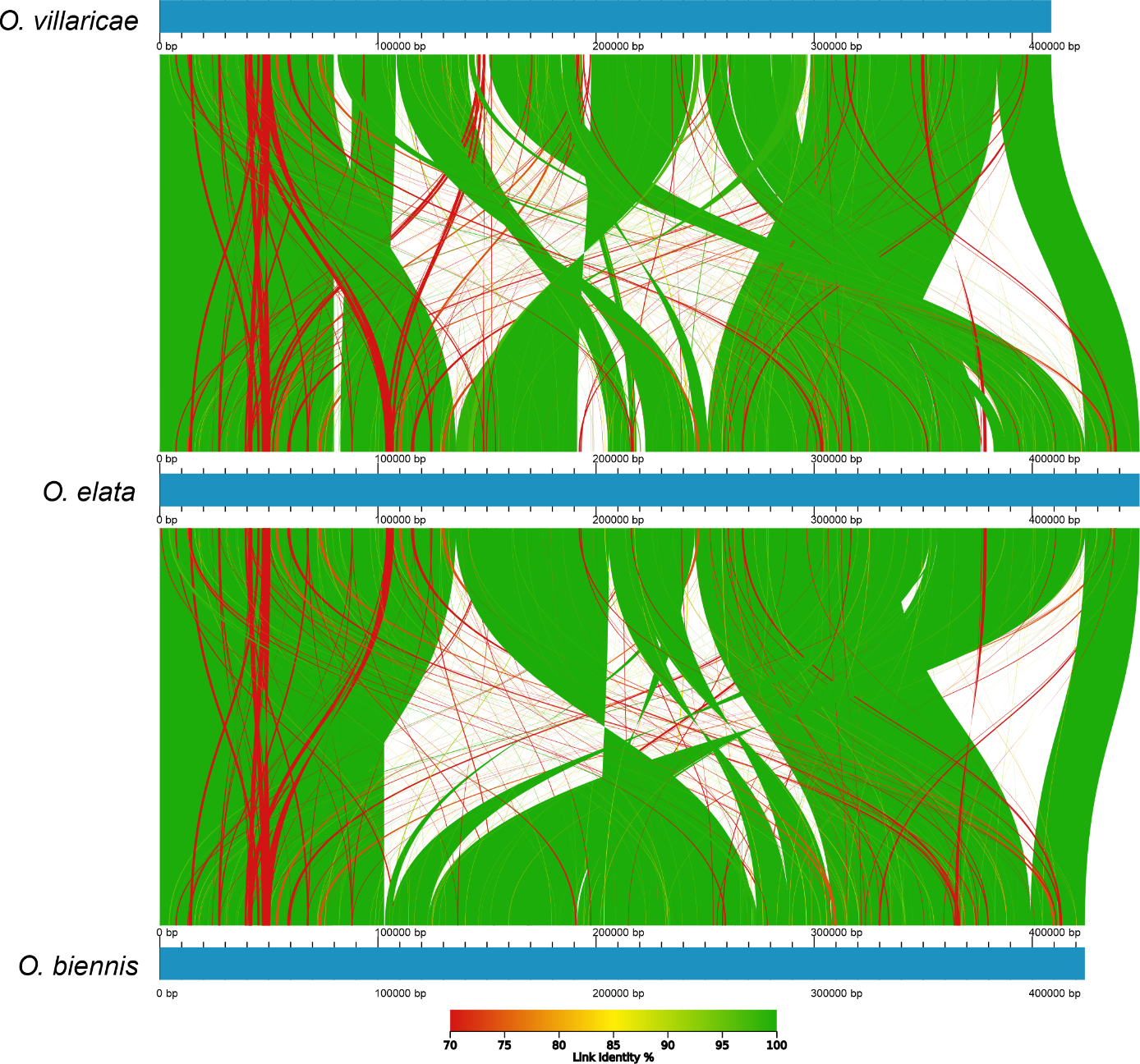


**Supplementary Figure 8.** Genome alignment.

Visualized is a sequence alignment of all three investigated mitochondrial genomes using AliTV with default parameters. Link coloring refers to the percentage of identity ranging from 70% (red) to 100% (green).

**Supplementary Table 1.** List of primers for PCR amplification and southern blot probe synthesis.

| **Name** | **Forward primer (5' to 3')** | **Reverse primer (5' to 3')** | **Length (nt)** |
| --- | --- | --- | --- |
| pr_9e_out/pr_121e_out | TGAGCCAGGATCGAACTCTC | GGGAATACGGATTCGAACCACTGACAC | 505 |
| pr_9e_out/pr_789e_out | TGAGCCAGGATCGAACTCTC | CTGGCTACGGAATGAATCAAAGAACTG | 1487 |
| pr_5e_out/pr_789e_out | GTTGCCCTTCTGAGGCTAACCCGTC | CTGGCTACGGAATGAATCAAAGAACTG | 1486 |
| pr_9e_out/pr_16e_out | TGAGCCAGGATCGAACTCTC | GACTTGAATGGGCTGCCTACGTATCTC | 1613 |
| pr_5e_out/pr_16e_out | GTTGCCCTTCTGAGGCTAACCCGTC | GACTTGAATGGGCTGCCTACGTATCTC | 1621 |
| pr_121s_out2/pr_16e_out | GACCTTCGACCTTCCCACAAGTC | GACTTGAATGGGCTGCCTACGTATCTC | 421 |
| pr_789e_out/pr_121s_out | CTGGCTACGGAATGAATCAAAGAACTG | GCTTCAGACTCTCCGCTCTCAGAC | 244 |
| pr_121e_out2/pr_5e_out | AGCAAAACCATCGCTCCGGCAC | GTTGCCCTTCTGAGGCTAACCCGTC | 379 |
| pr_30e_out/pr_443e_out | GATCGGCGTAGTCGCTCATAGGGAC | CAGAGACCAAAGCAGGGCTCGAC | 669 |
| pr_3e_out/pr_762s_out | CATCACATGCCTGCAGATGTAGTCAC | TACCACTCACAGGTAAATAGCGCAAGAC | 655 |
| pr_443s_out/pr_762s_out | TCCCATCCCAGTCTTTCGCAACAC | TACCACTCACAGGTAAATAGCGCAAGAC | 188 |
| pr_30e_out/pr_762s_out | GATCGGCGTAGTCGCTCATAGGGAC | TACCACTCACAGGTAAATAGCGCAAGAC | 903 |
| pr_3e_out/pr_156s_out | CATCACATGCCTGCAGATGTAGTCAC | CCGGAACAACCCAACCCCTTACTTAGAG | 869 |
| pr_30e_out/pr_156s_out | GATCGGCGTAGTCGCTCATAGGGAC | CCGGAACAACCCAACCCCTTACTTAGAG | 1117 |
| pr_156s_out/pr_443s_out | CCGGAACAACCCAACCCCTTACTTAGAG | TCCCATCCCAGTCTTTCGCAACAC | 405 |
| pr_443e_out/pr_3e_out | CAGAGACCAAAGCAGGGCTCGAC | CATCACATGCCTGCAGATGTAGTCAC | 421 |
| pr_3s_out/pr_12s_out | CGCTGCATCACAAGGAGCAATTGTGAG | TGTTGAGATAGGGAAGGGCAATCACTC | 551 |
| pr_0s_out/pr_12s_out | GTTCGATTCCATCCCAGTTGCCTCTC | TGTTGAGATAGGGAAGGGCAATCACTC | 797 |
| pr_518s_out/pr_12s_out | GACAGACCTCTGAACGCGAGATCACAG | TGTTGAGATAGGGAAGGGCAATCACTC | 226 |
| pr_3s_out/pr_5s_out | CGCTGCATCACAAGGAGCAATTGTGAG | CTGATGAGGCAGCAGACGGTTACCAG | 591 |
| pr_0s_out/pr_5s_out | GTTCGATTCCATCCCAGTTGCCTCTC | CTGATGAGGCAGCAGACGGTTACCAG | 837 |
| pr_0s_out/pr_518e_out | GTTCGATTCCATCCCAGTTGCCTCTC | GCCTTTCTTGGTTGGACTAAGCCCG | 465 |
| pr_5s_out/pr_518s_out | CTGATGAGGCAGCAGACGGTTACCAG | GACAGACCTCTGAACGCGAGATCACAG | 266 |
| pr_518e_out/pr_3s_out | GCCTTTCTTGGTTGGACTAAGCCCG | CGCTGCATCACAAGGAGCAATTGTGAG | 219 |
| pr_11s_out2/pr_539e_out2 | CAGTACCGAACTCTAAGCGAGTGGCTC | CACATCTTTGCATCTTGCTAGCGAGTCC | 619 |
| pr_11e_out/pr_13s_out | TGACCCTAGAGGGAATGAACGCATTCAG | GATGTTCTTACCCCGGTGTGAAAGCAG | 639 |
| pr_11s_out/pr_13s_out | CTGAGGCTCTATCTACTGCGTCTGCCT | GATGTTCTTACCCCGGTGTGAAAGCAG | 801 |
| pr_539s_out/pr_13s_out | CTCGACCTATCCCACCCTCTCGAC | GATGTTCTTACCCCGGTGTGAAAGCAG | 451 |
| pr_0e_out/pr_539s_out | GTGCCCATCACTCCAGCAATGG | CTCGACCTATCCCACCCTCTCGAC | 379 |
| pr_539e_out/pr_11e_out | GTCGAGAGGGTGGGATAGGTCGAG | TGACCCTAGAGGGAATGAACGCATTCAG | 212 |
| pr_30s_out/pr_16s_out | GGATGGAACAAGGTGCGTCTGGTG | GAAGAATTCCTACCAGCAGACTTCCCTG | 384 |
| pr_815s_out/pr_16s_out | GGACGAAGCGTAGAGTGATCTTTCCCTC | GAAGAATTCCTACCAGCAGACTTCCCTG | 364 |
| pr_815s_out/pr_773e_out | GGACGAAGCGTAGAGTGATCTTTCCCTC | CTCCTCCTCCTTCTCCTCCTTTATCGTC | 227 |
| pr_815s_out/pr_13e_out | GGACGAAGCGTAGAGTGATCTTTCCCTC | CAGGAAAATCATTAGGAGCCACAC | 397 |

**Supplementary Table 1 continued**

| **Name** | **Forward primer (5' to 3')** | **Reverse primer (5' to 3')** | **Length (nt)** |
| --- | --- | --- | --- |
| pr_773s_out/pr_13e_out3 | GACGATAAAGGAGGAGAAGGAGGAGGAG | GAGTGGCTTCGGCTGCAAGGAG | 387 |
| pr_16s_out/pr_773s_out | GAAGAATTCCTACCAGCAGACTTCCCTG | GACGATAAAGGAGGAGAAGGAGGAGGAG | 165 |
| pr_773e_out/pr_30s_out | CTCCTCCTCCTTCTCCTCCTTTATCGTC | GGATGGAACAAGGTGCGTCTGGTG | 247 |
| pr_762e_out/pr_795s_in | TACCACTCACAGGTAAATAGCGCAAGAC | TTCGTCGGAATACATCCTGTCTTTTCAC | 310 |
| pr_156e_out/pr_795s_in | CGCCGATTTGTGTTCGGGACTC | TTCGTCGGAATACATCCTGTCTTTTCAC | 292 |
| pr_795s_out/pr_9s_out | ATGCCTCCAAGGTACGAAGTTGGAC | GCTTGCATTCCCATCATCTGTGCGAG | 373 |
| pr_156e_out/pr_9s_out | CGCCGATTTGTGTTCGGGACTC | GCTTGCATTCCCATCATCTGTGCGAG | 488 |
| pr_762e_out/pr_9s_out | TACCACTCACAGGTAAATAGCGCAAGAC | GCTTGCATTCCCATCATCTGTGCGAG | 506 |
| pr_156e_out/pr_2s_out | CGCCGATTTGTGTTCGGGACTC | GACTTTGTCTATTCCCACCGGTCCG | 480 |
| pr_762e_out/pr_2s_out | TACCACTCACAGGTAAATAGCGCAAGAC | GACTTTGTCTATTCCCACCGGTCCG | 498 |
| pr_795s_out/pr_2s_out | ATGCCTCCAAGGTACGAAGTTGGAC | GACTTTGTCTATTCCCACCGGTCCG | 365 |
| pr_11e_out/pr_0e_out | TGACCCTAGAGGGAATGAACGCATTCAG | GTGCCCATCACTCCAGCAATGG | 567 |
| pr_11s_out/pr_0e_out | CTGAGGCTCTATCTACTGCGTCTGCCT | GTGCCCATCACTCCAGCAATGG | 729 |
| probe_0 | GAGCCCGGAGCGGAAAGCG | GAGGCGGCCAAGAGAGGCAACTG | 317 |
| probe_3 | CGACCAGGTACAGCATCACATTTGACAC | GGATCTTGGTTCGCGCTTTATGGCAC | 448 |
| probe_5 | CTGGTAACCGTCTGCTGCCTCATCAG | GTGCCTGAAGTGGAACTCAGCGAGT | 426 |
| probe_12 | AGCTACCCTGCTTTAGCTCACCACTC | GACATGAGACATCGAGCGCTGTAACT | 349 |
| probe_518 | GTTGGAGTAGGCGAGGGCTCTACTTG | TAGGTCGAGATTCCGTTTGCGCG | 346 |

**Supplementary Table 2.** Overview of sequenced samples and their corresponding NGS technologies, library and nucleotide origin

| **Strain** | **Nucleotide** | **Sequencing** | **Sequencer** | **Library** | **read length** | **Sequencing** | **SRR** |
| --- | --- | --- | --- | --- | --- | --- | --- |
|  | **origin** | **technique** |  | **type** | **[bp]** | **provider** | **accession** |
| *O. villaricae* | mtDNA | 454 | GS FLX Titanium | Single-end | 100-644 | EMO^2^ | SRR15620607 |
|  | mtDNA | Illumina | HiSeq 2000 | Paired-end | 101 | EMO^2^ | SRR15620606 |
| *O. biennis* | mtDNA | 454 | GS FLX Titanium | Single-end | 100-756 | EMO^2^ | SRR15620605 |
|  | mtDNA | Illumina | HiSeq 2000 | Paired-end | 101 | EMO^2^ | SRR15620604 |
| *O. elata* | mtDNA | 454 | GS FLX Titanium | Single-end | 100-594 | EMO^2^ | SRR15620603 |
|  | mtDNA | Illumina | HiSeq 3000 | Paired-end | 150 | MPGC^3^ | SRR15620602 |
|  | mtDNA | Illumina | HiSeq 3000 | Mate-pair | 150 | MPGC^3^ | SRR15620601 |
|  | mRNA^1^ | Illumina | HiSeq 4000 | Paired-end | 75 | MPIMG^4^ | SRR15620600 |
|  | total DNA | Pacific Biosciences | RS II | Single-end | 1000-73887 | MPGC^3^ | NA |

^1^ rRNA depleted

^2^ Eurofins MWG Operon

^3^ Max Planck Genome Centre Cologne

^4^ Max Planck Institute for Molecular Genetics
