# Supplementary figures and images for: Graph-based models of the Oenothera mitochondrial genome capture the enormous complexity of higher plant mitochondrial DNA organization
Source: NAR Genom Bioinform. 2022 Mar 31;4(2):lqac027. doi: 10.1093/nargab/lqac027 (PMC8969700; doi:10.1093/nargab/lqac027)

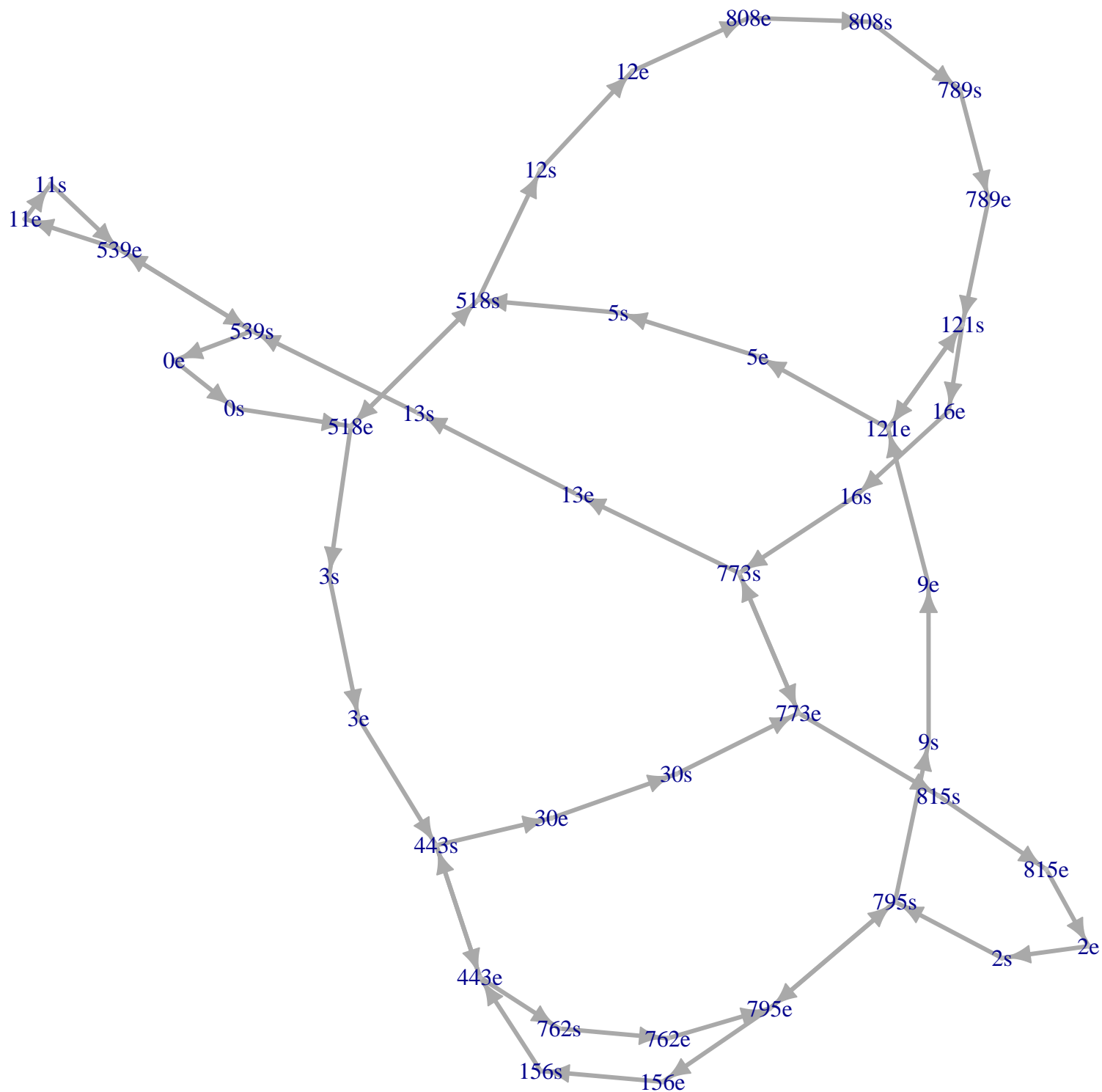

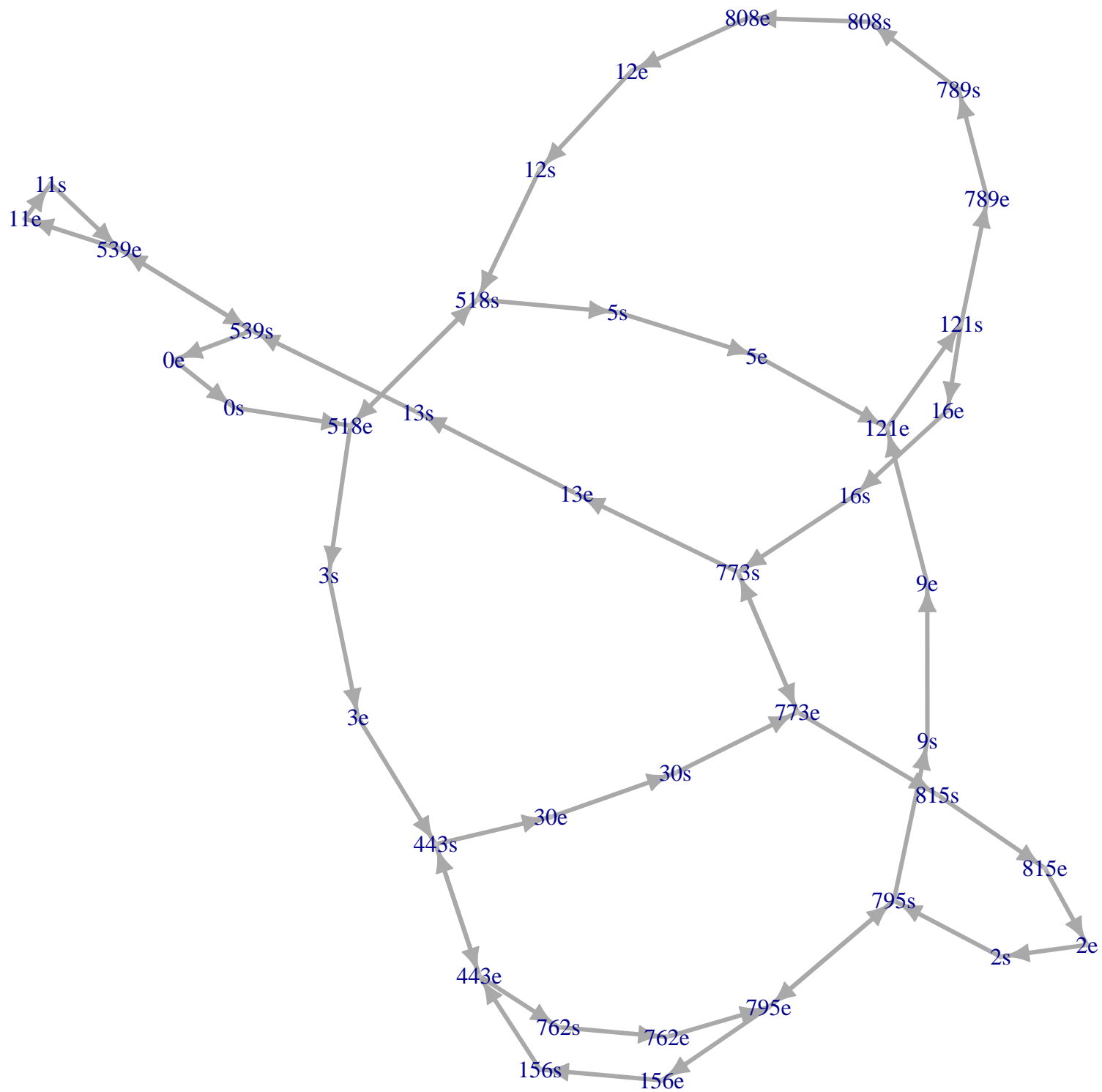

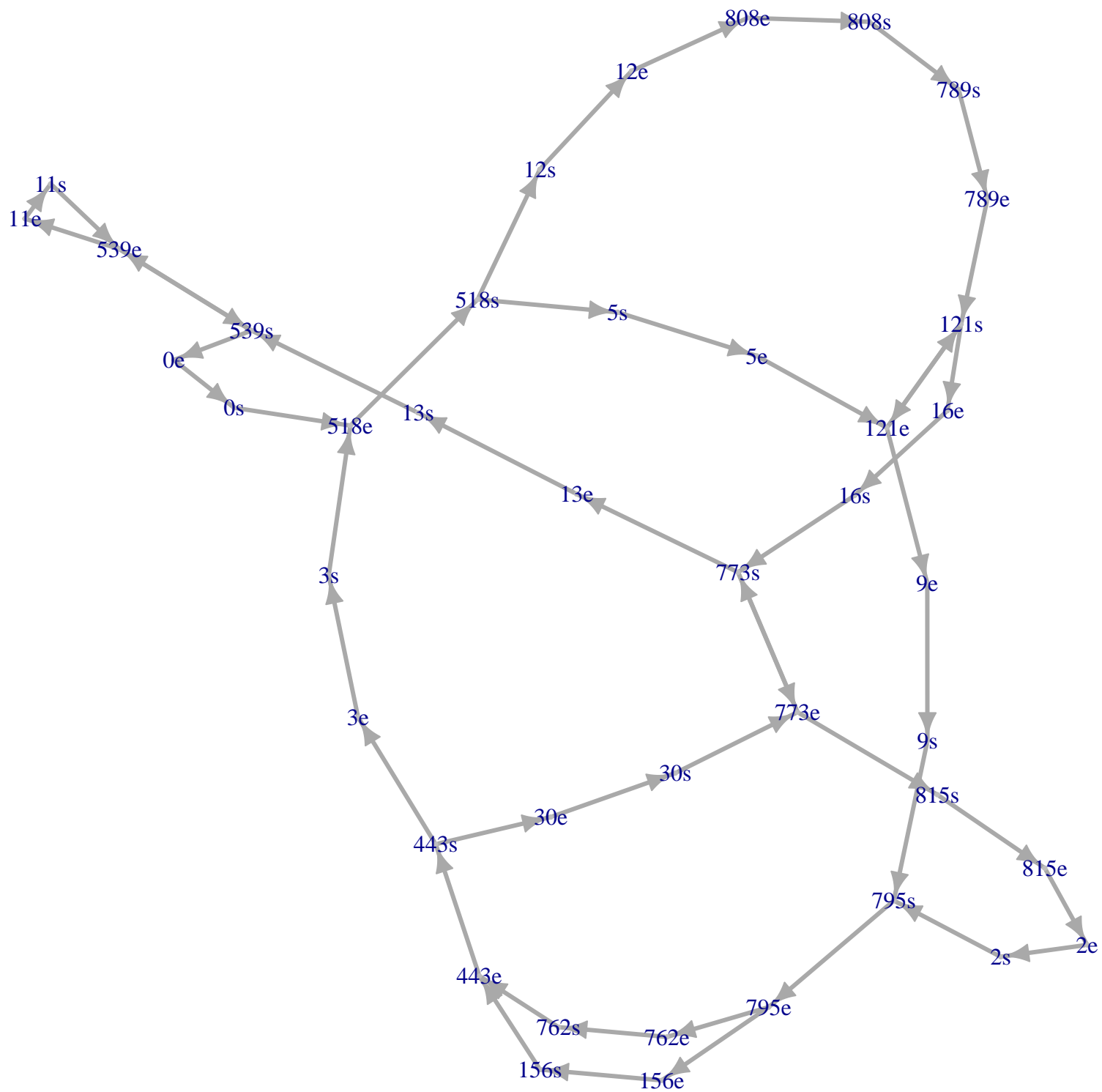

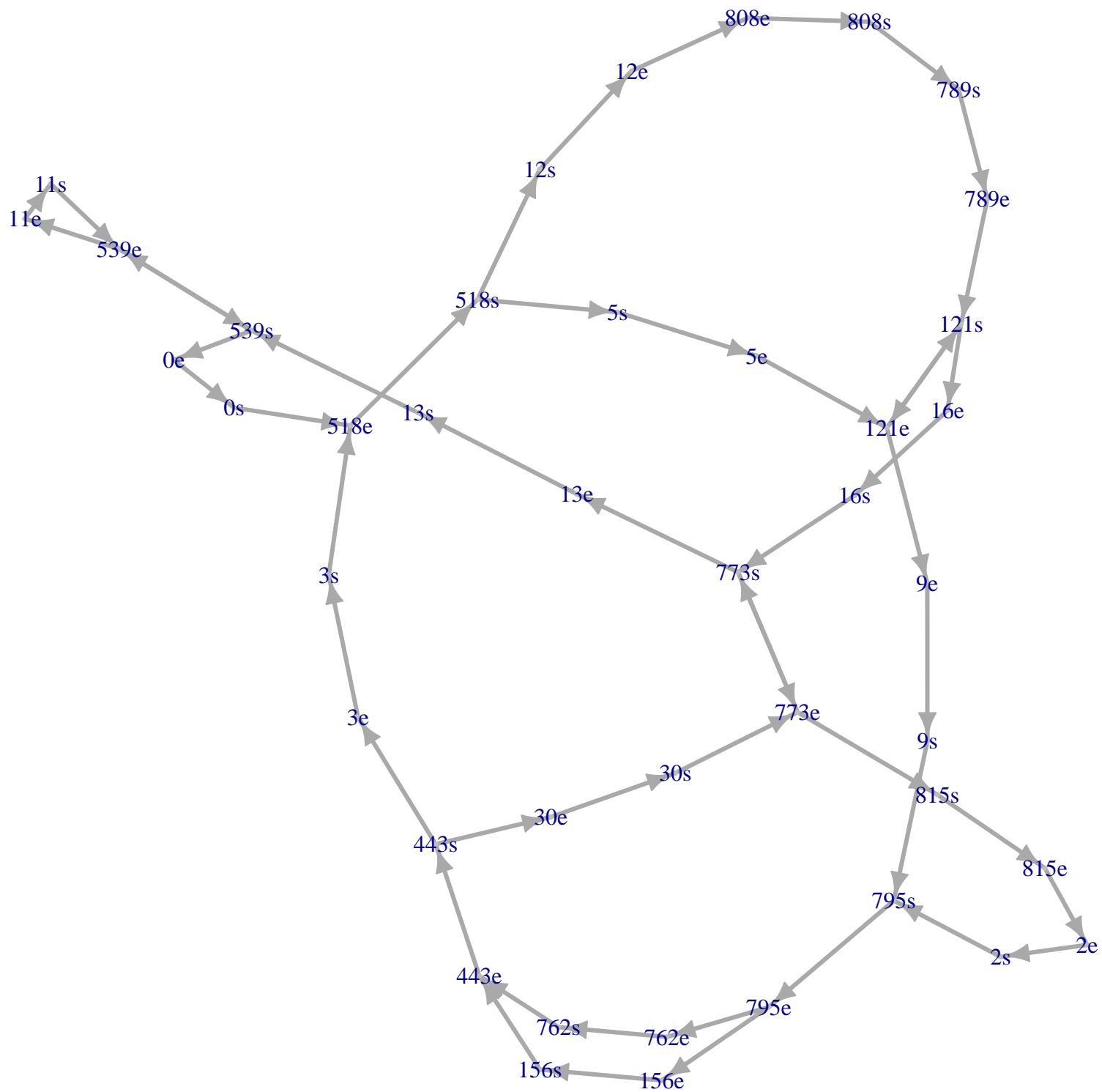

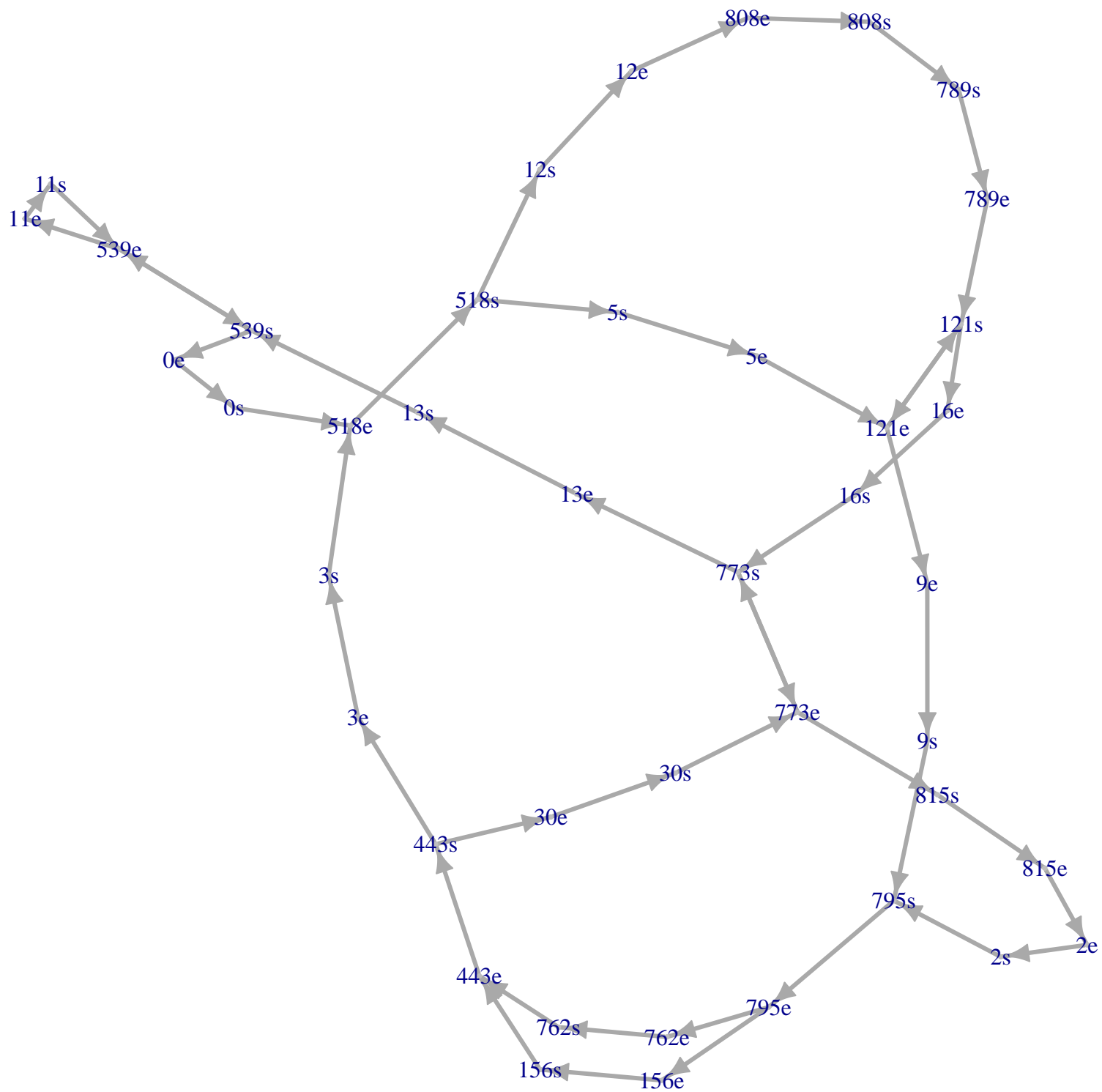

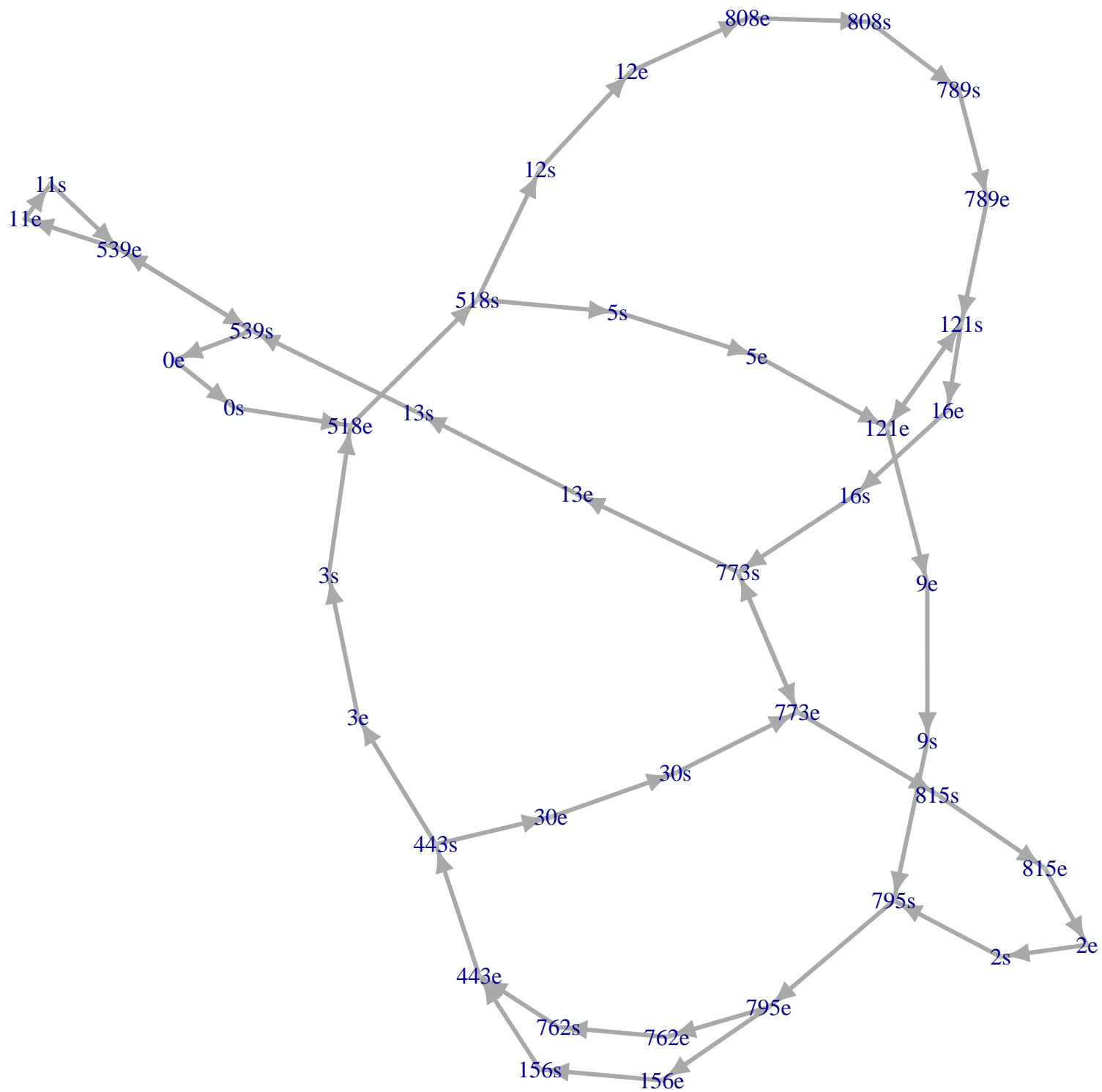

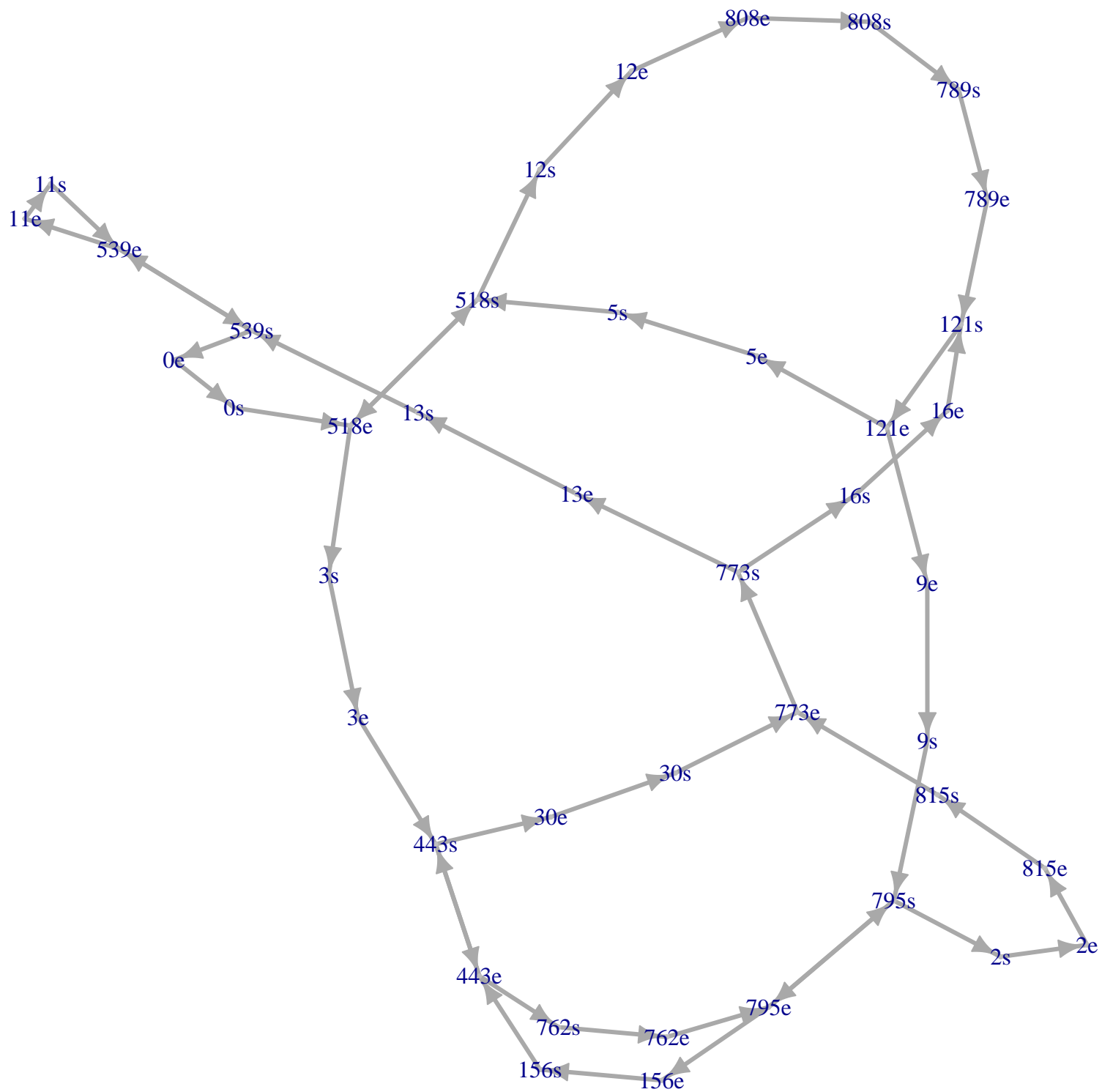

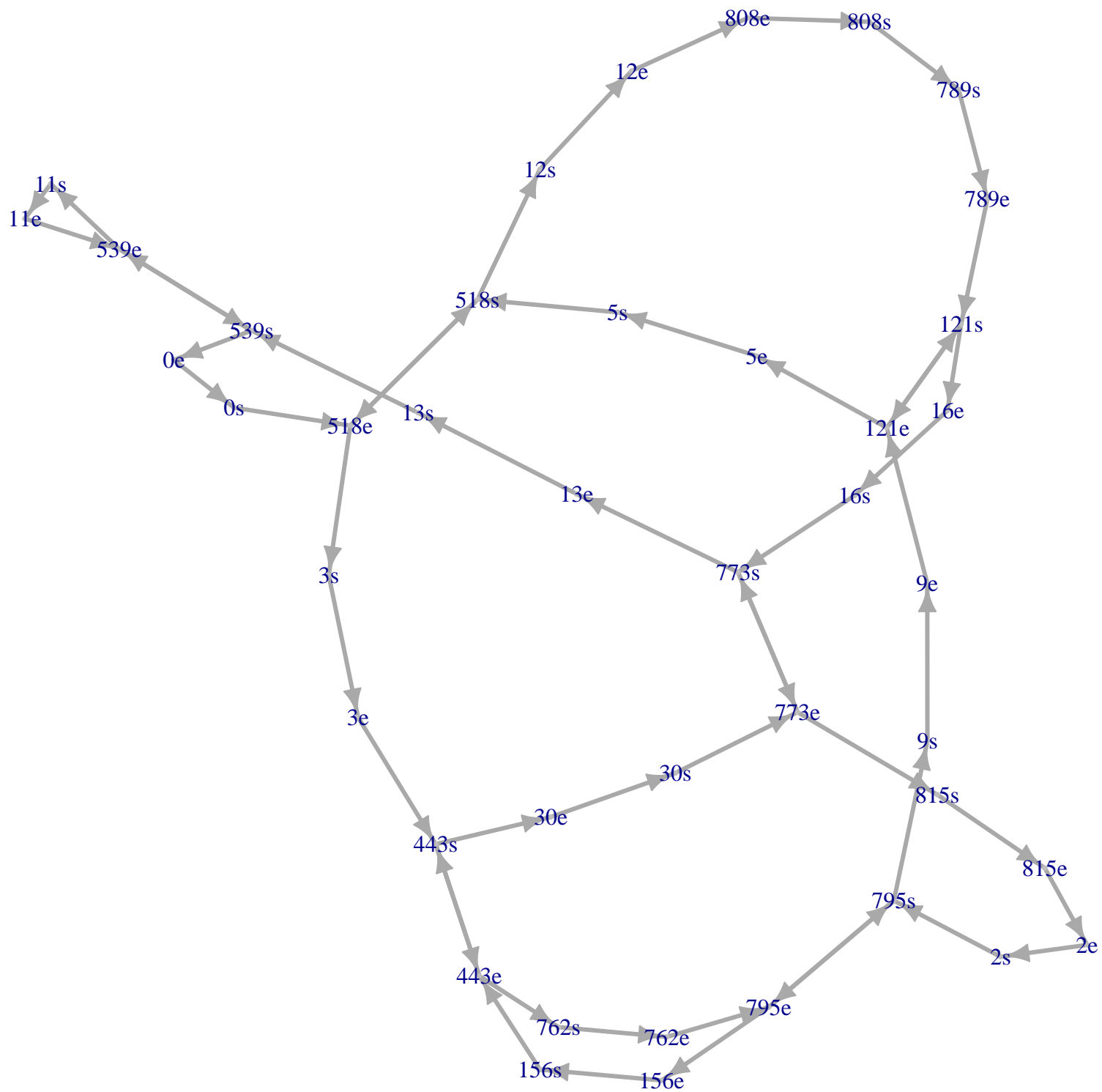

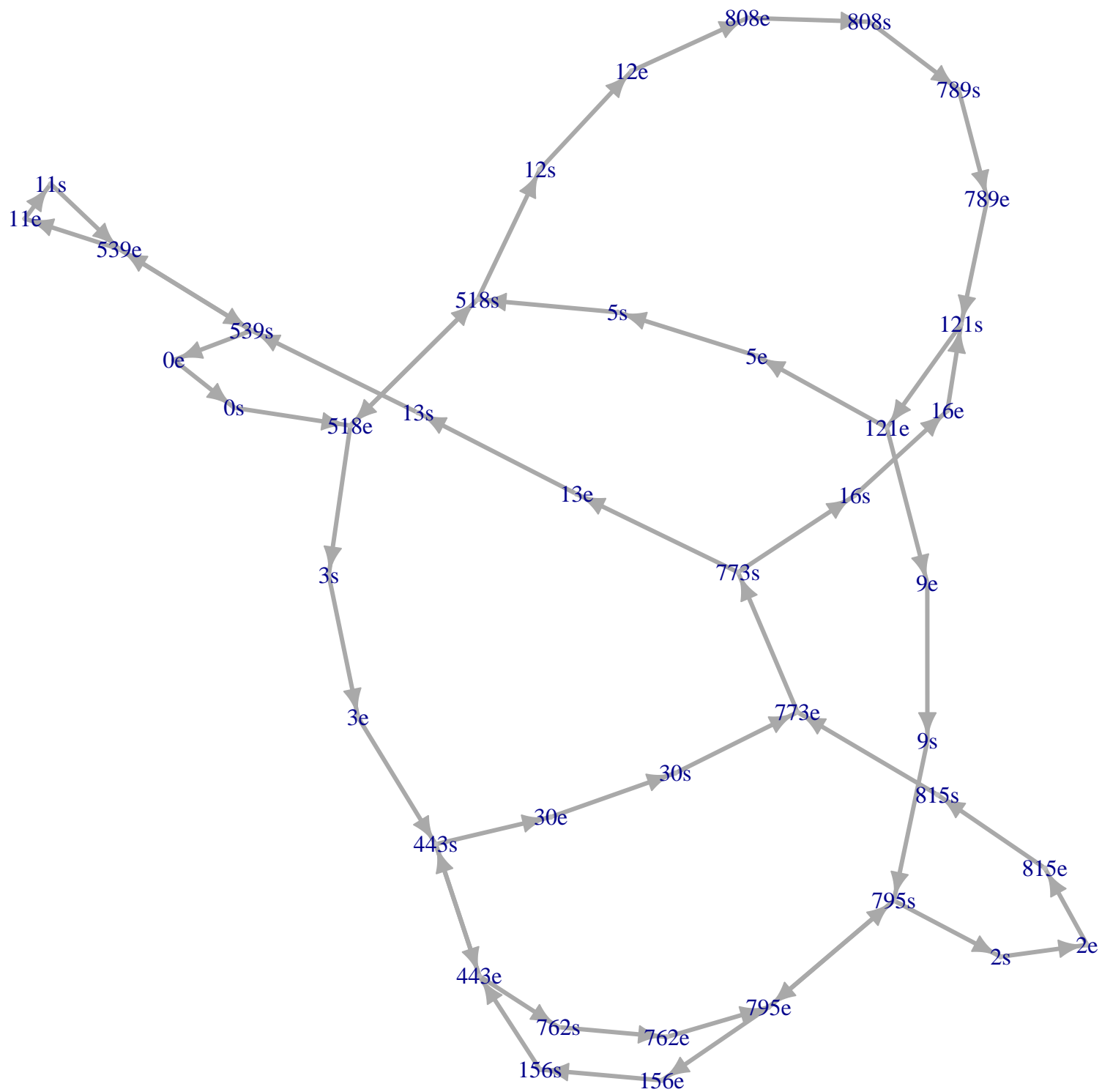

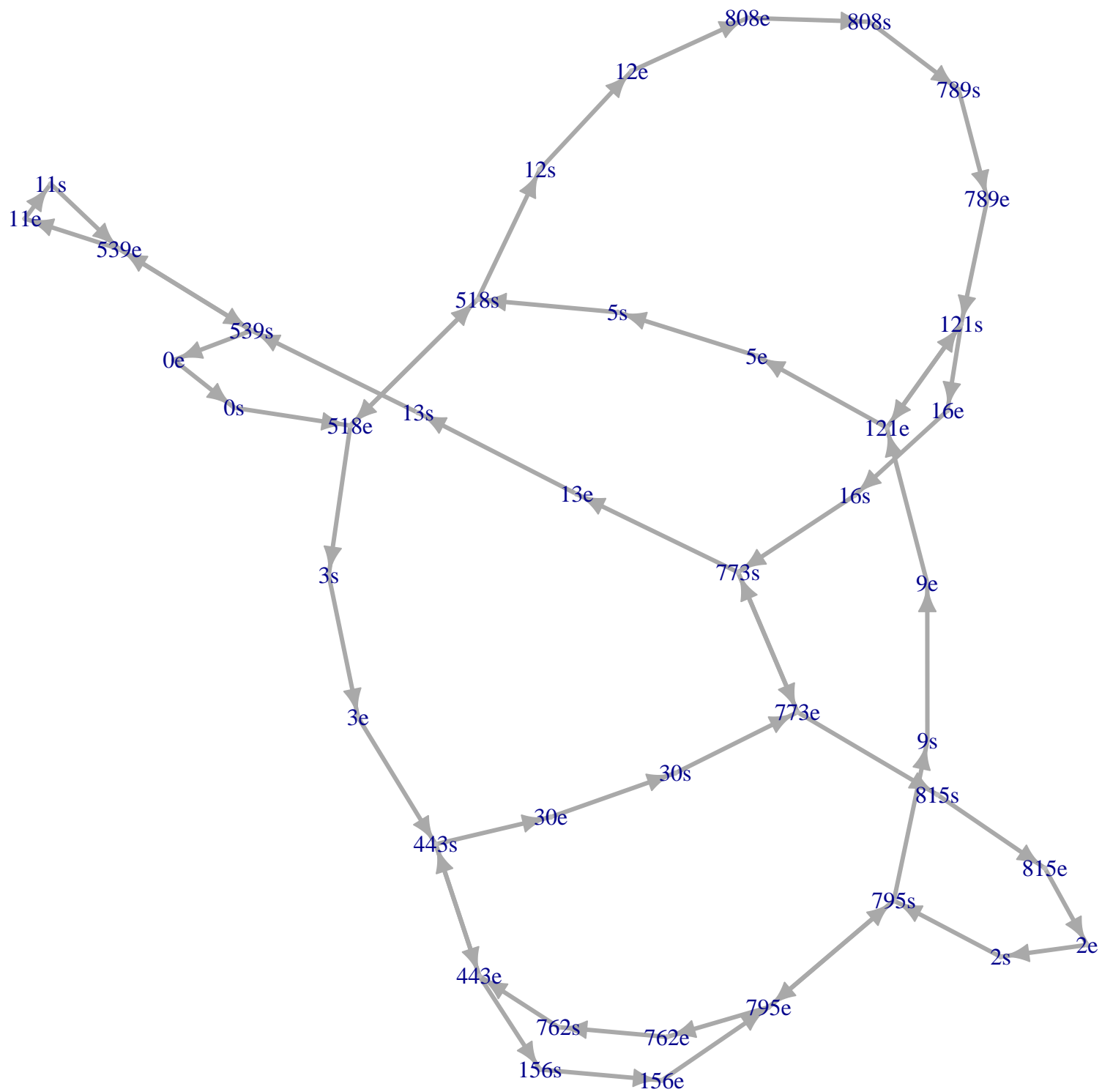

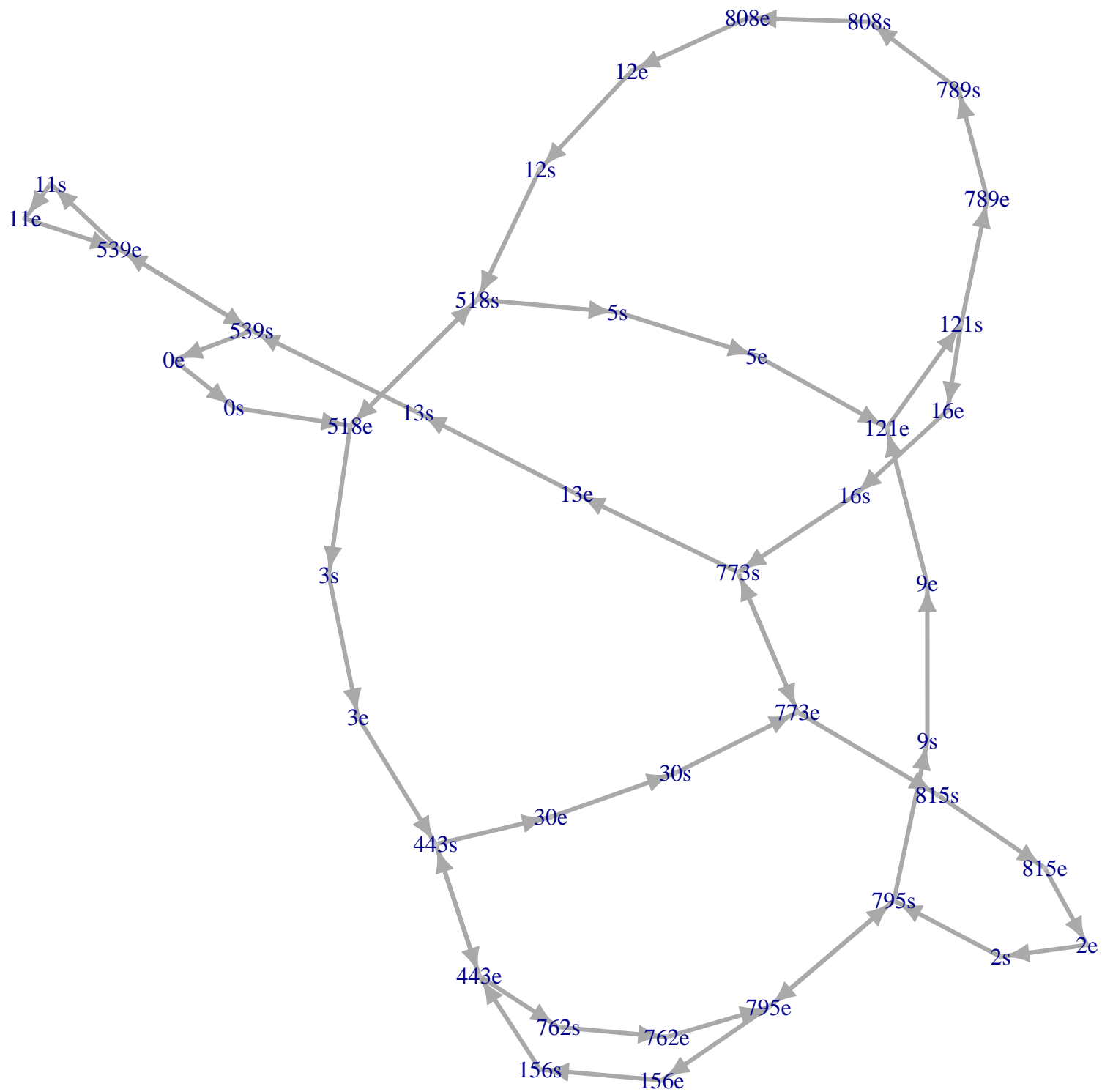

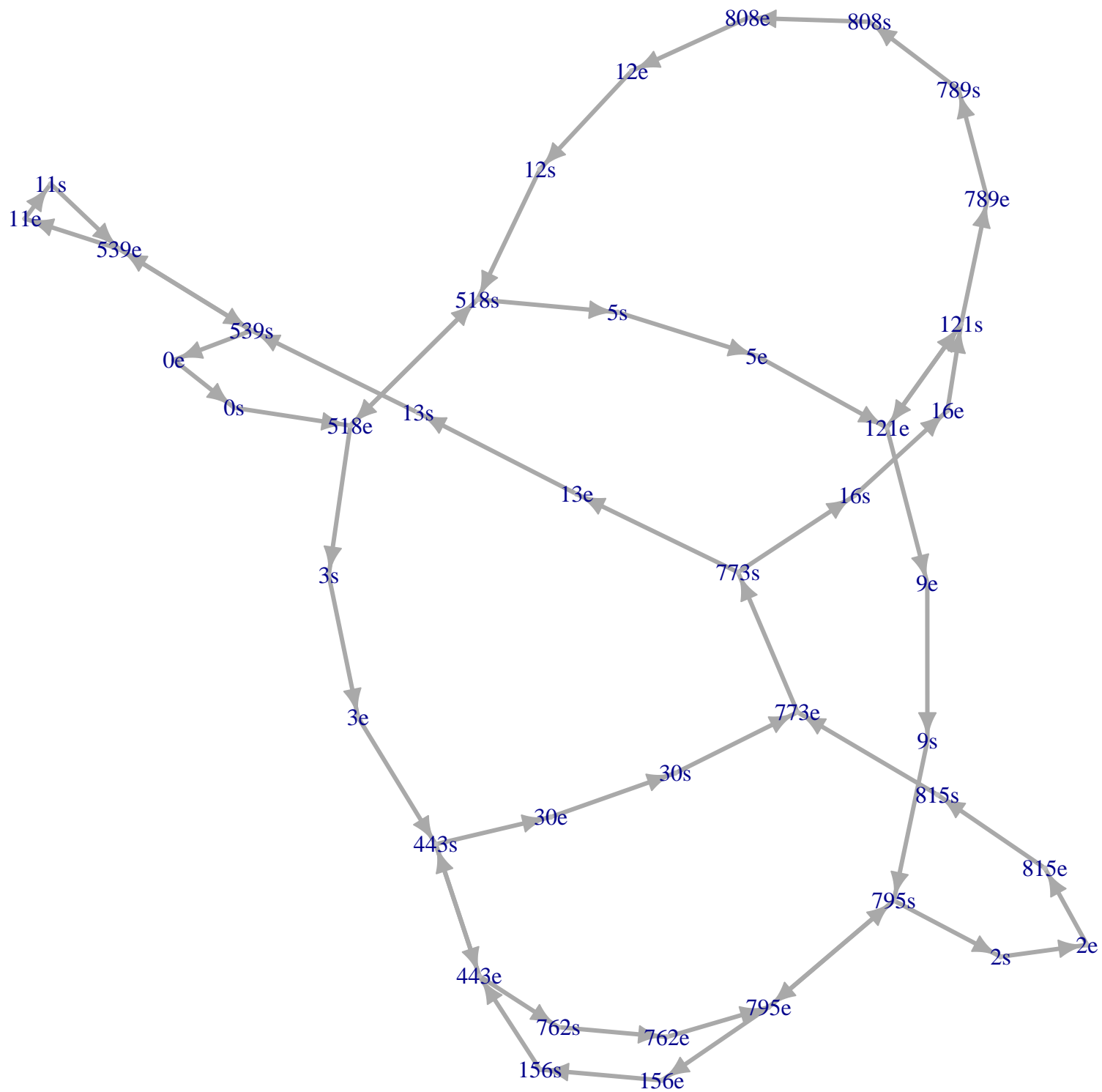

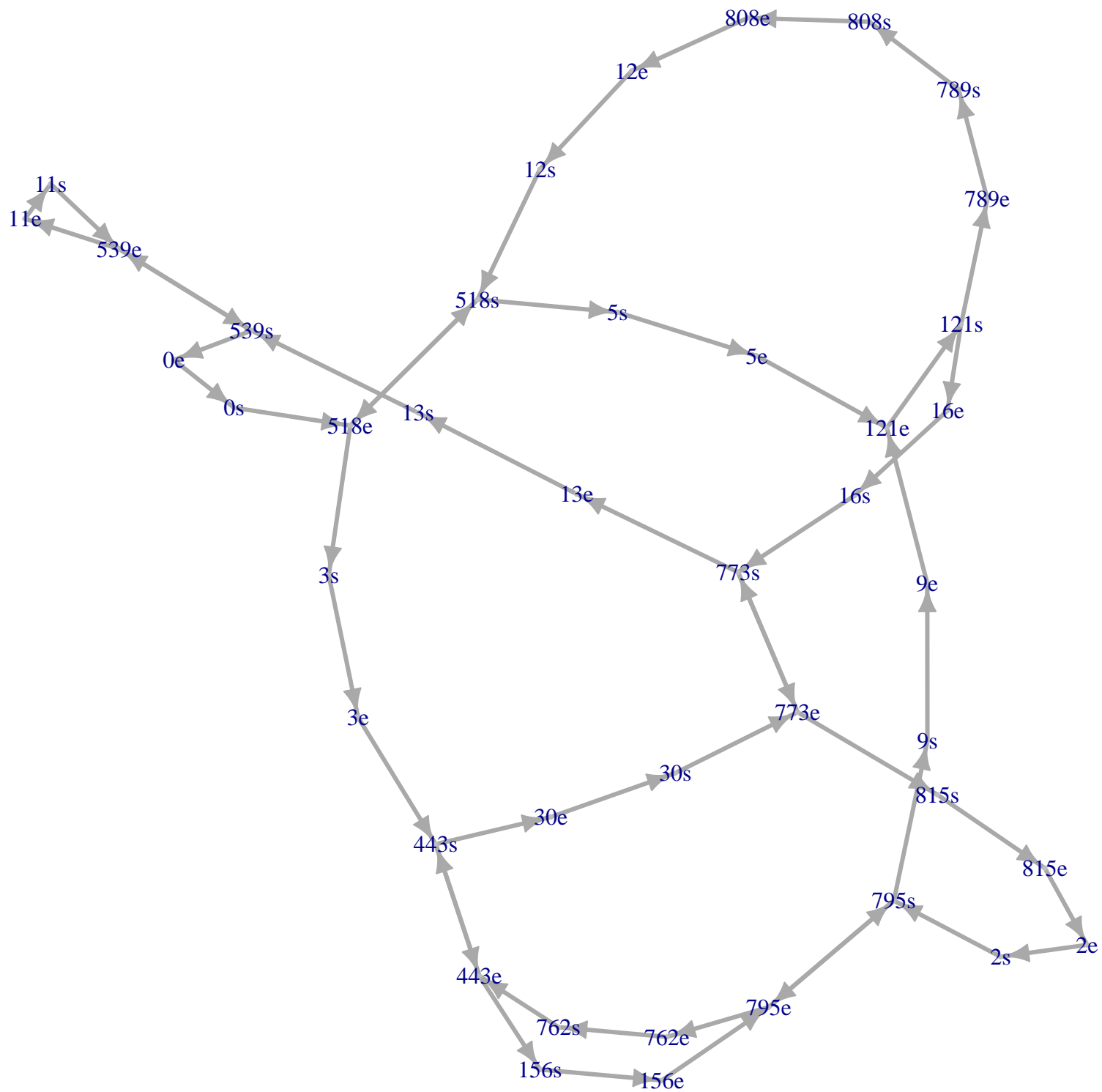

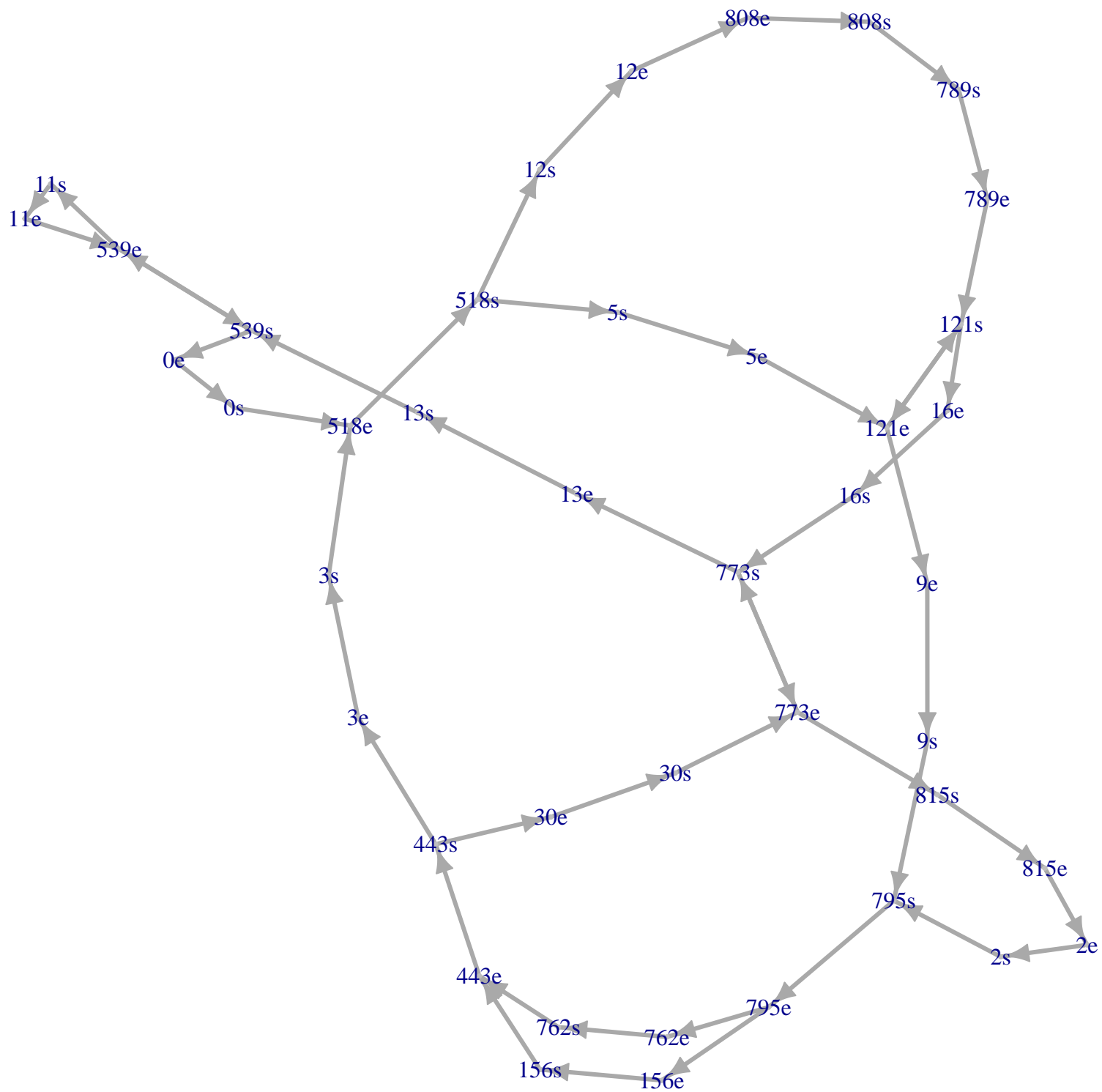

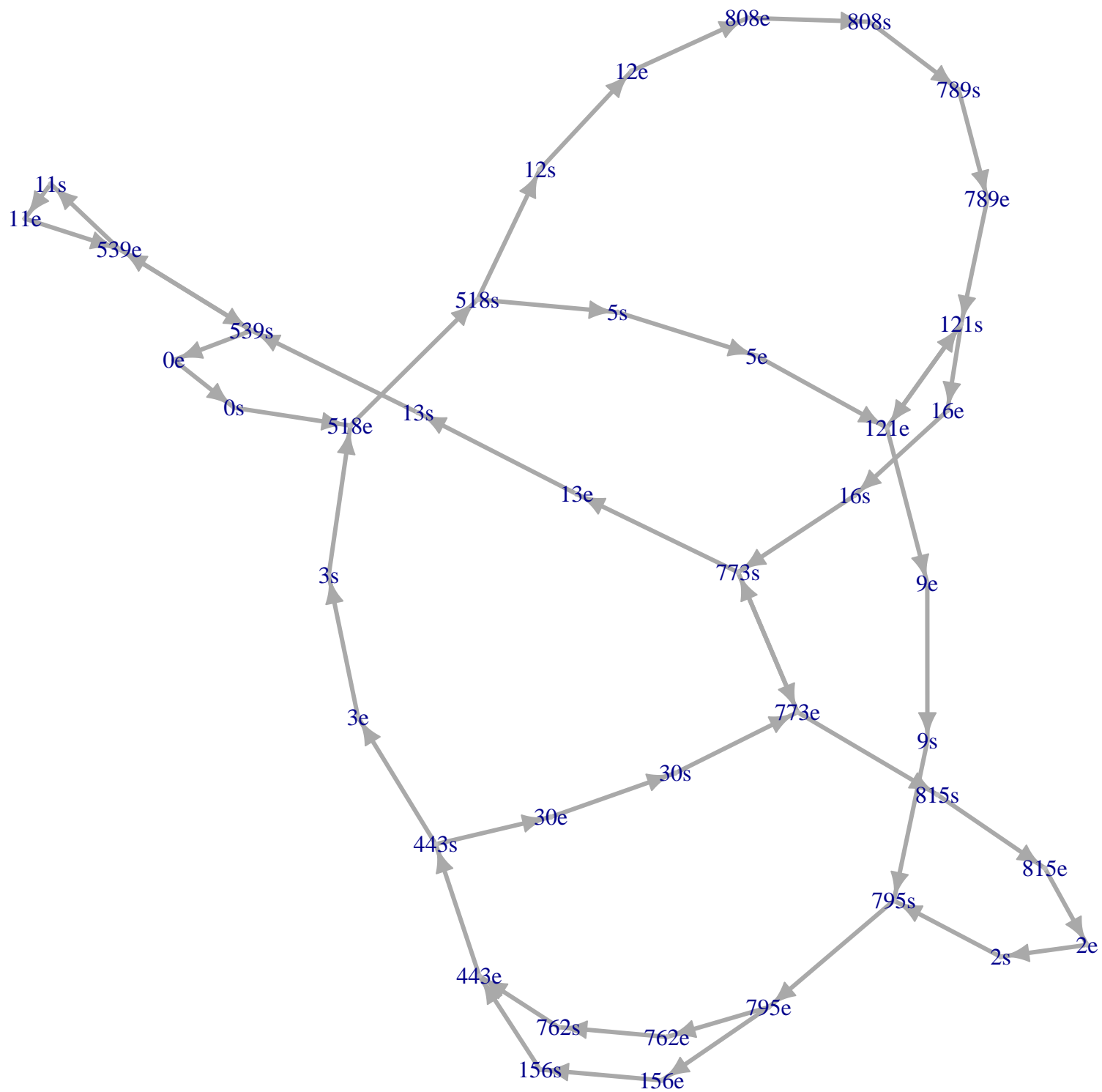

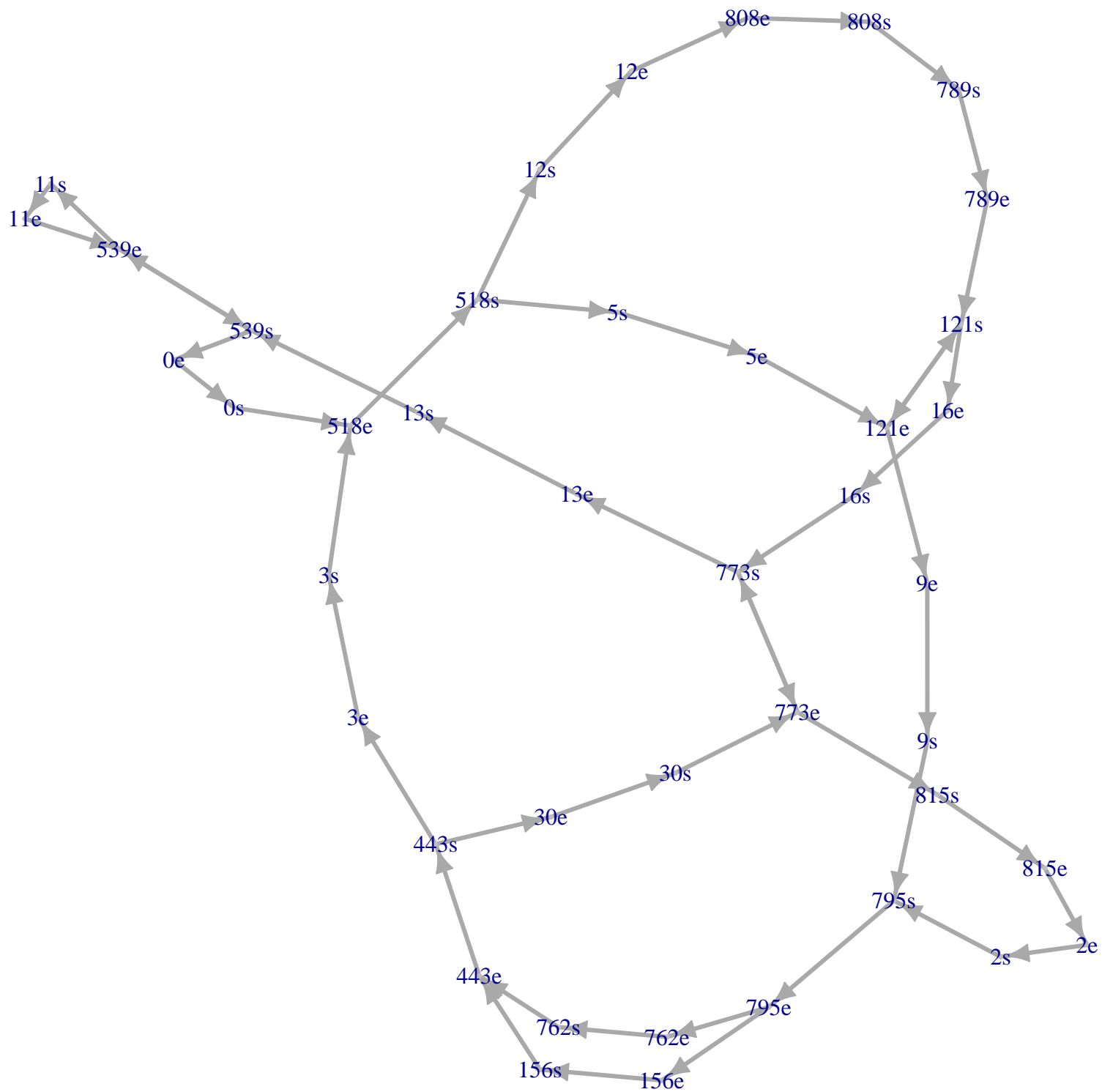

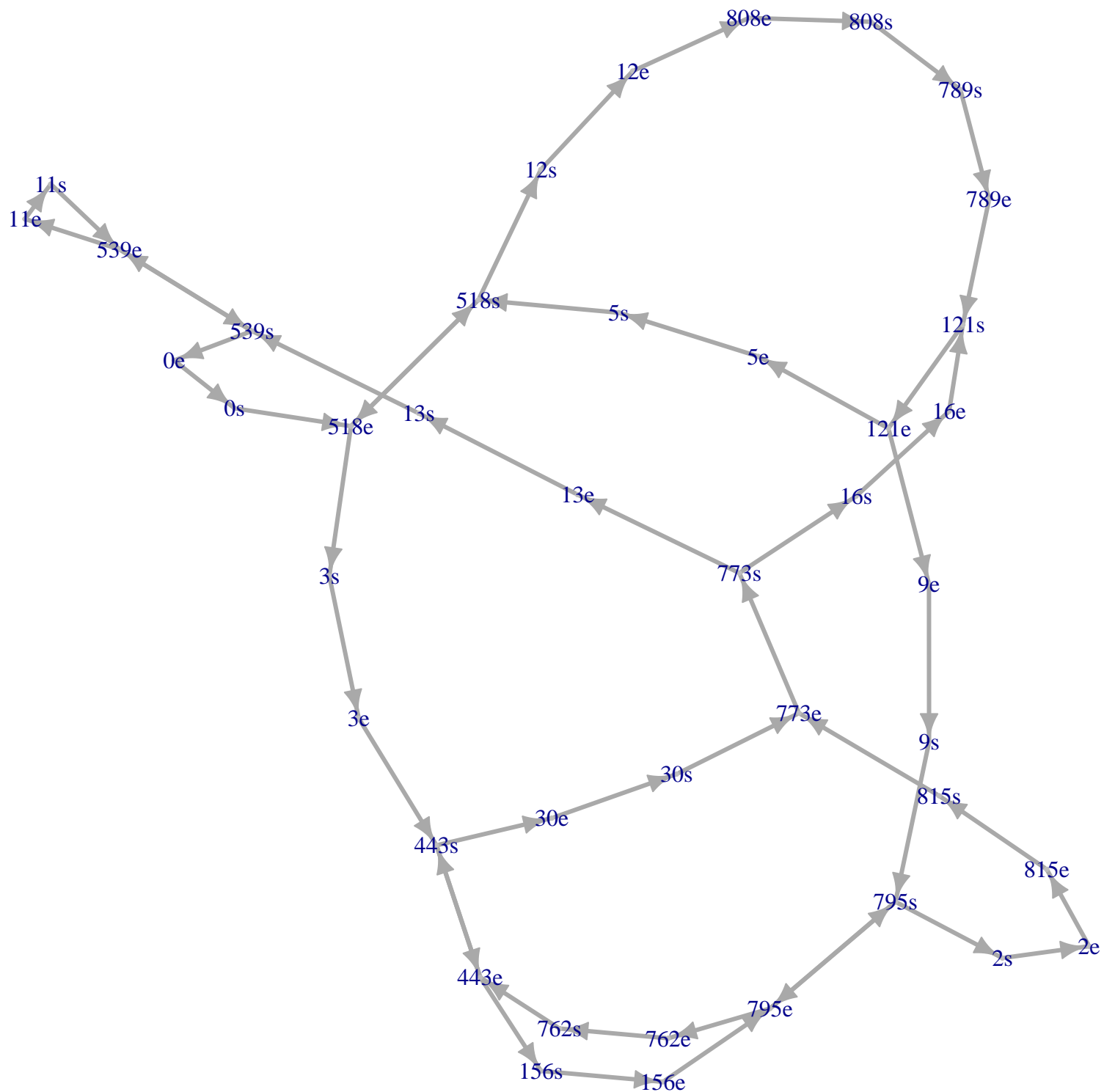

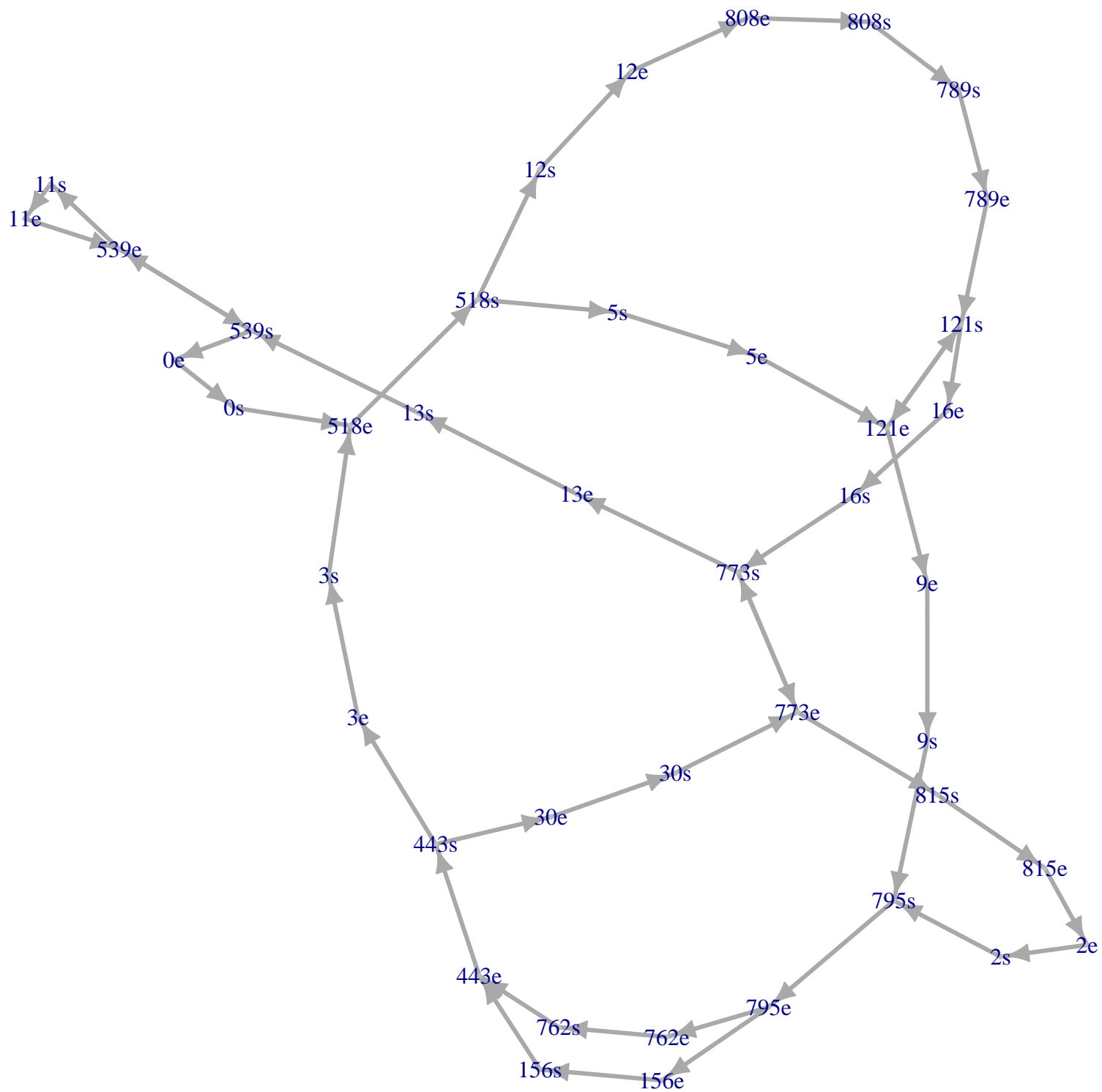

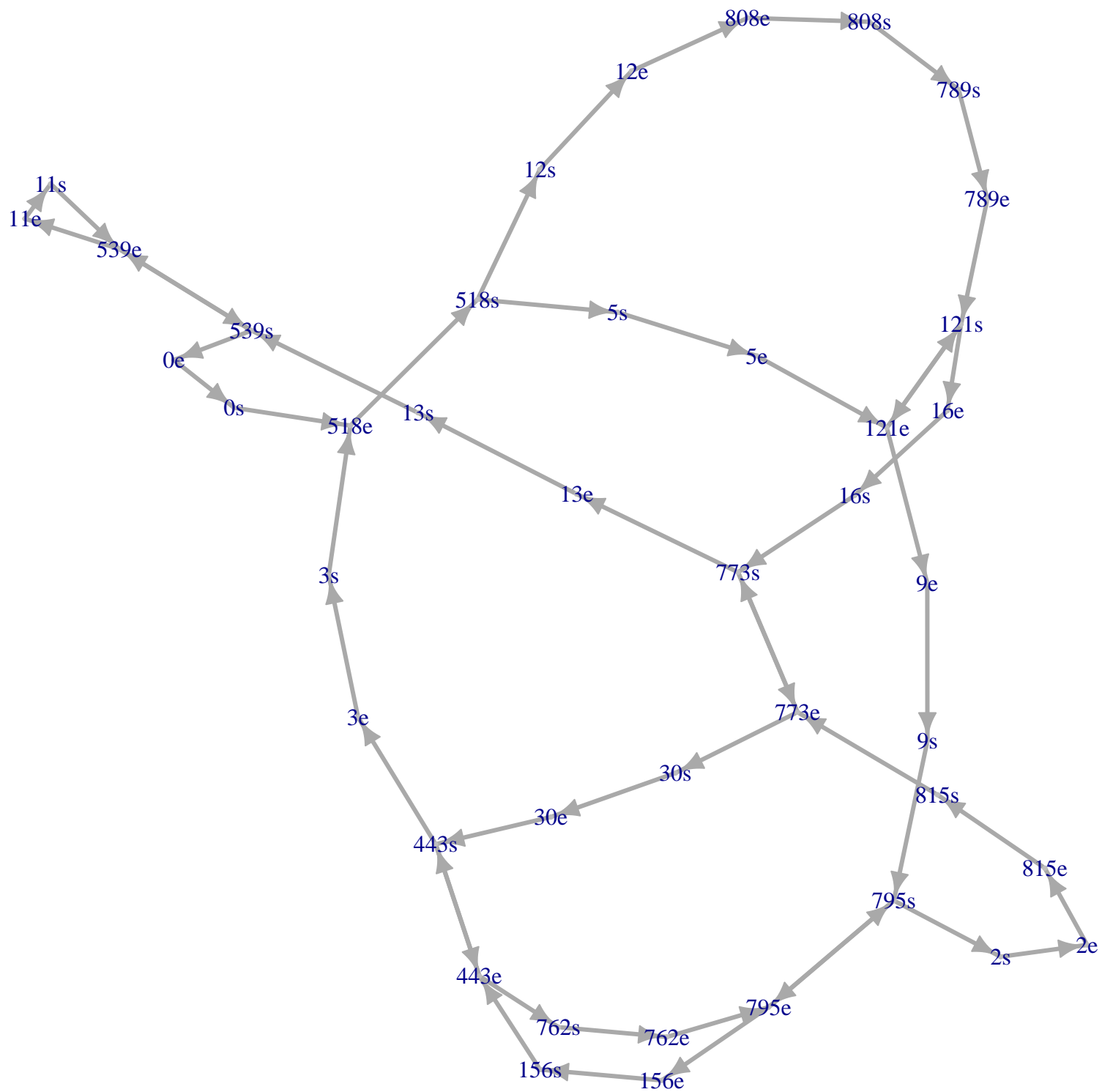

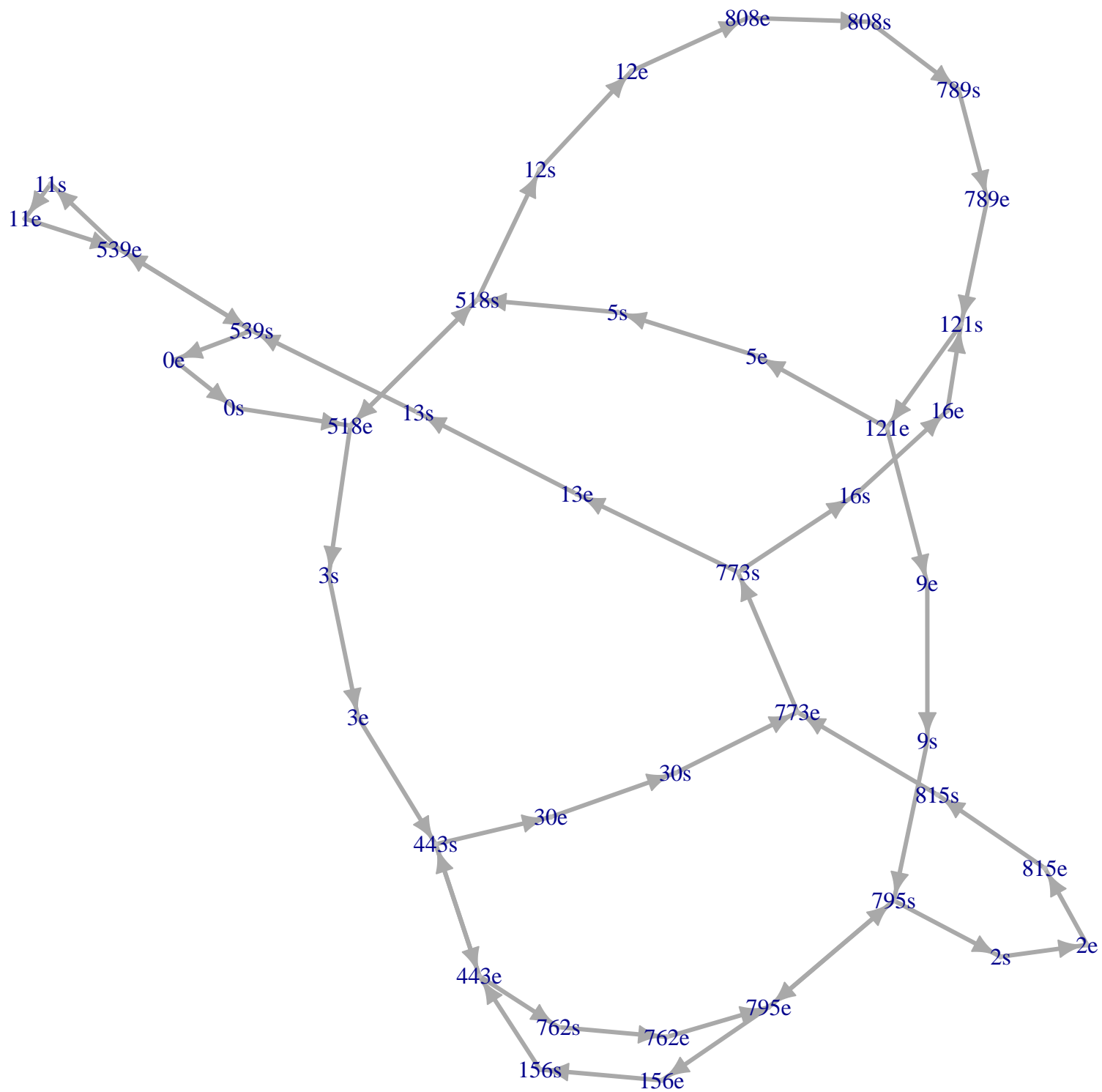

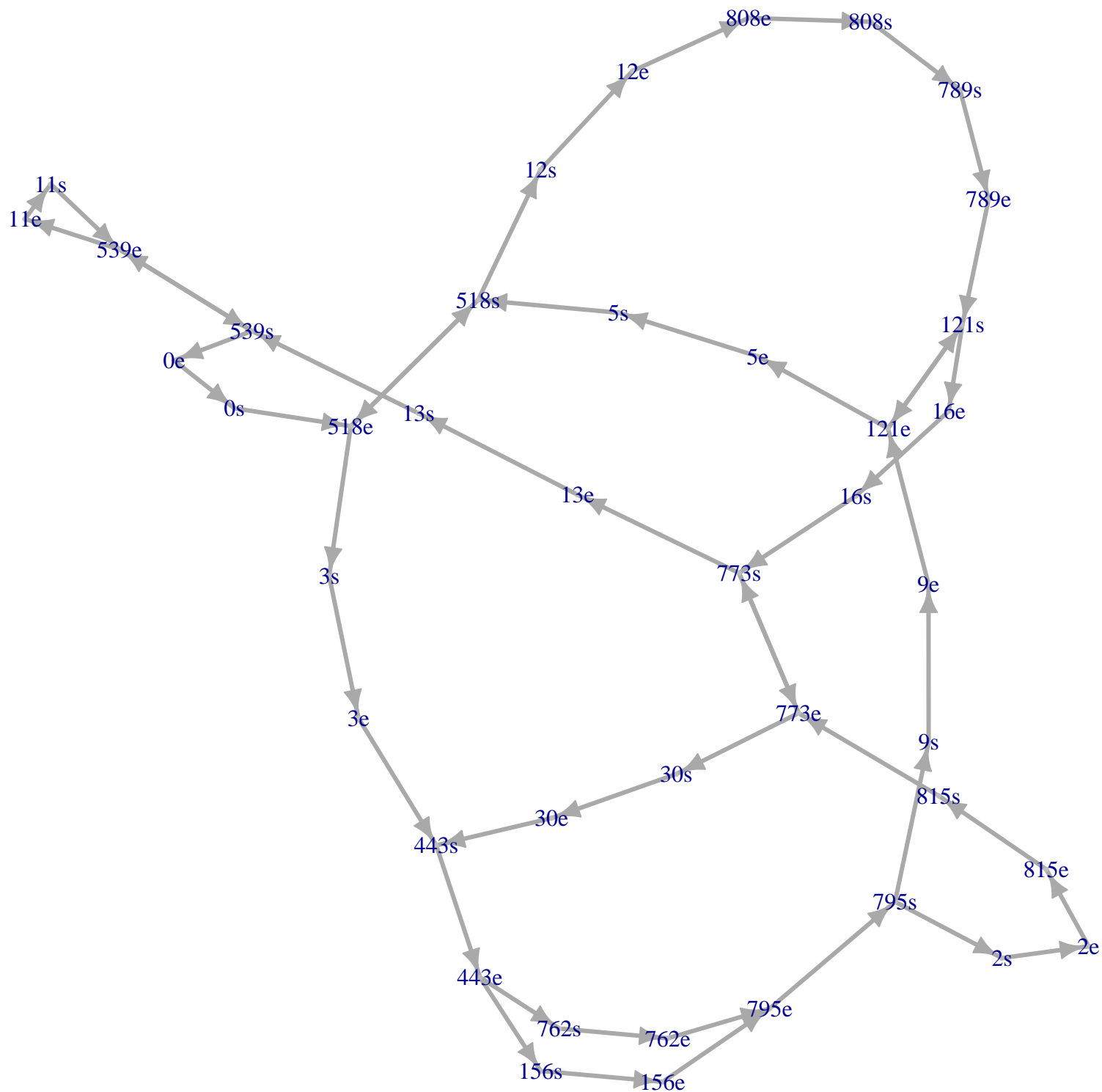

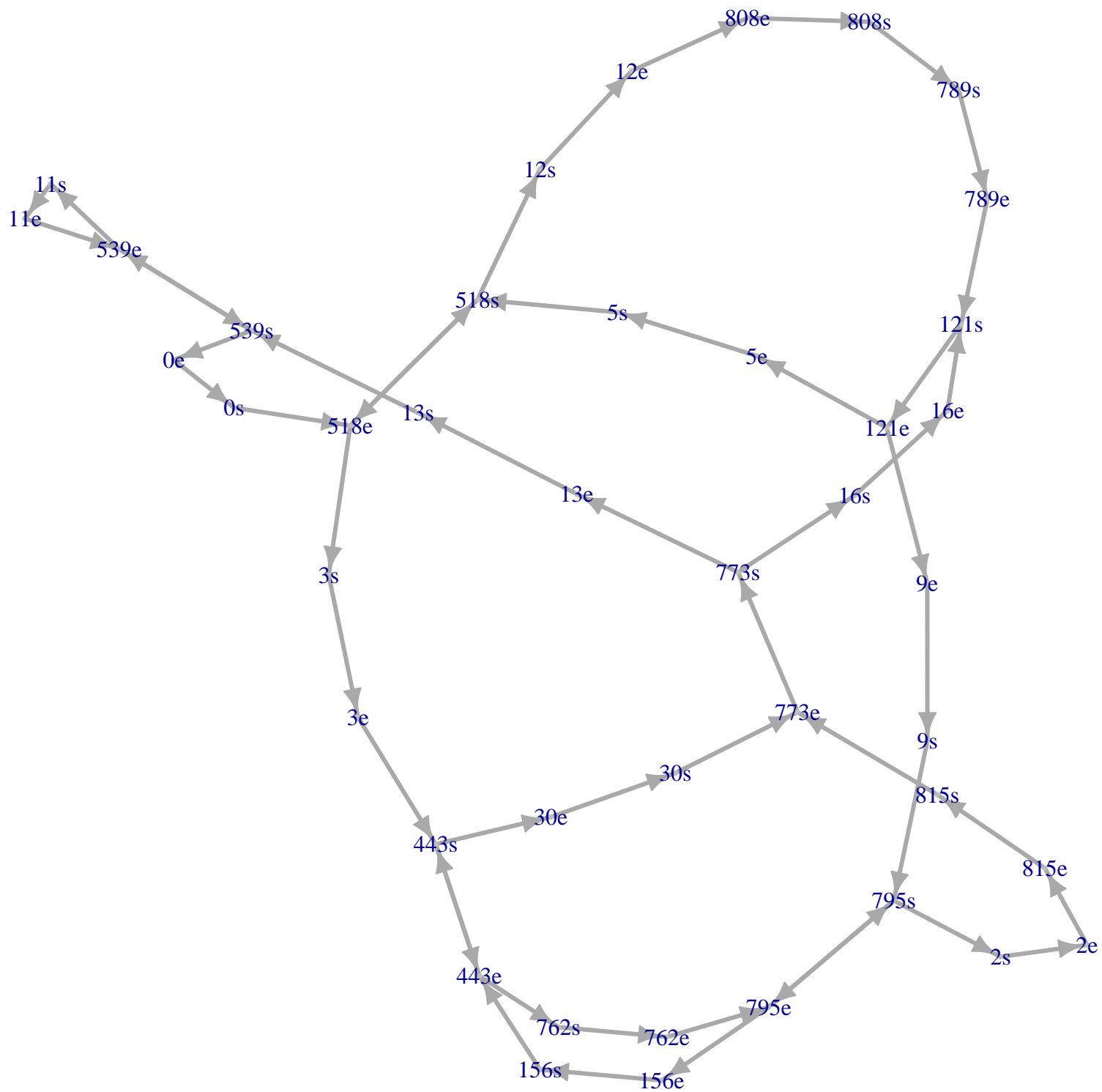

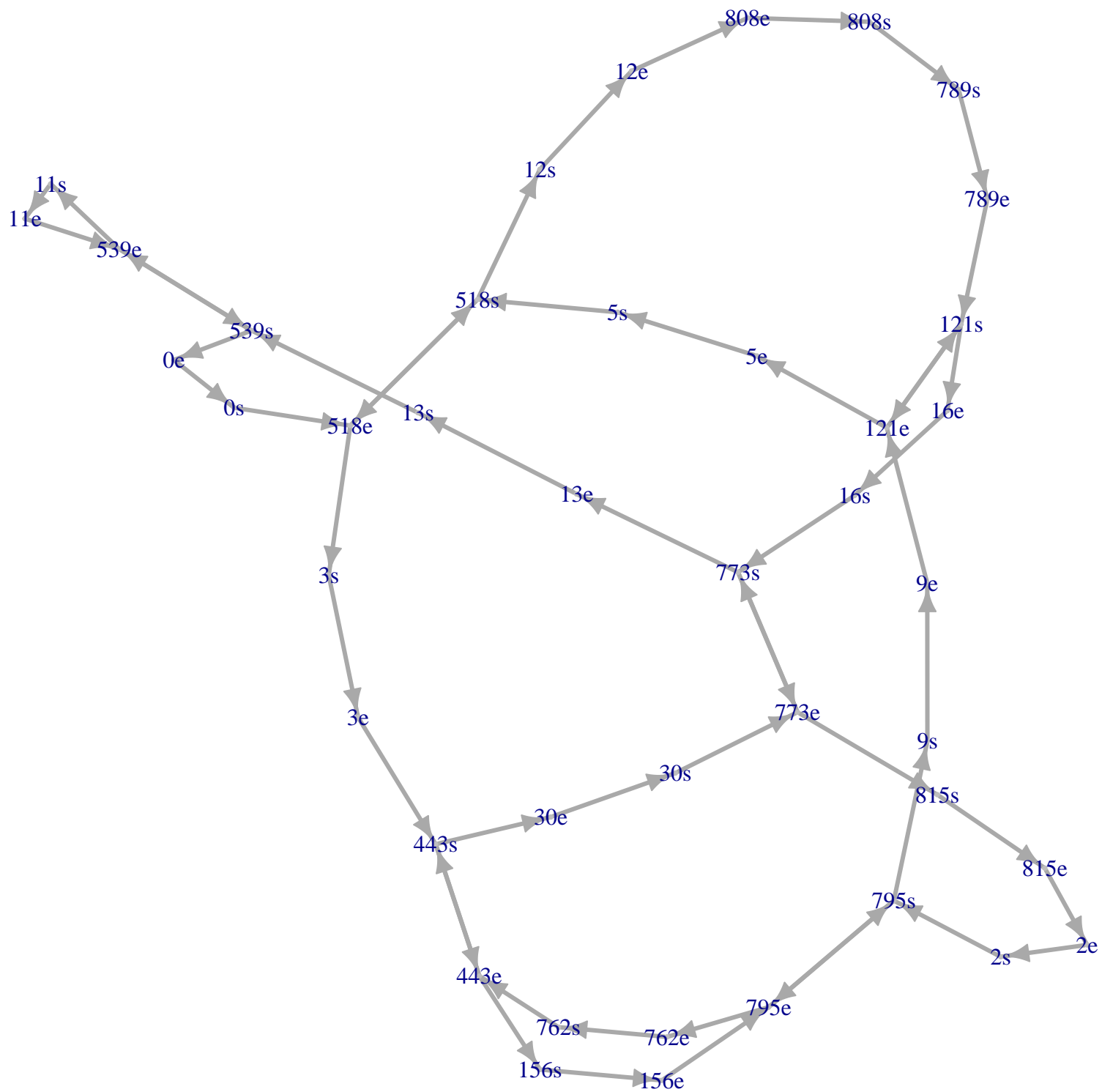

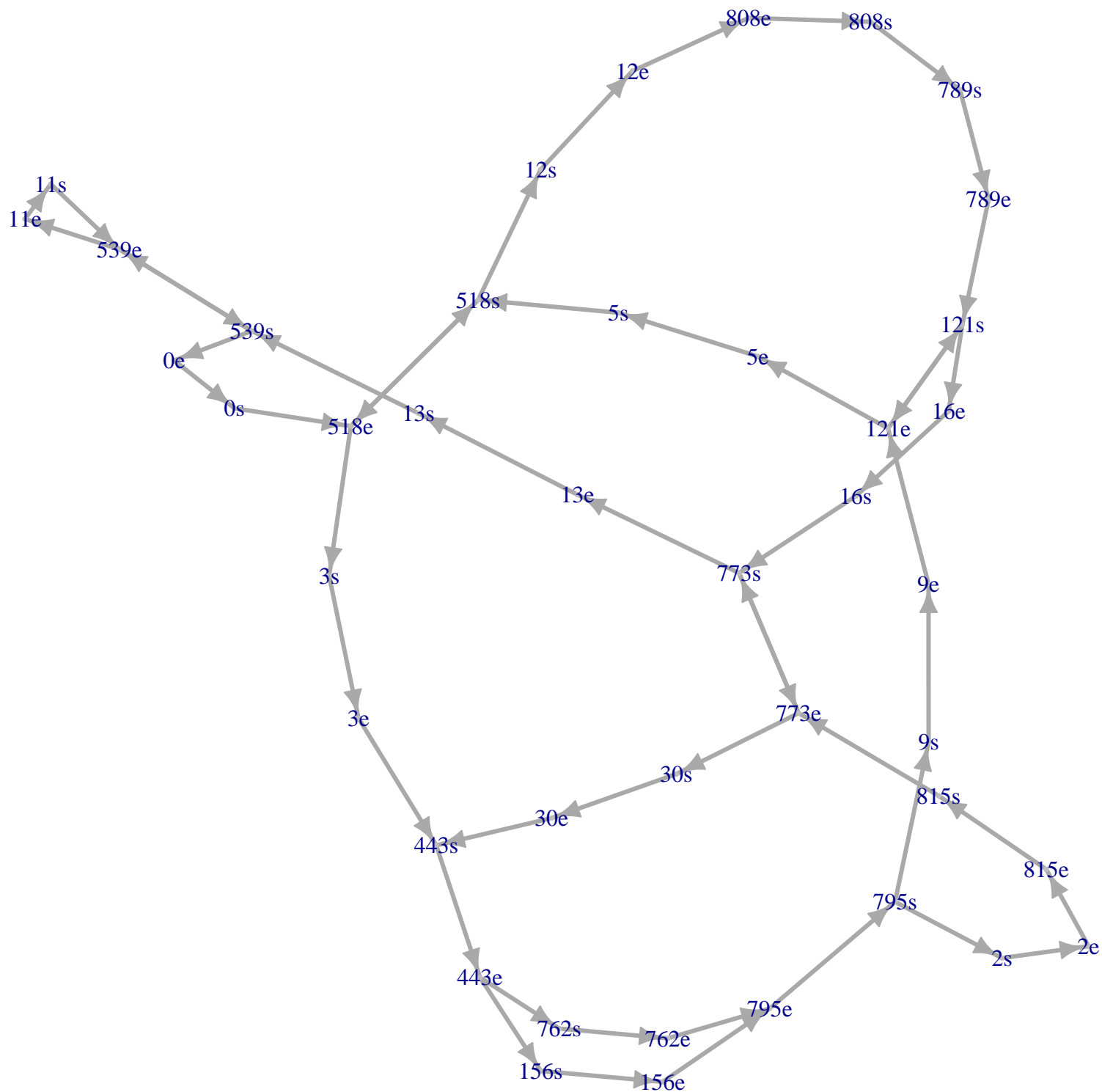

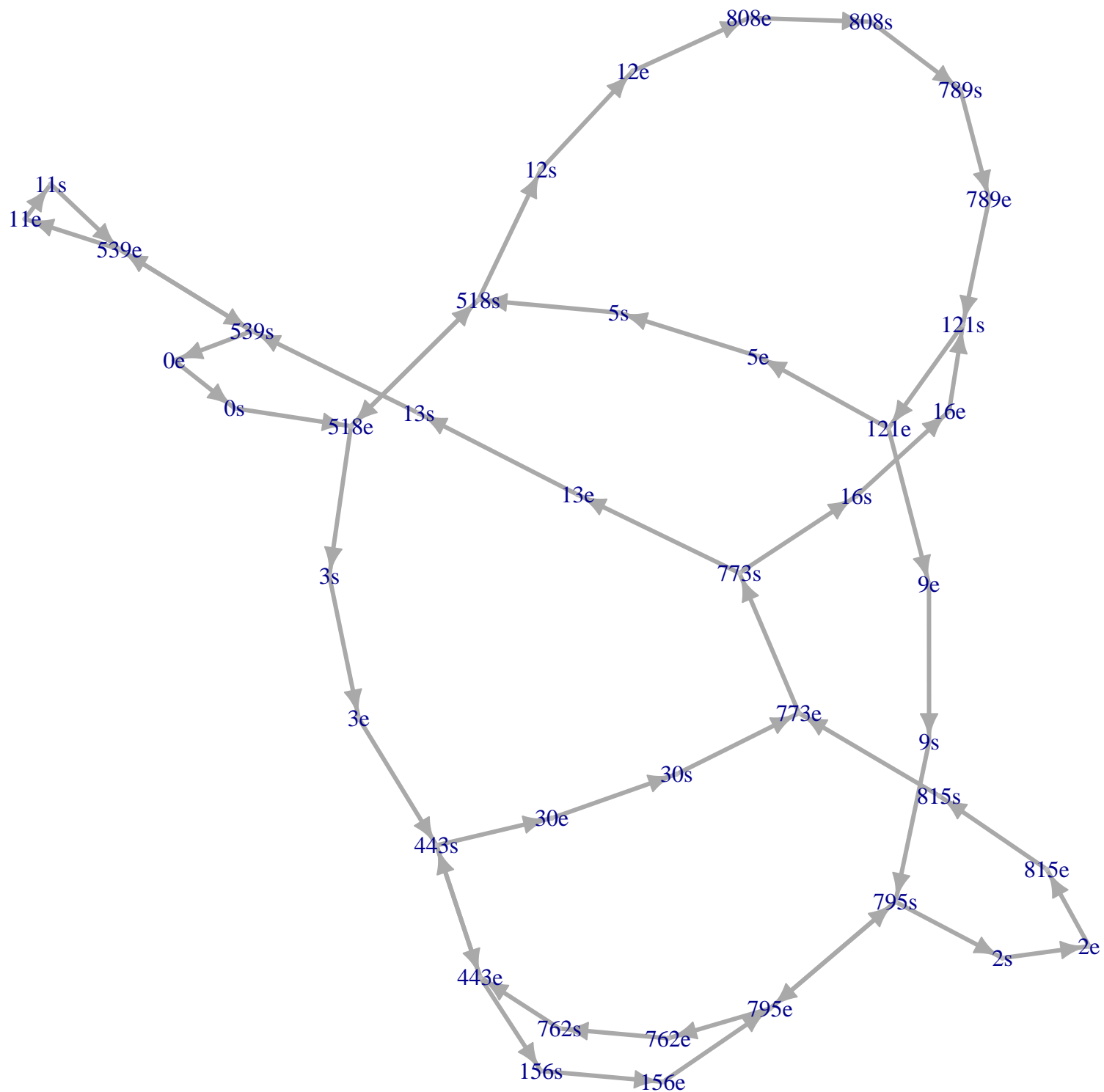

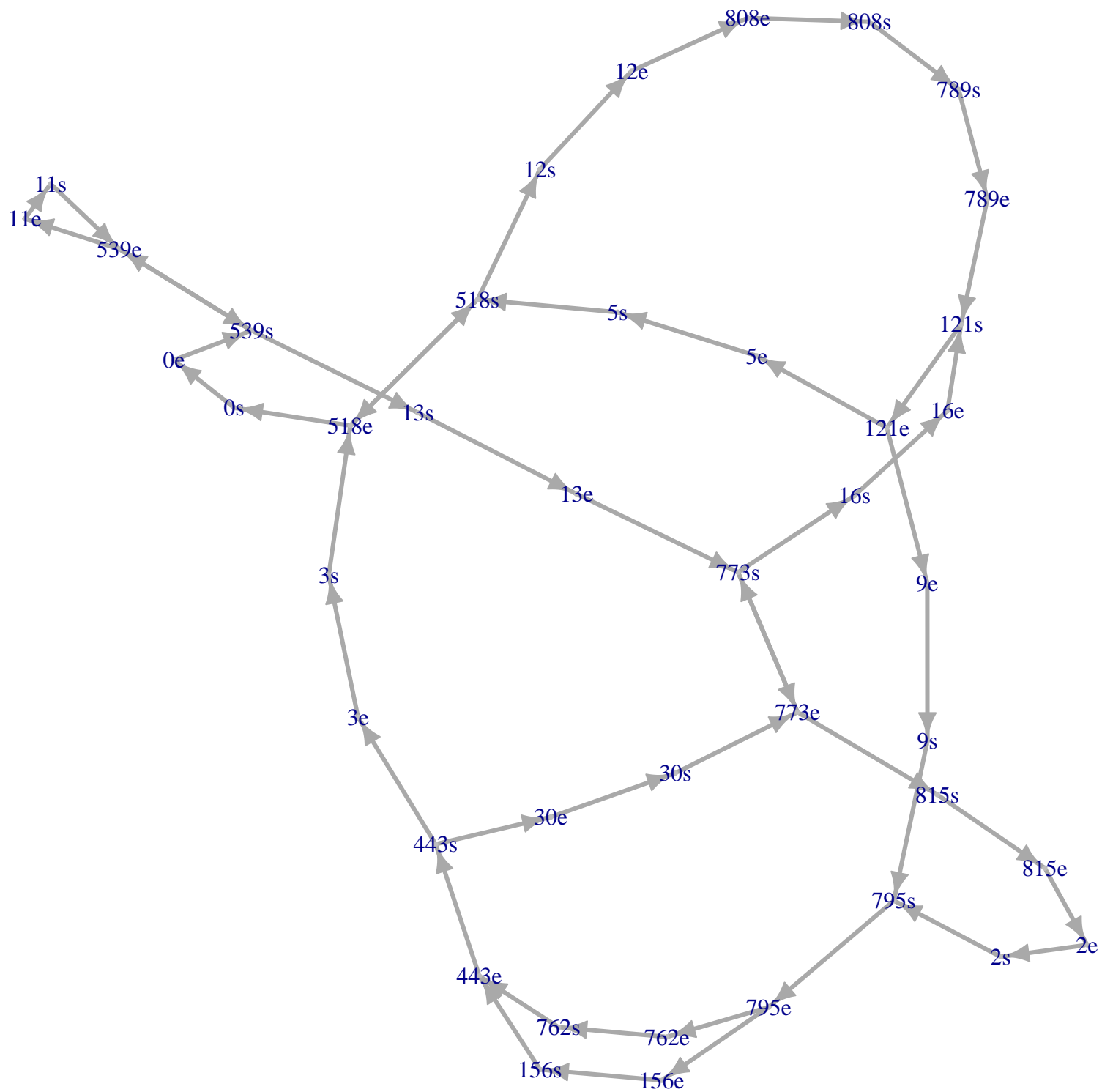

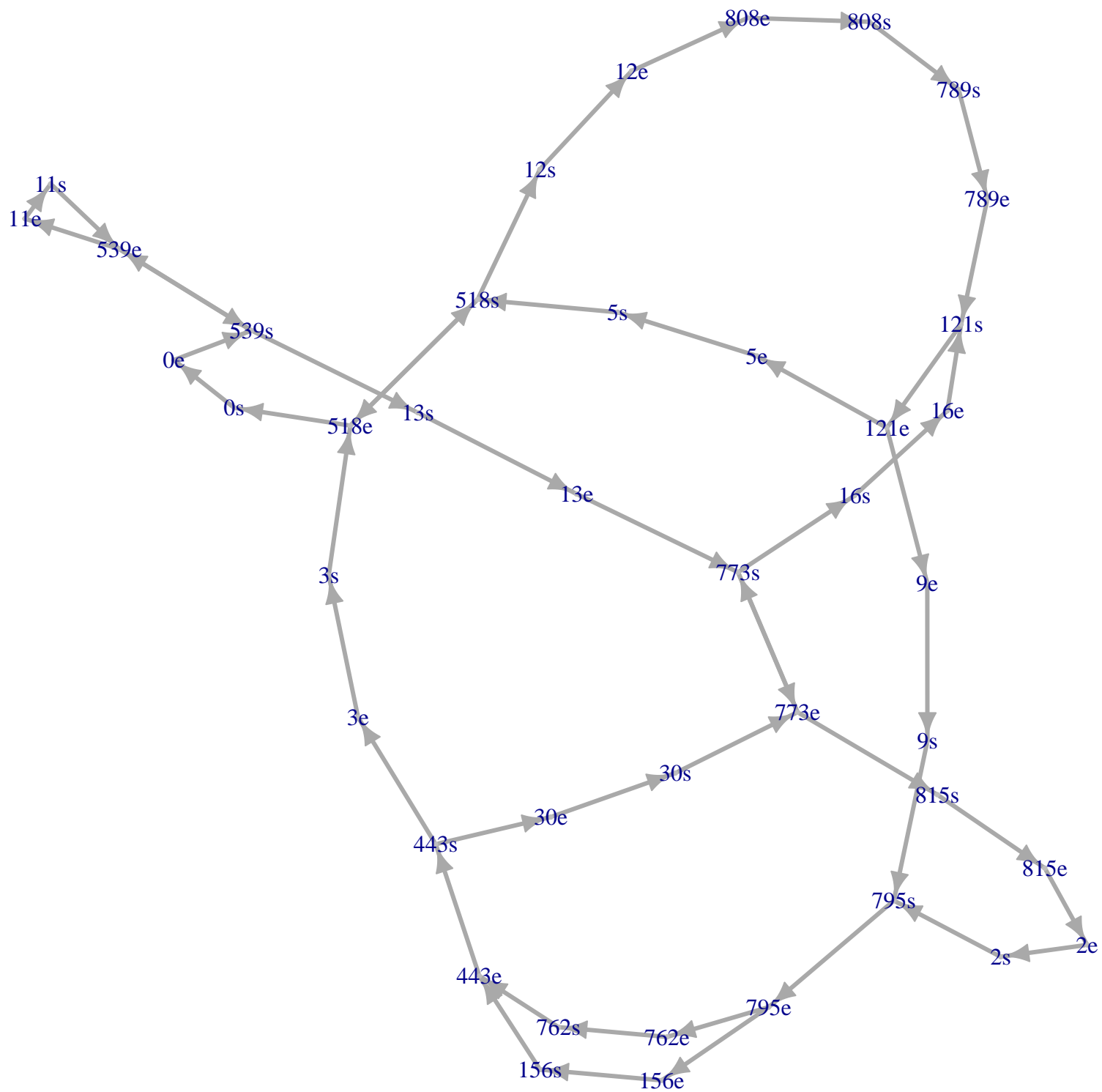

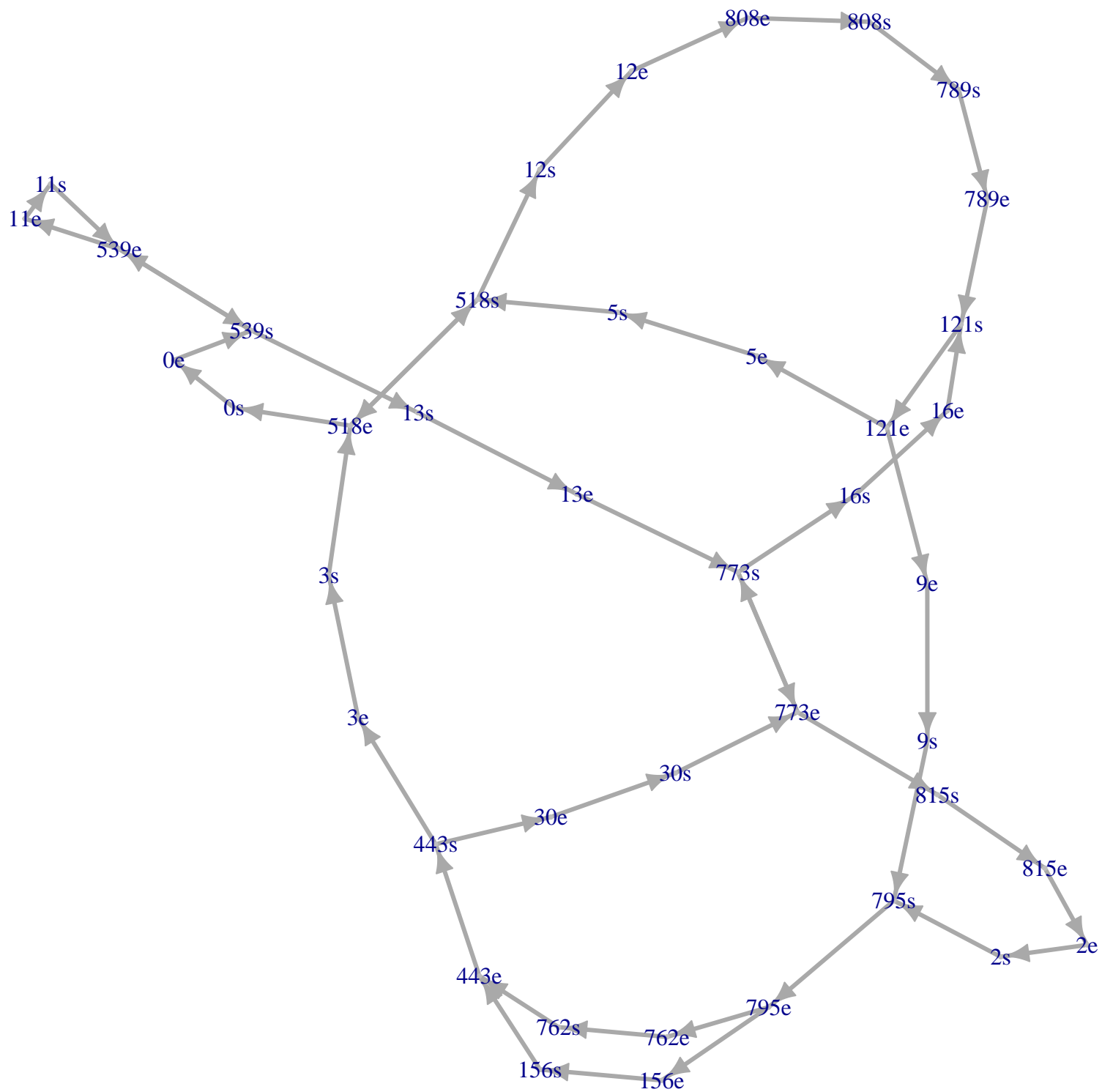

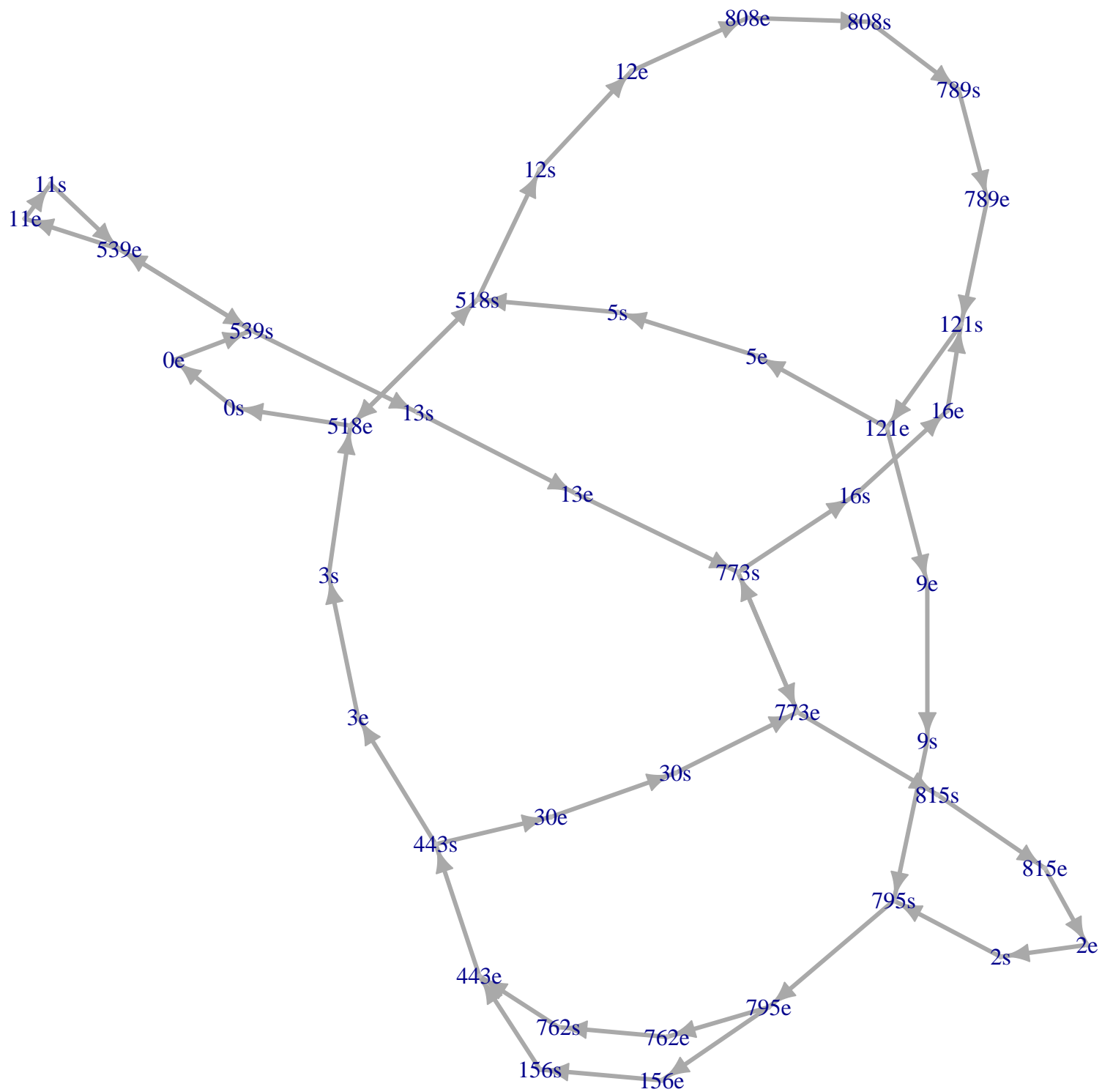

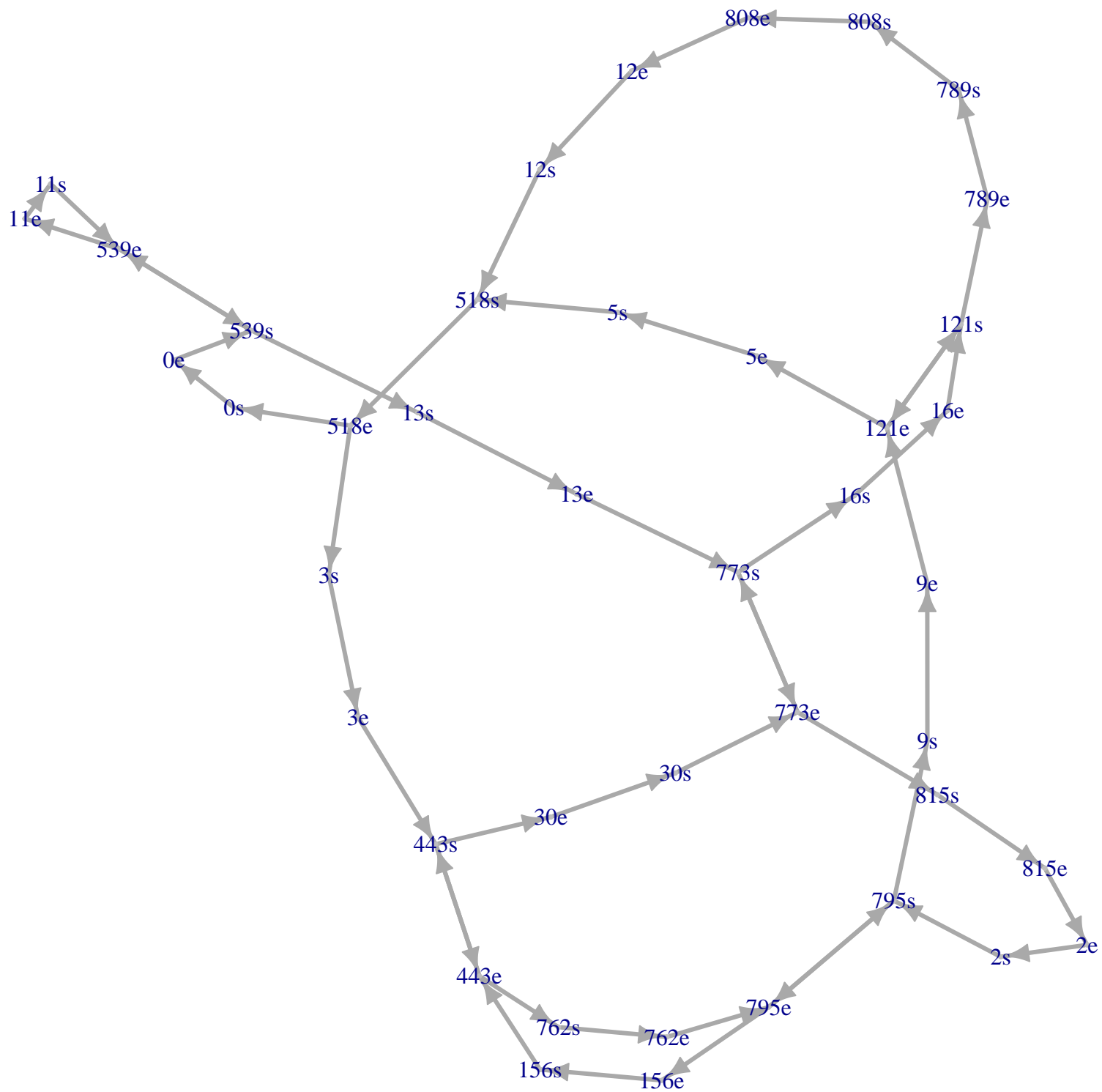

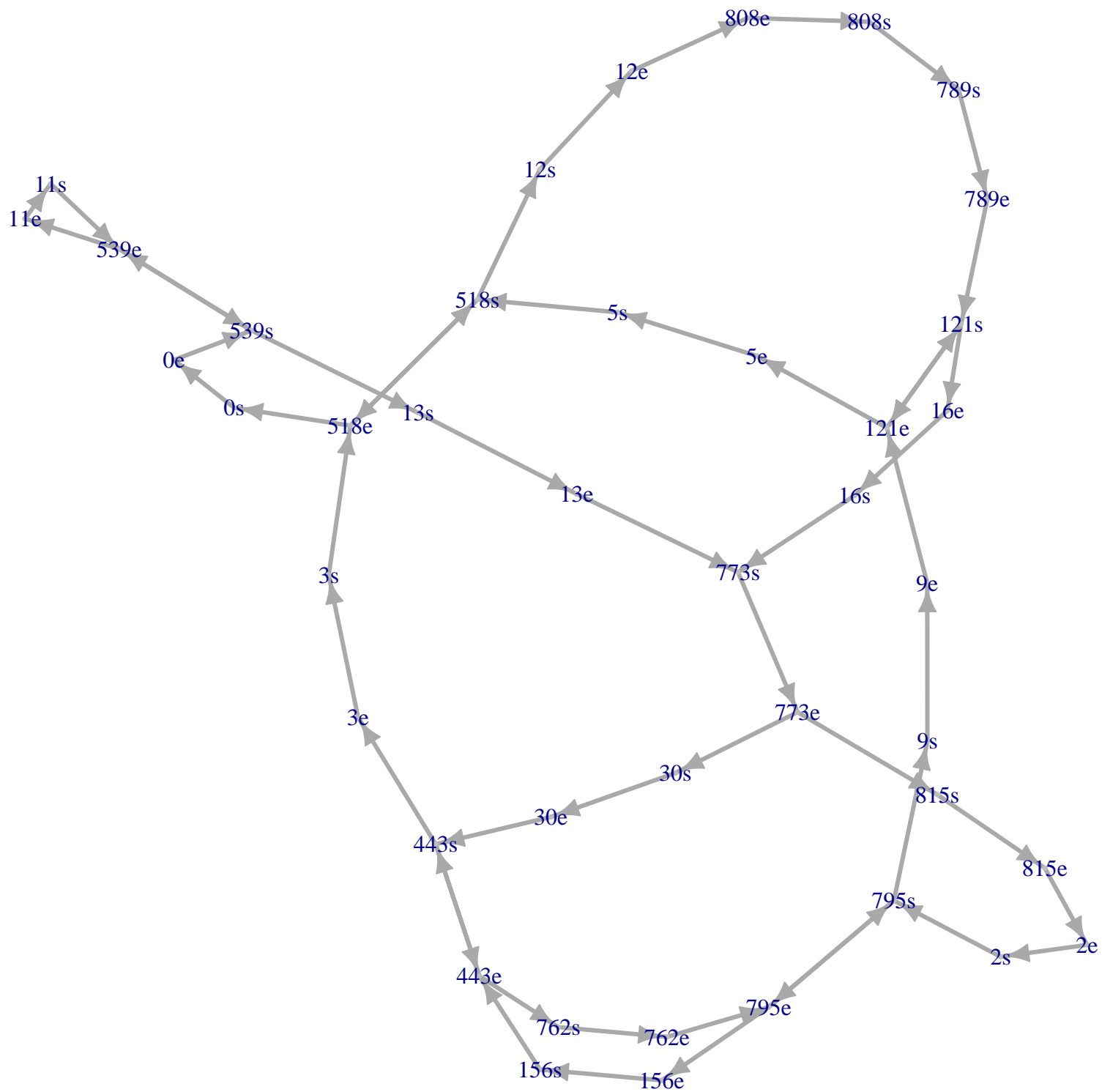

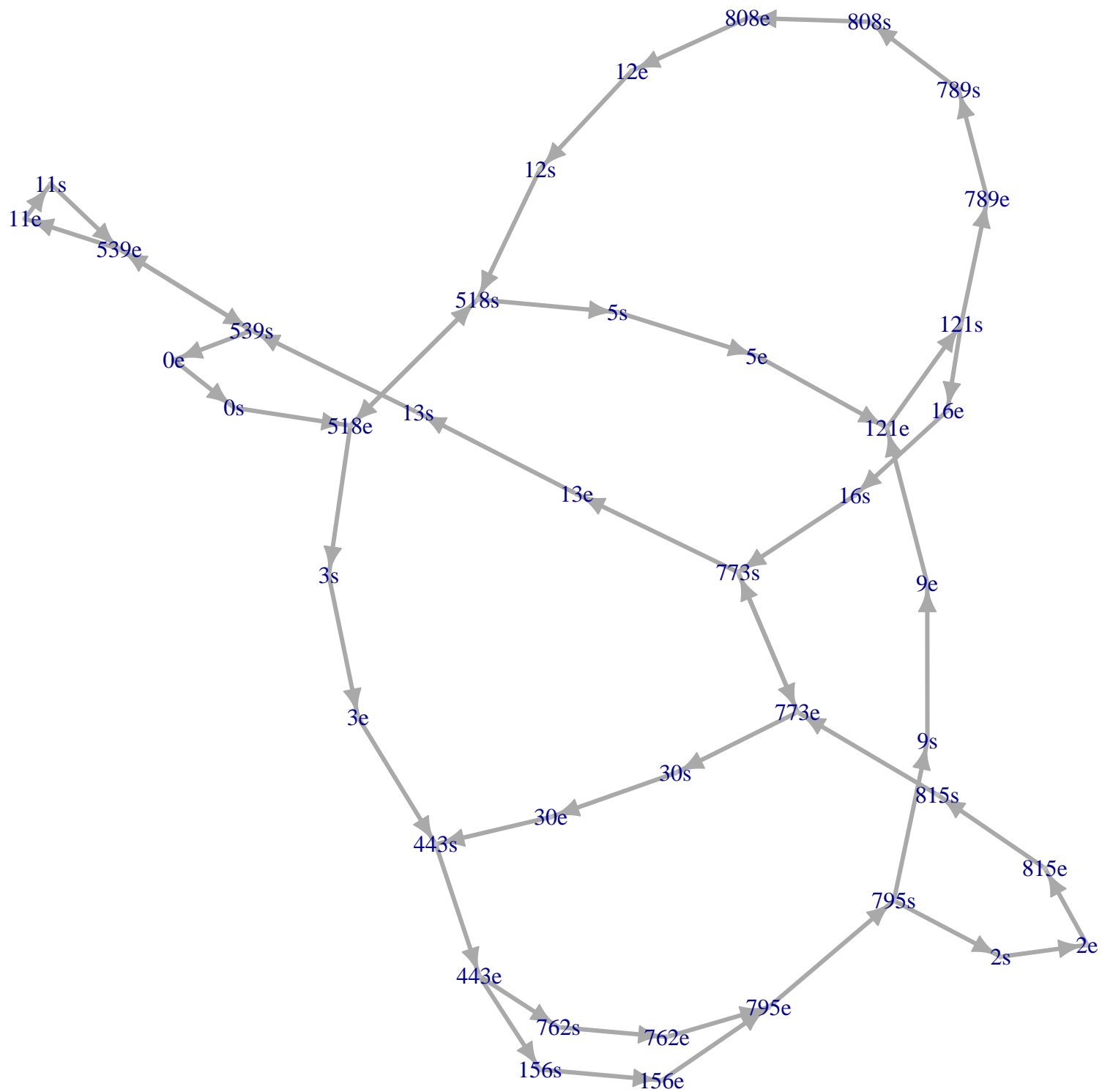

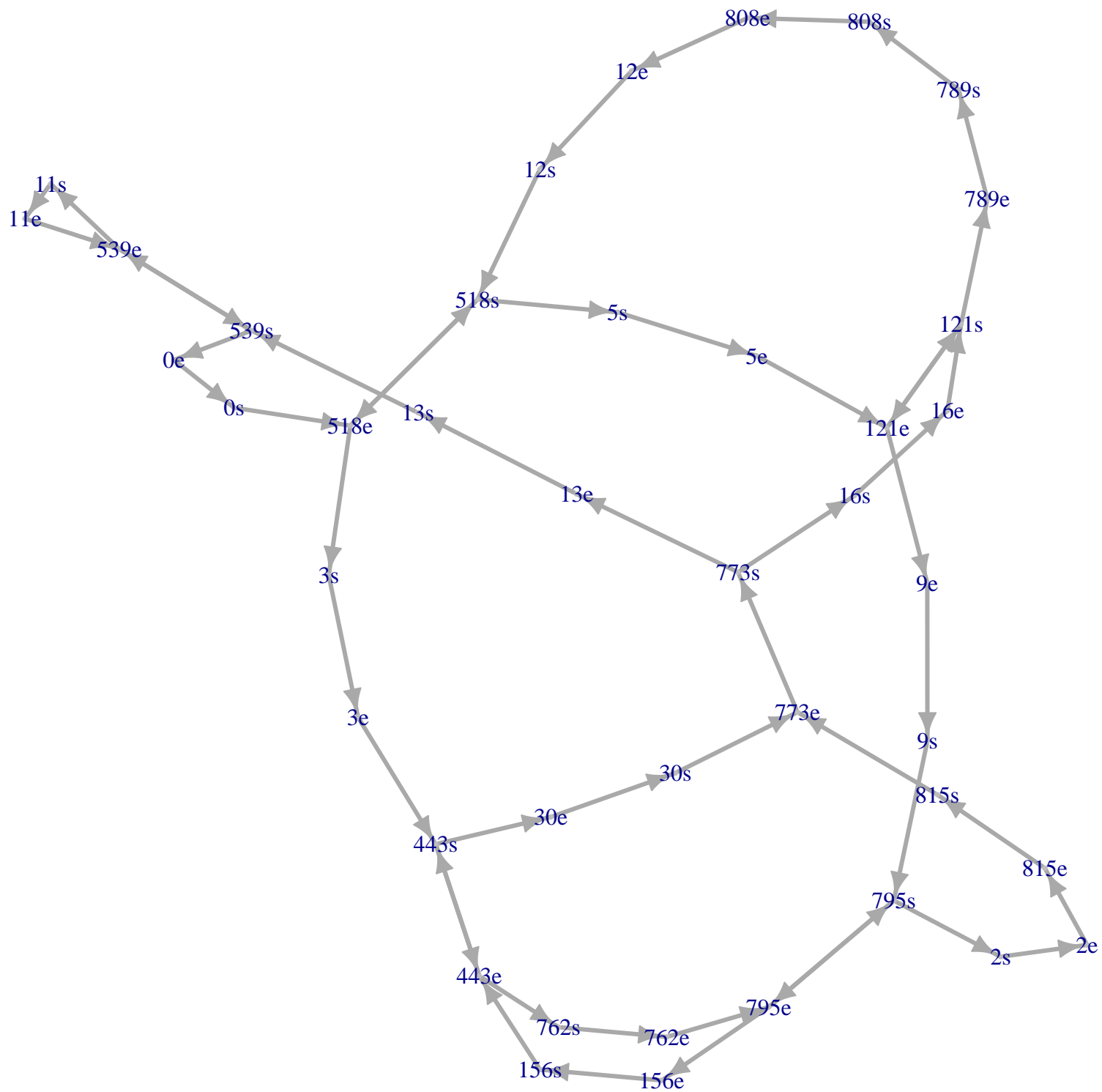

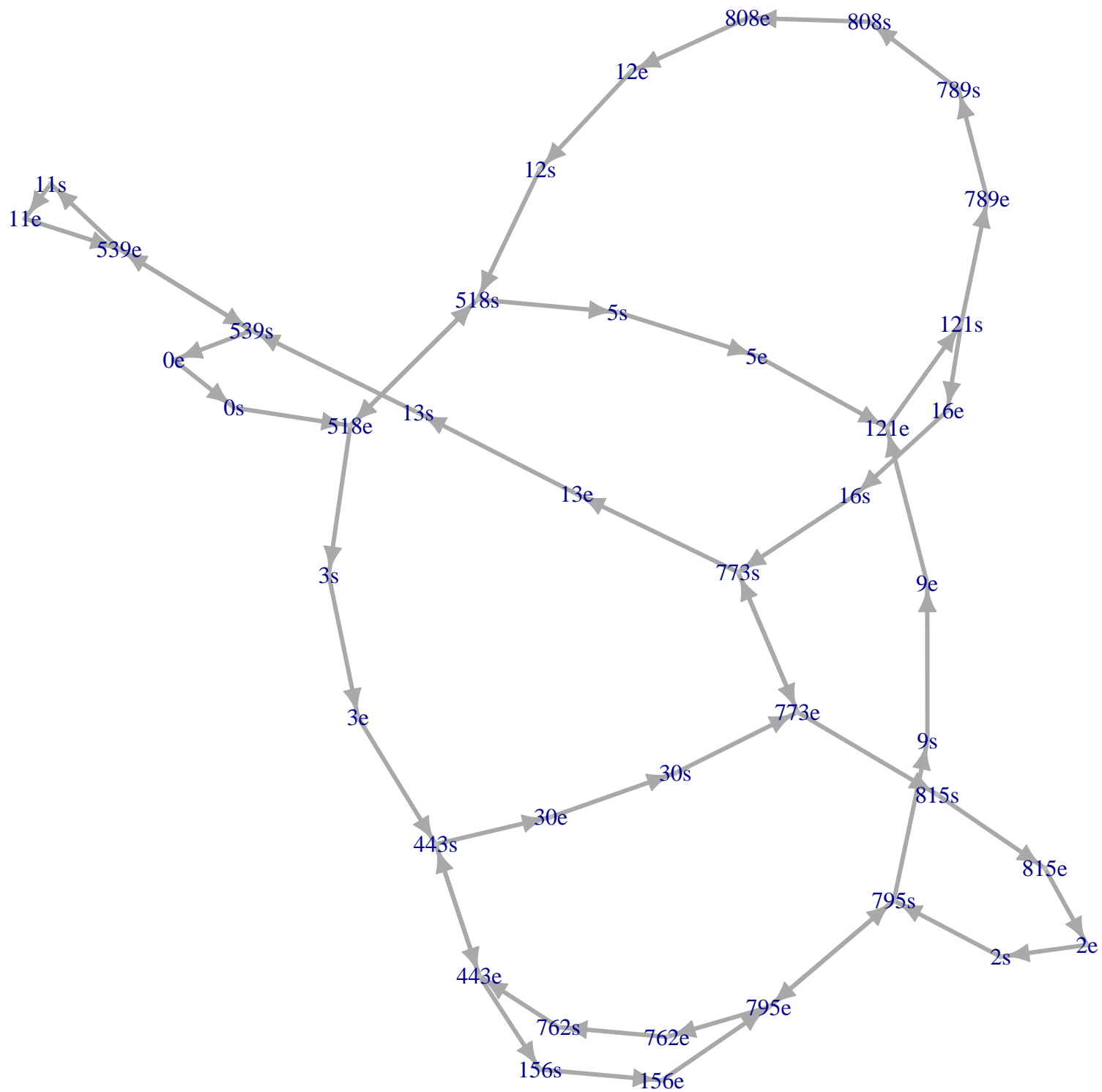

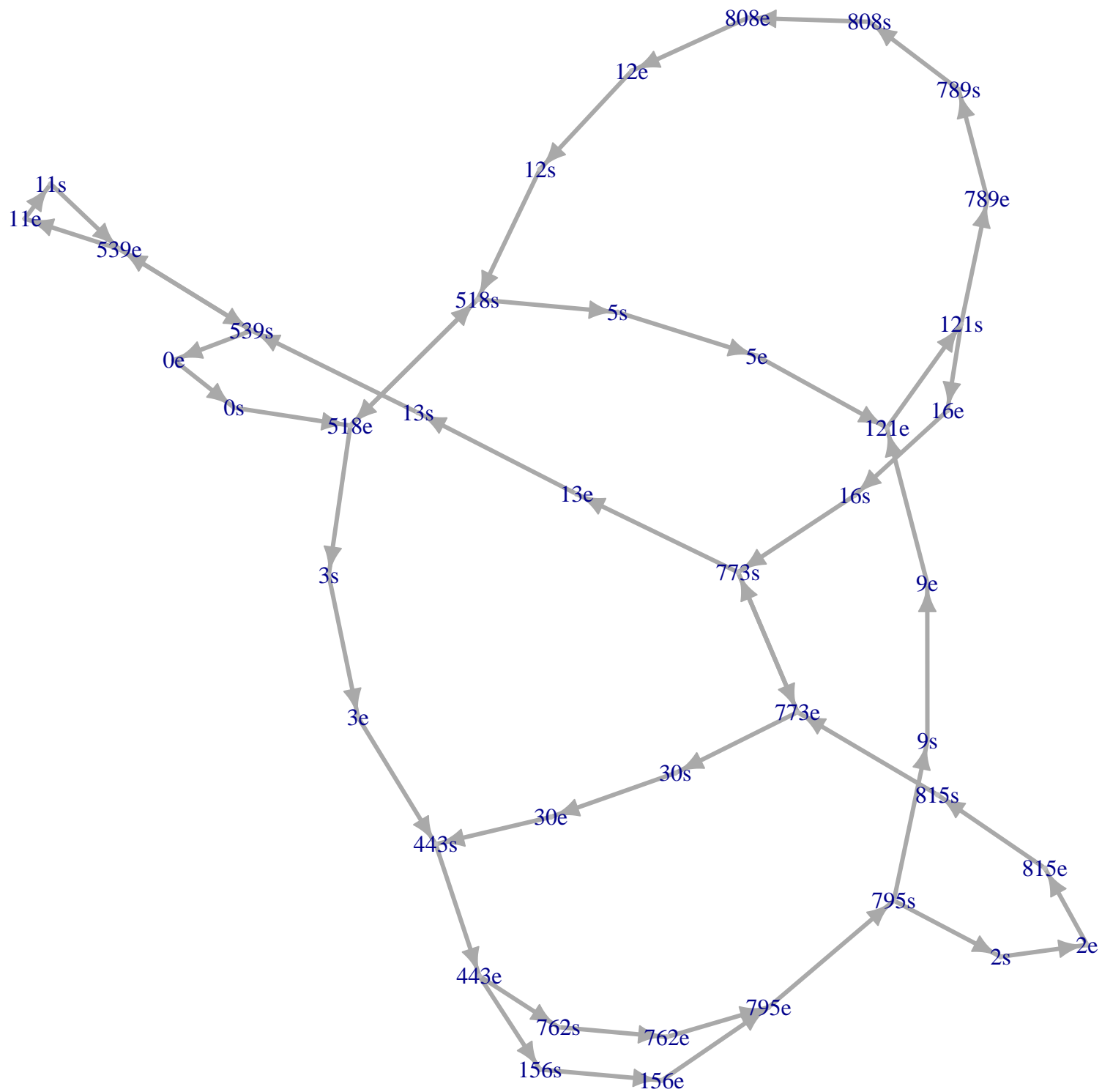

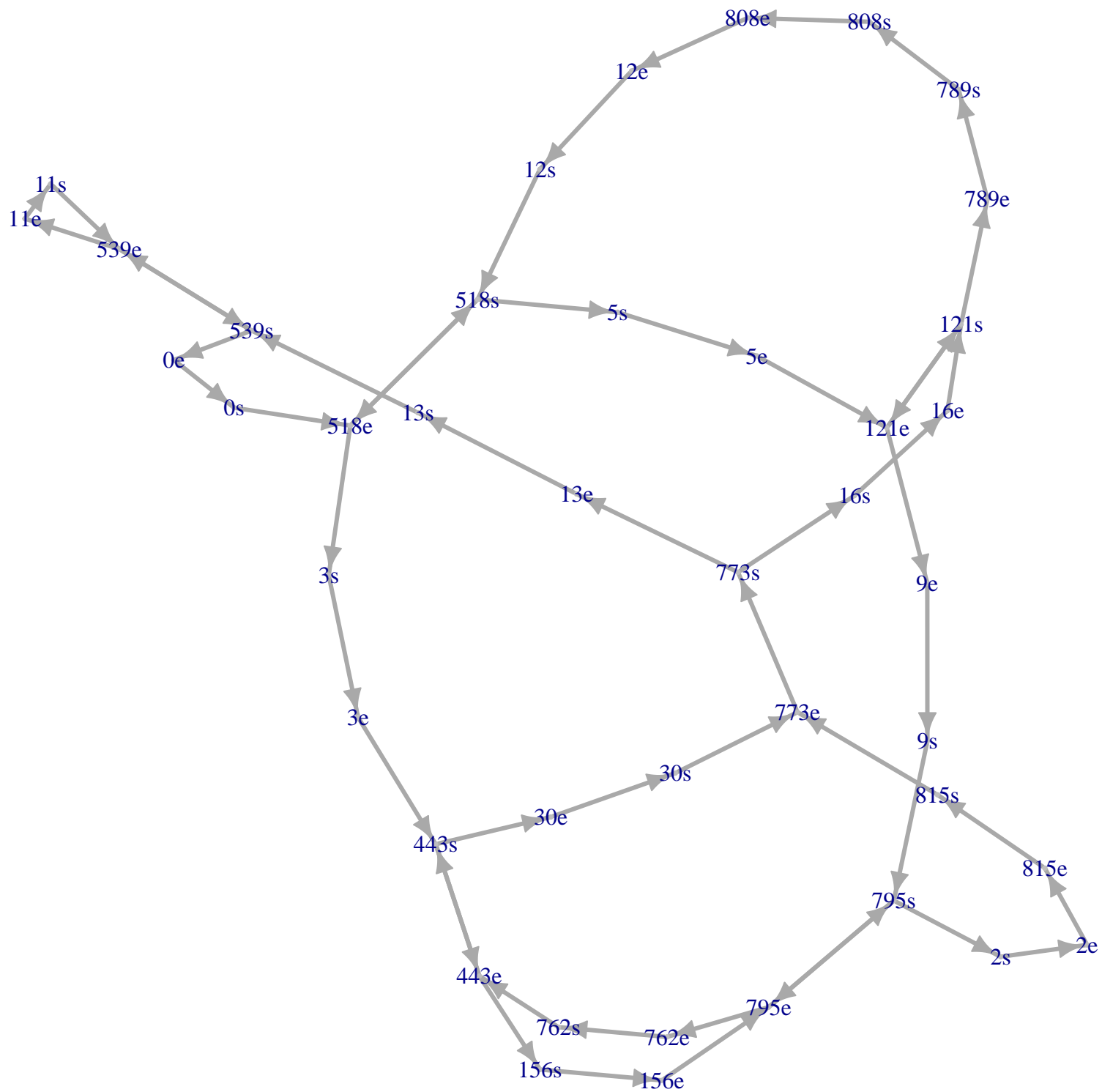

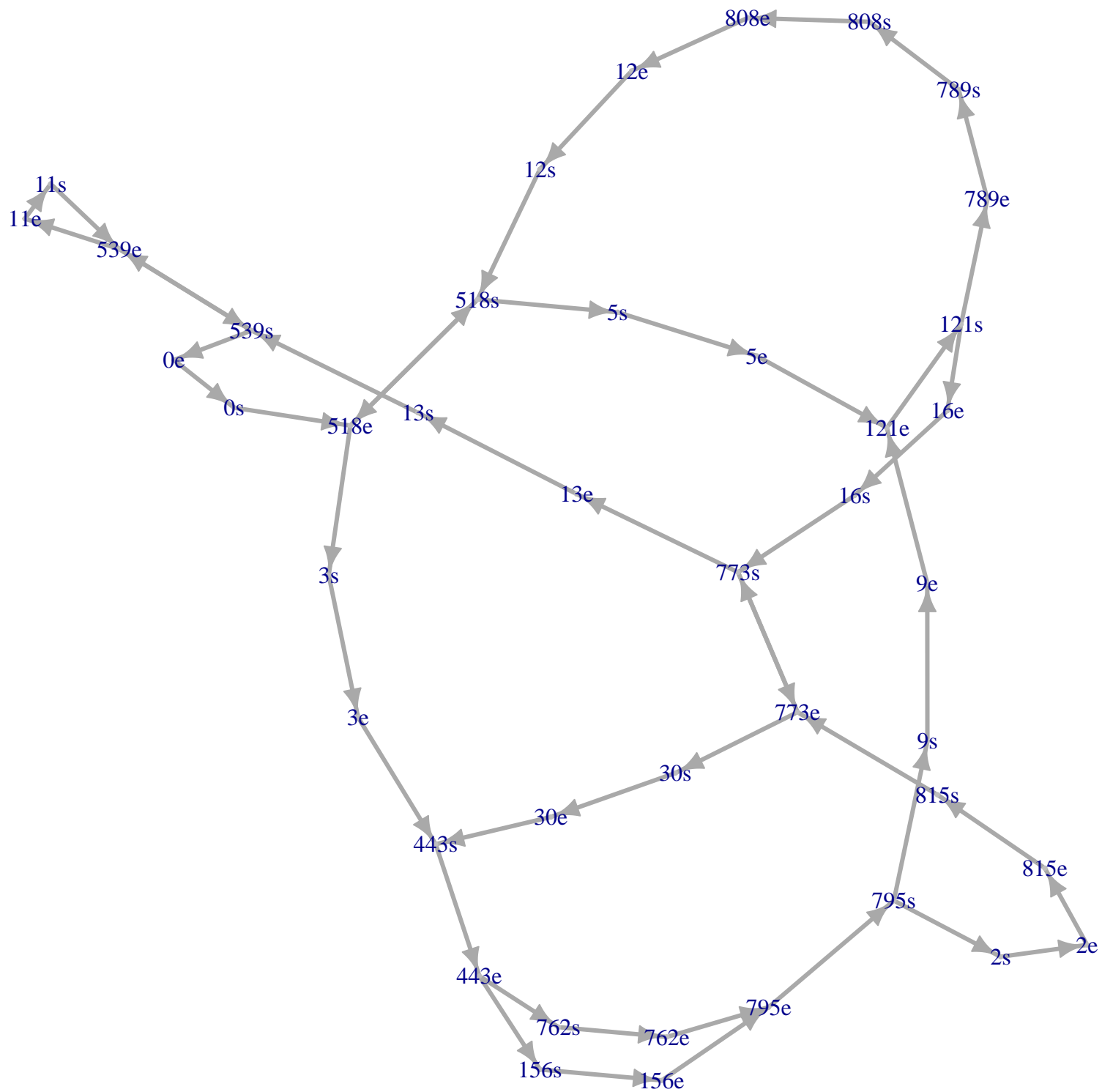

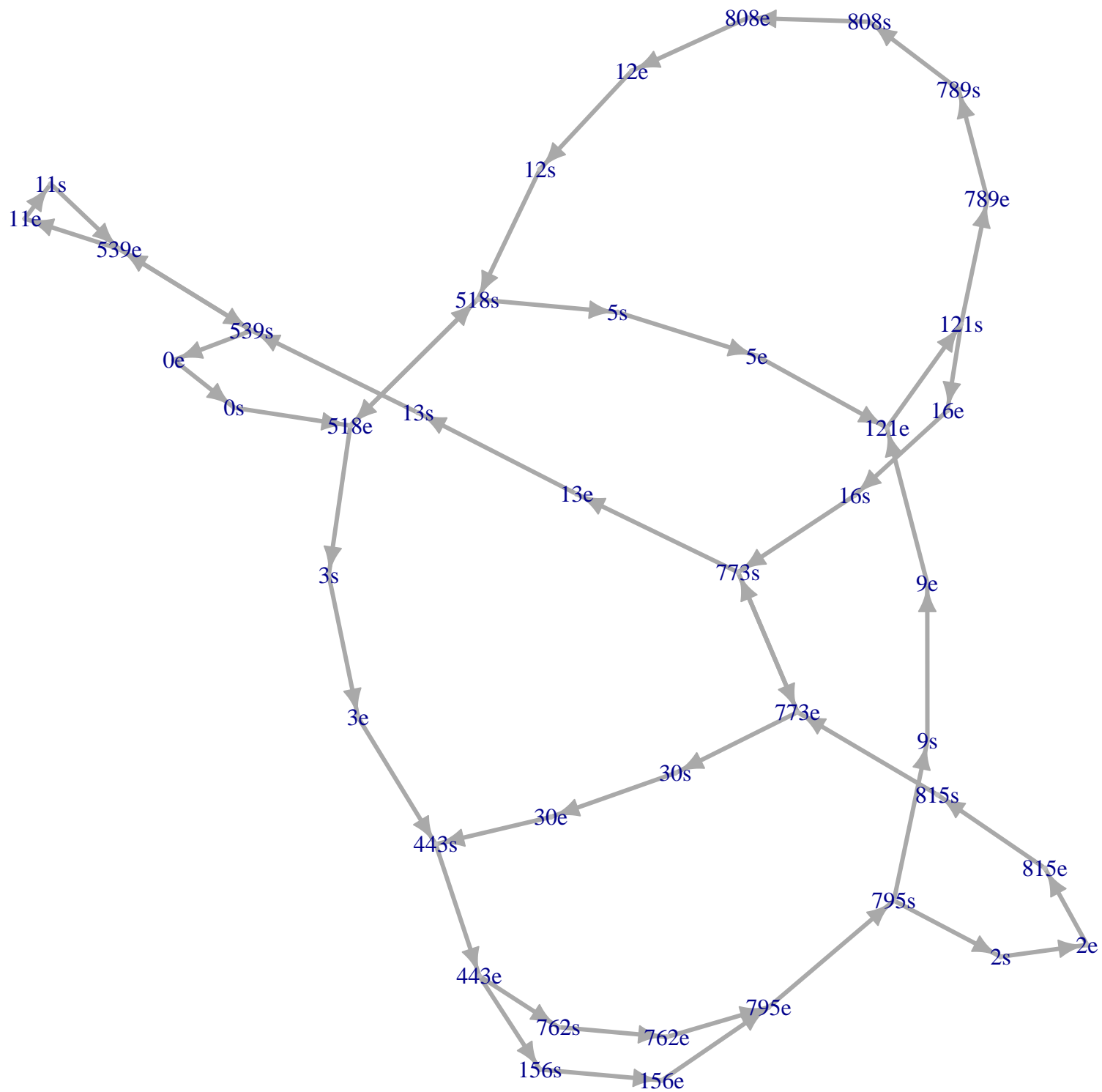

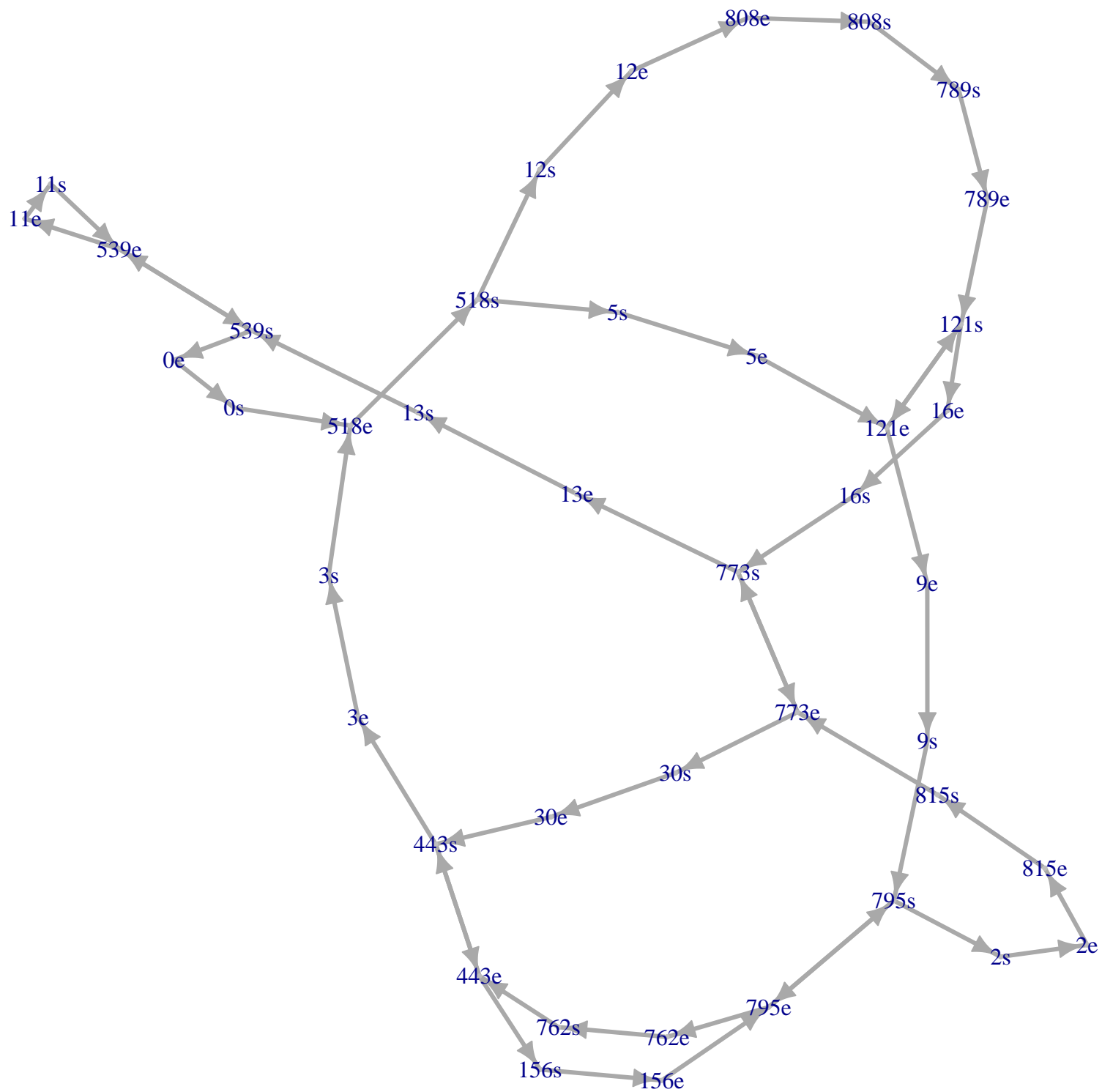

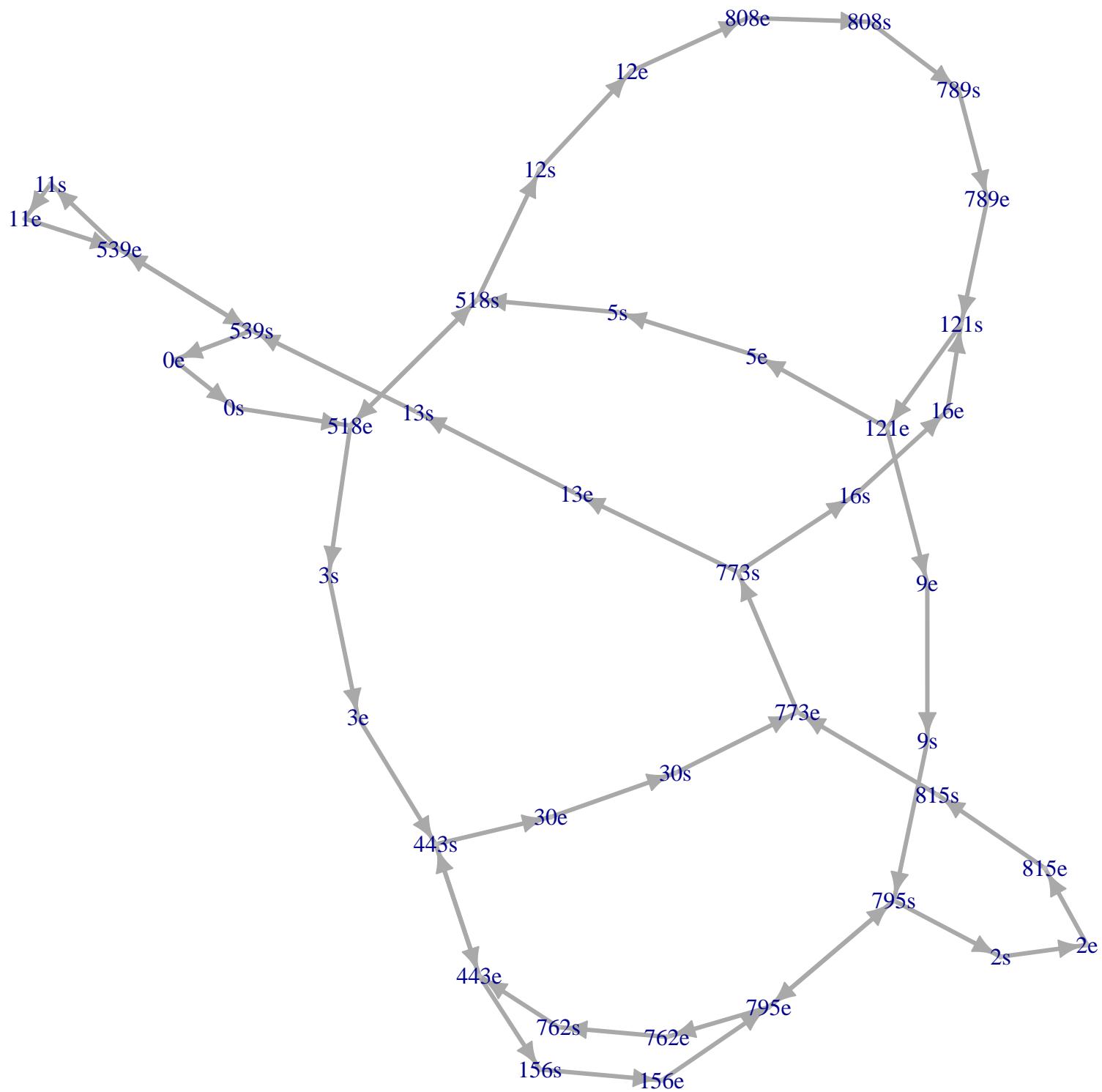

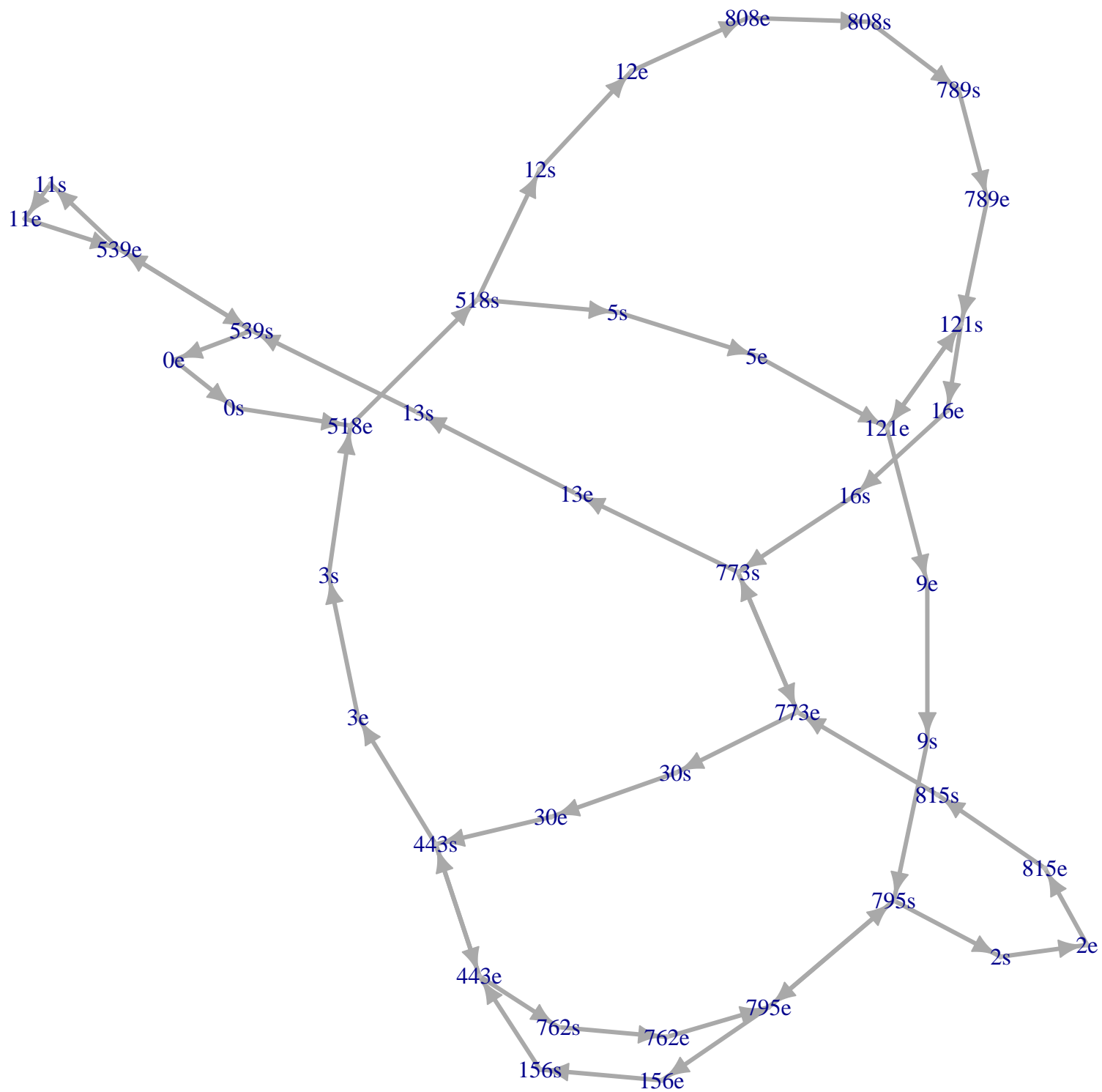

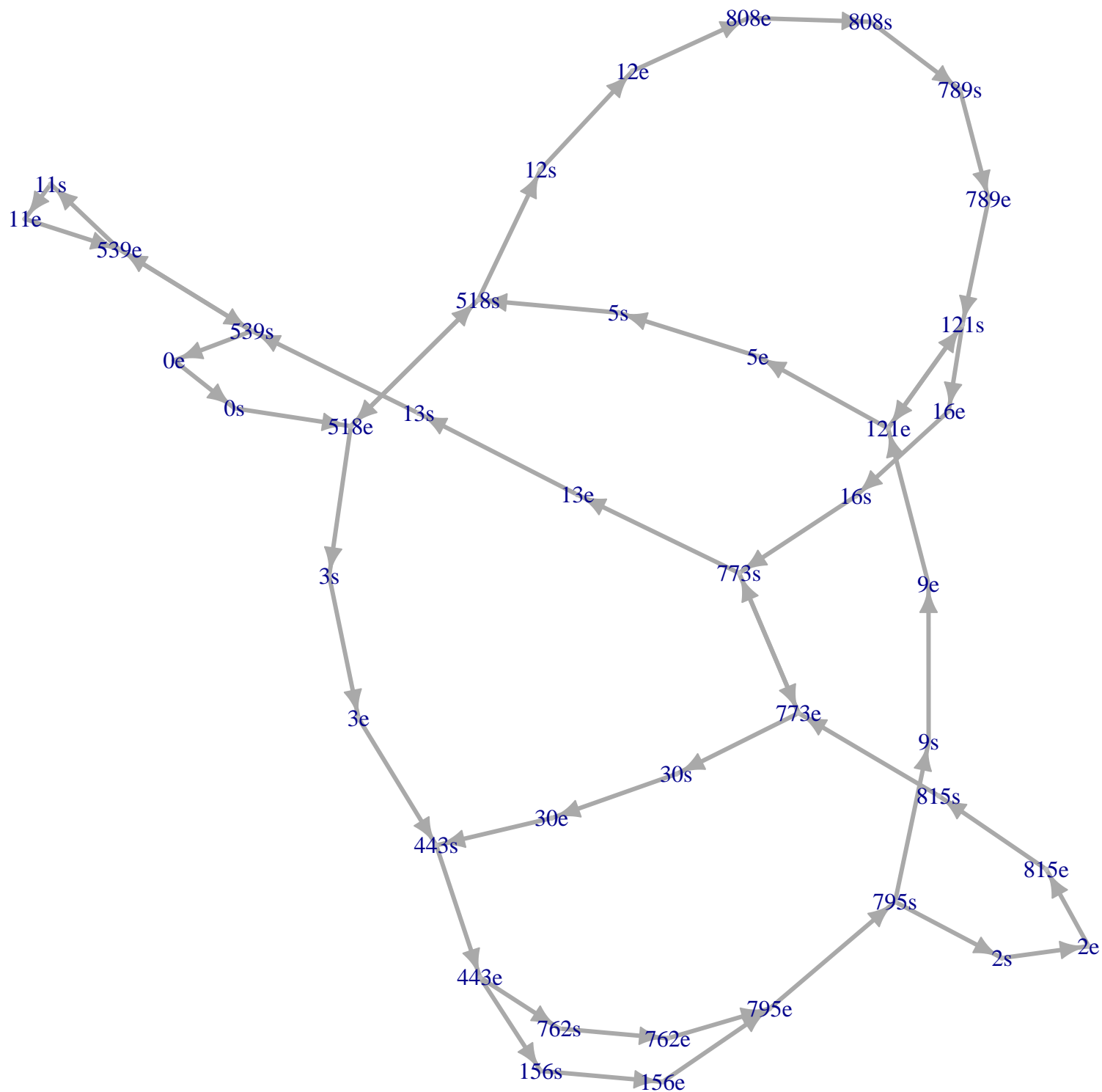

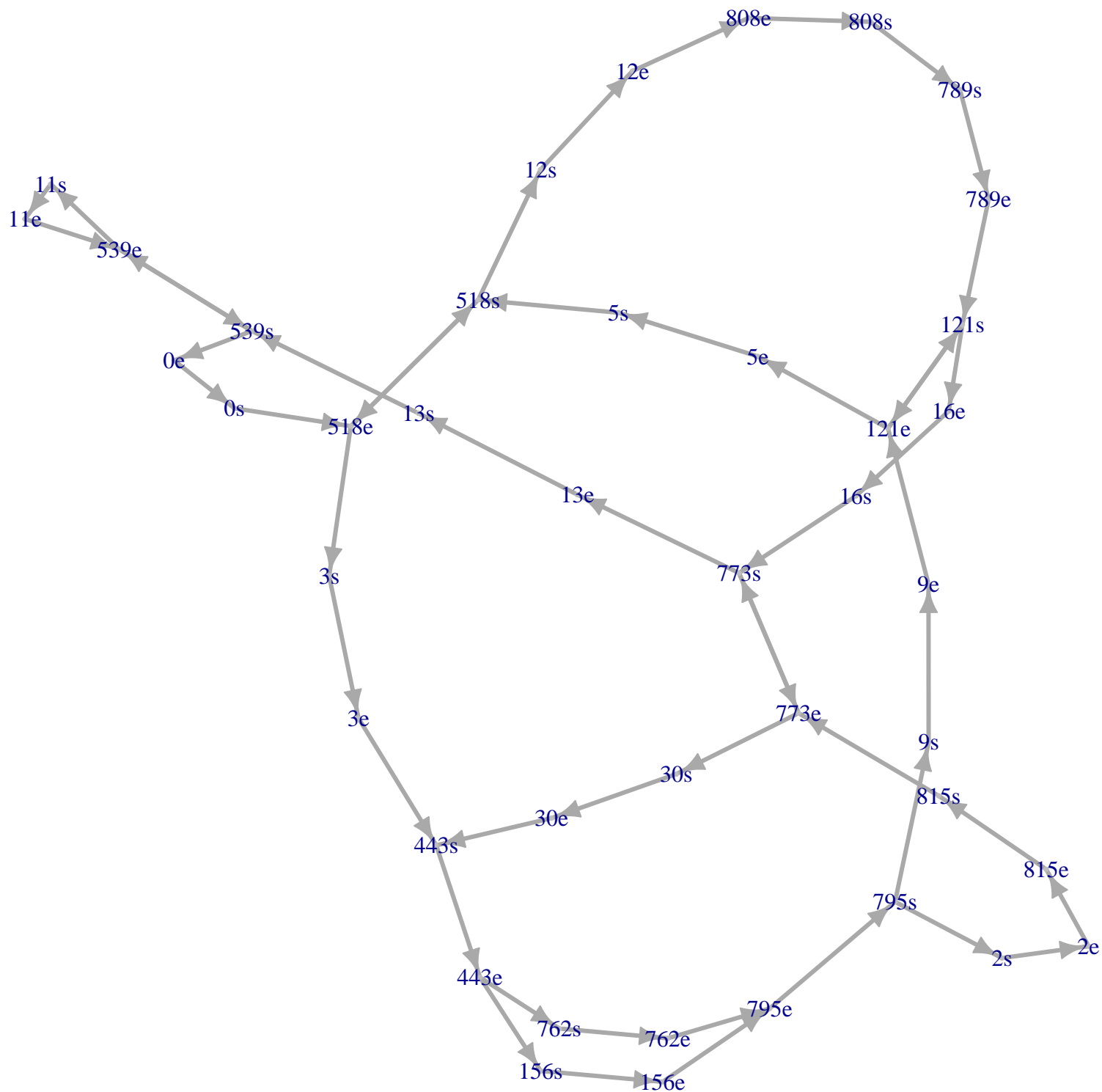

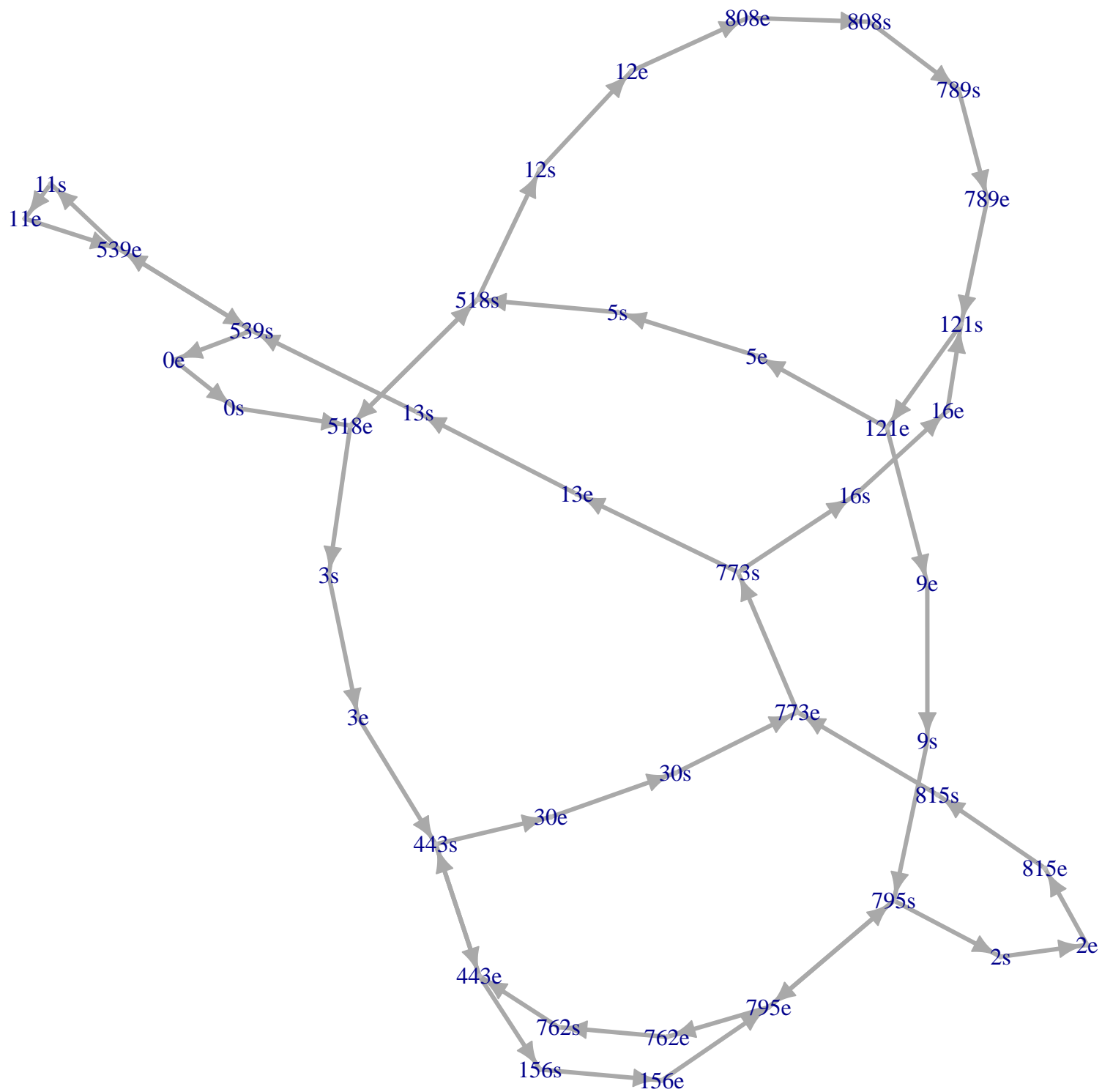

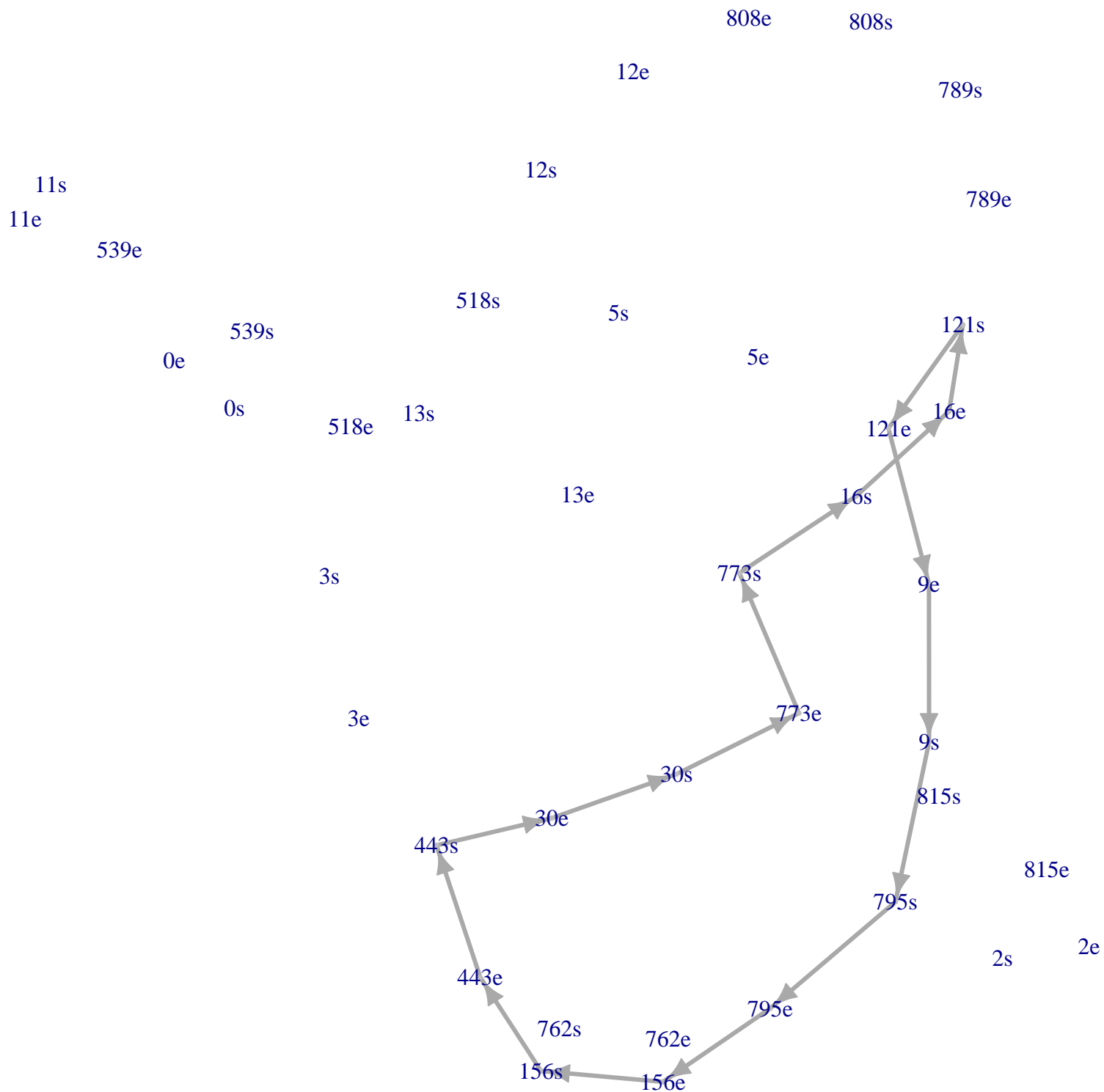

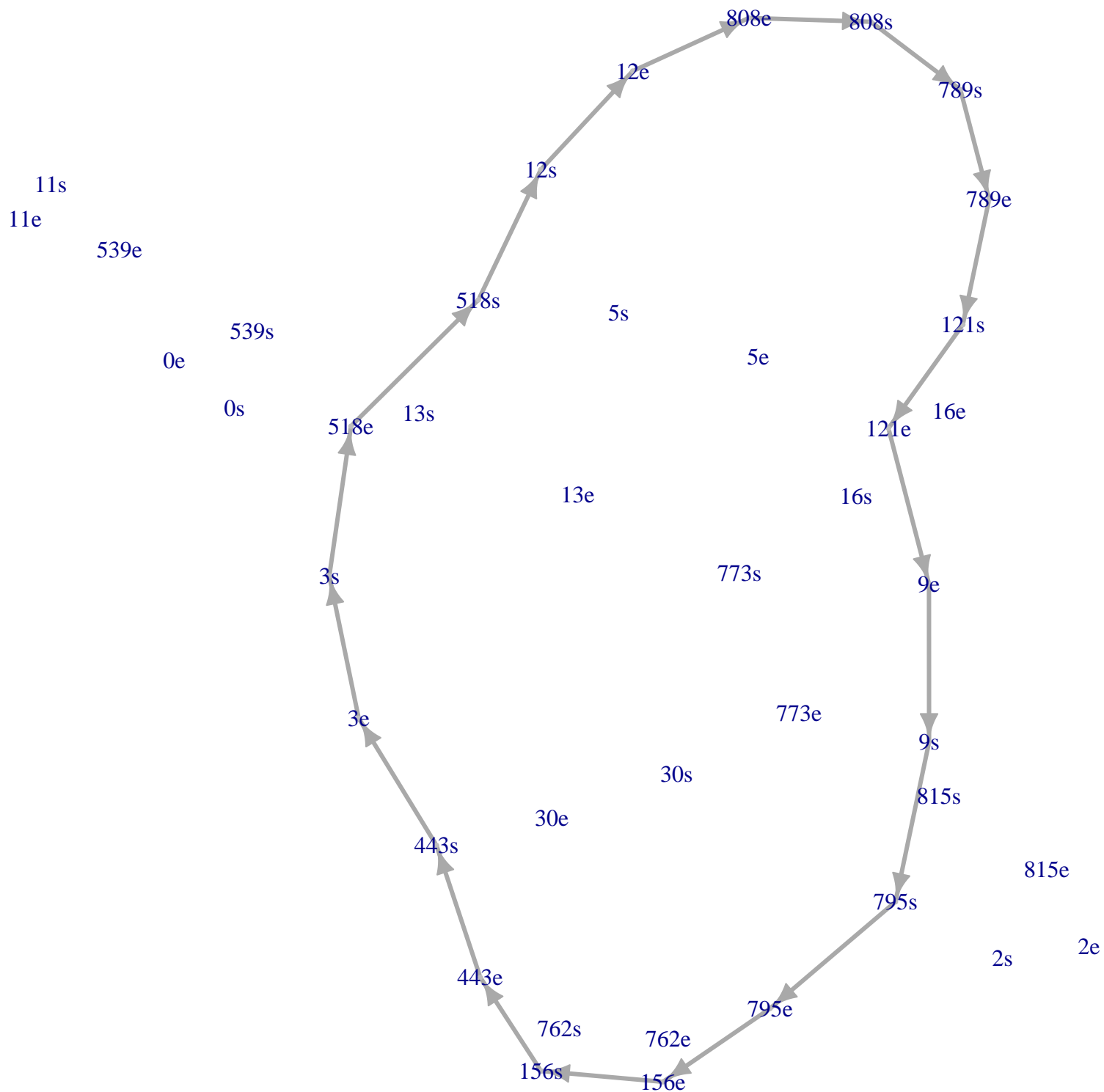

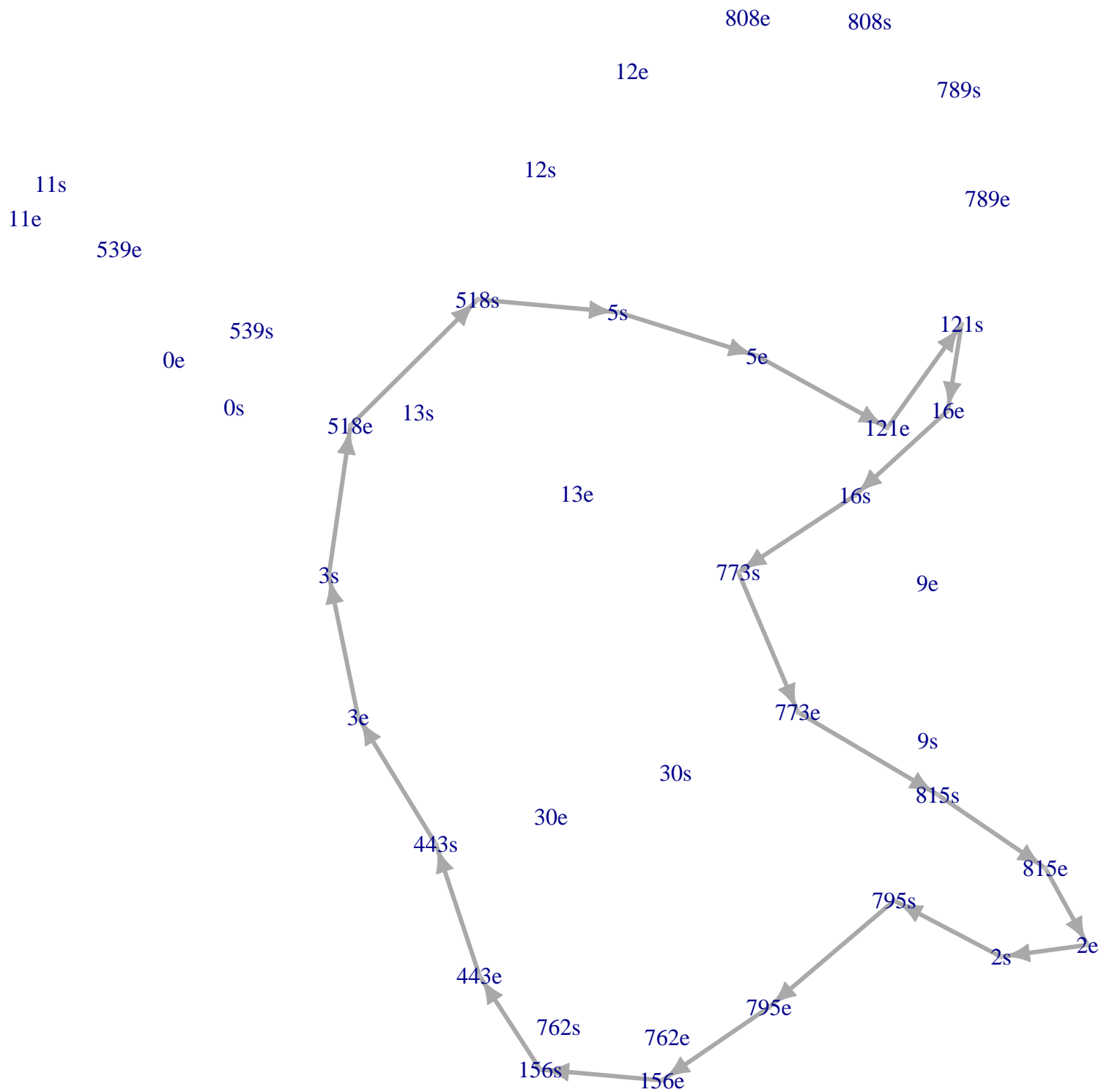

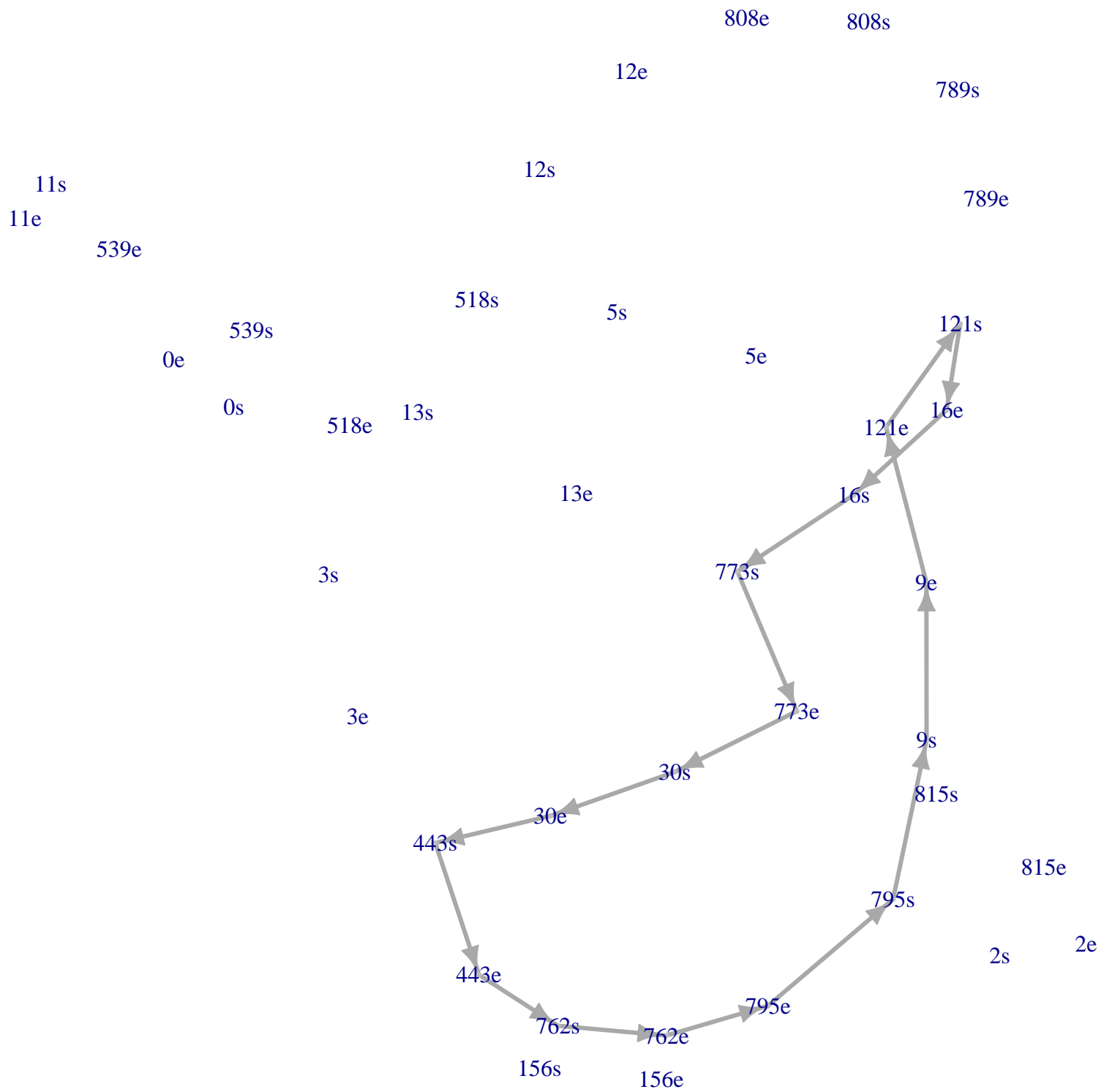

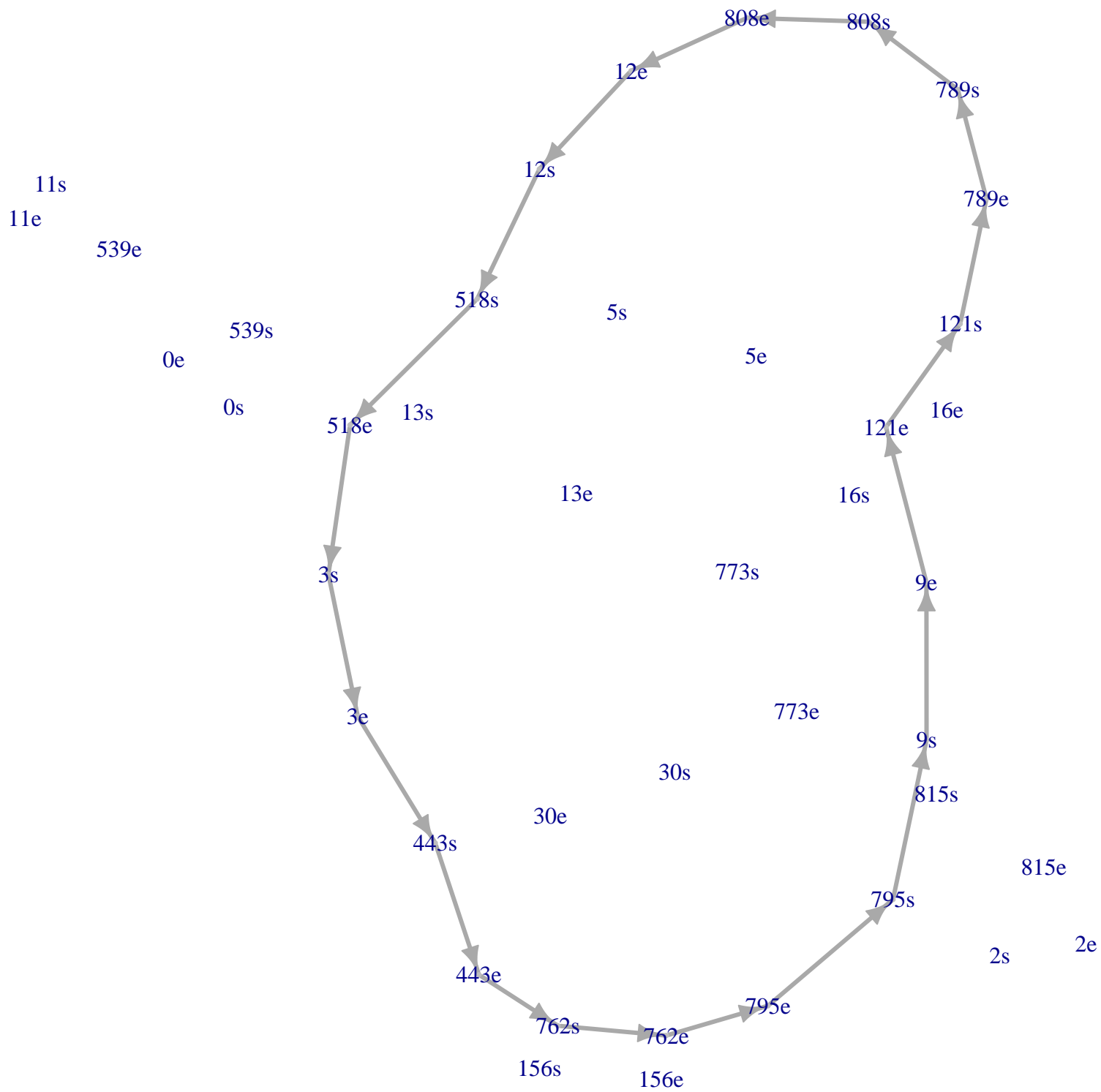

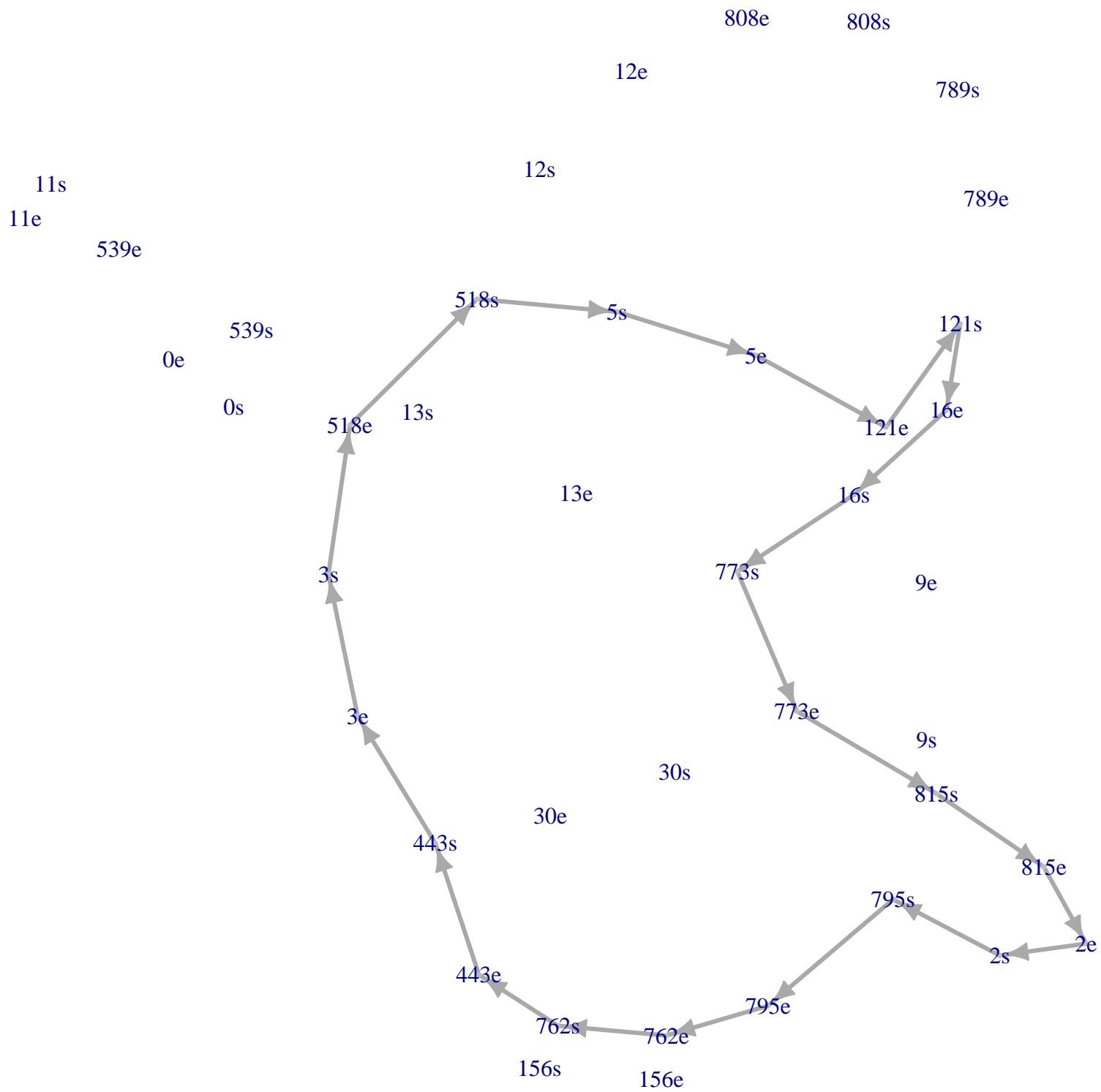

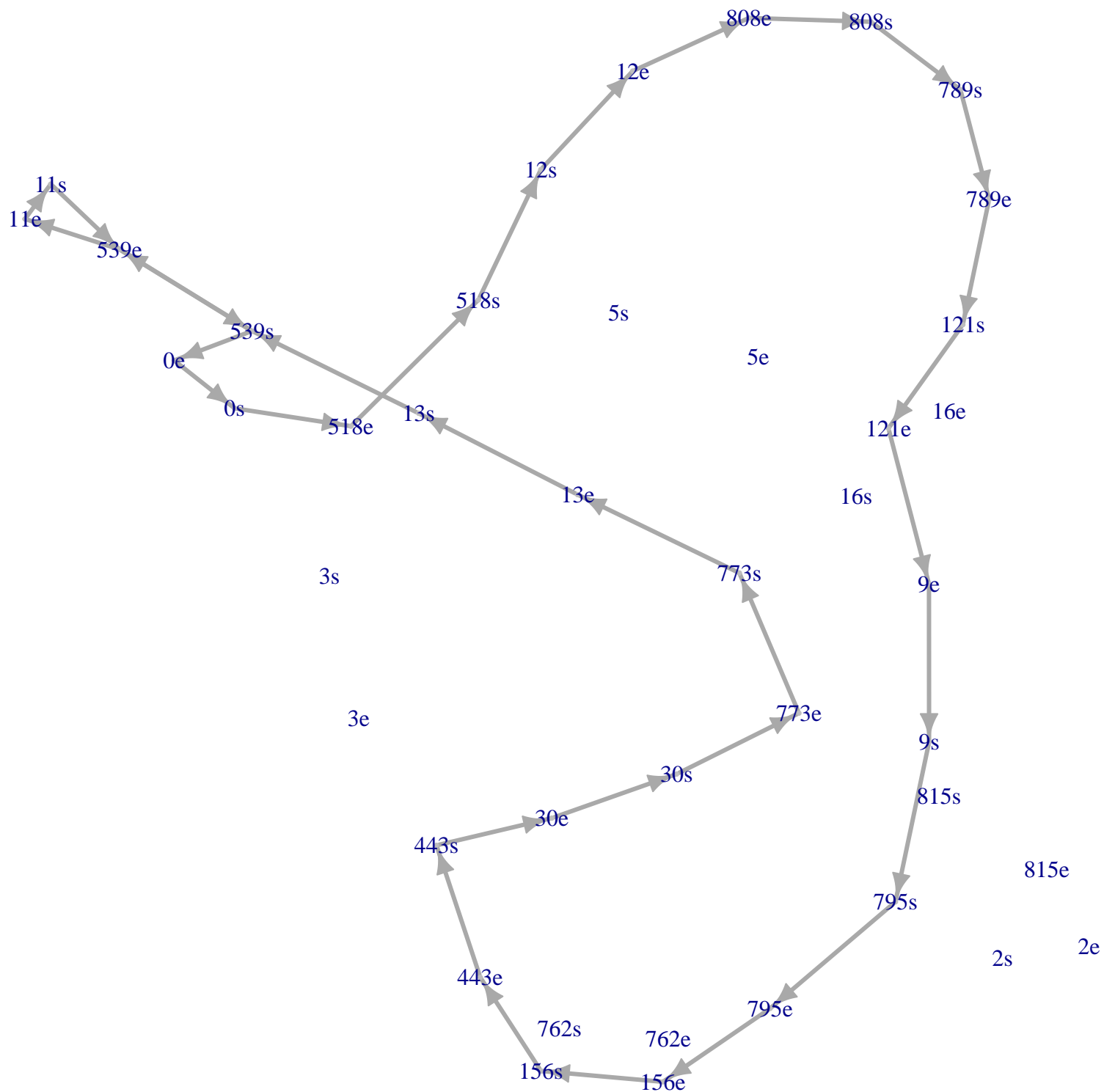

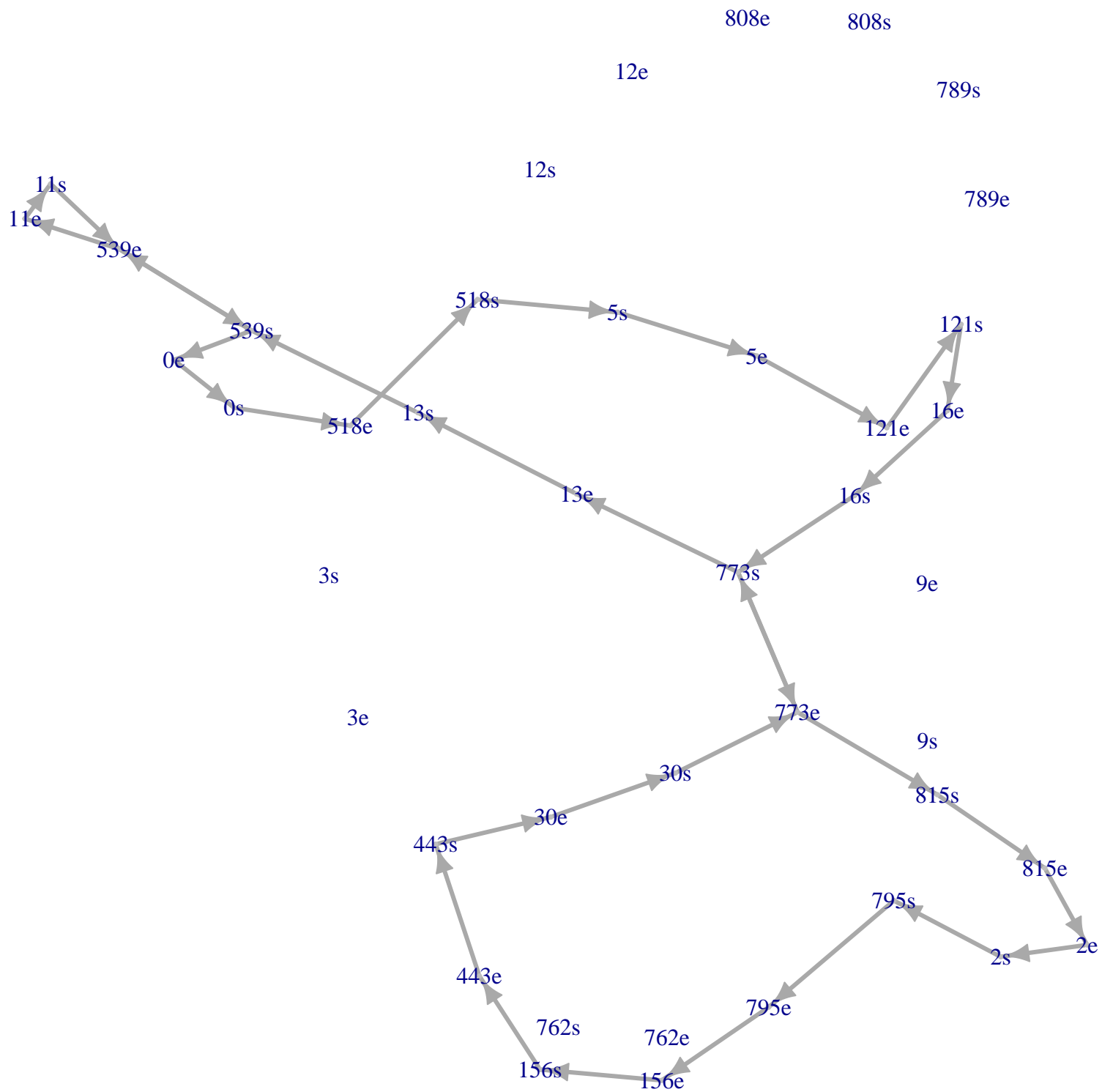

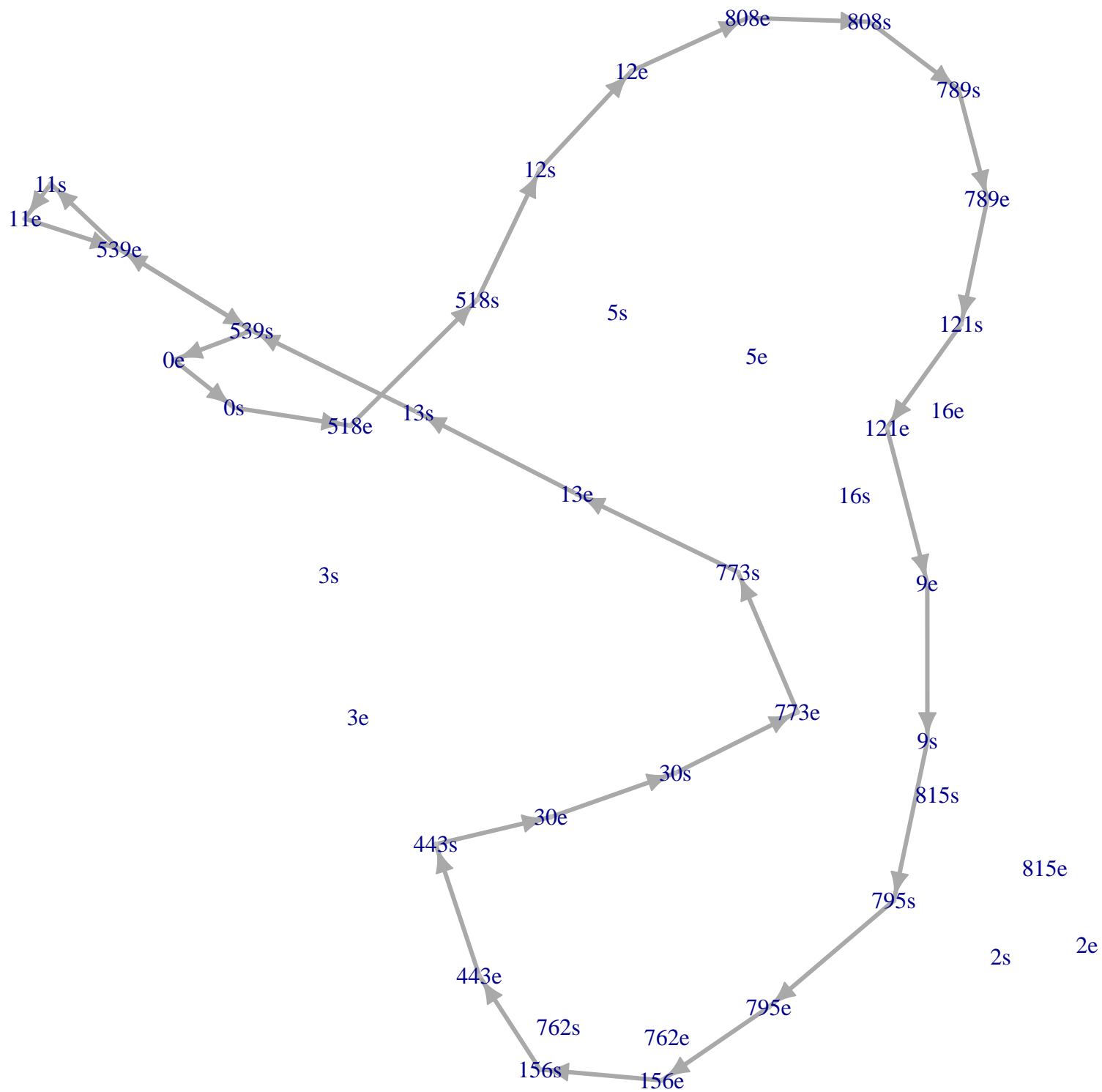

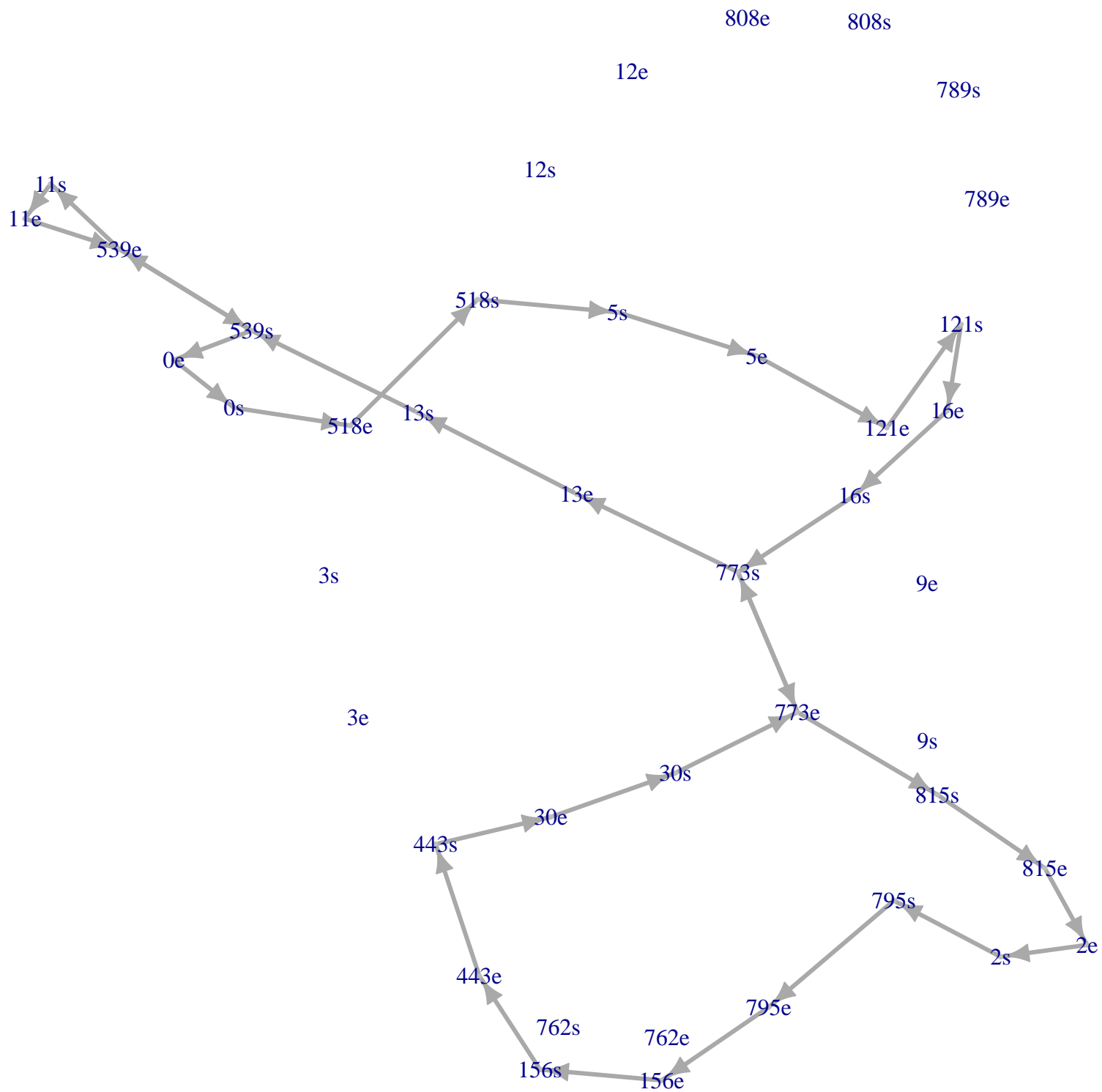

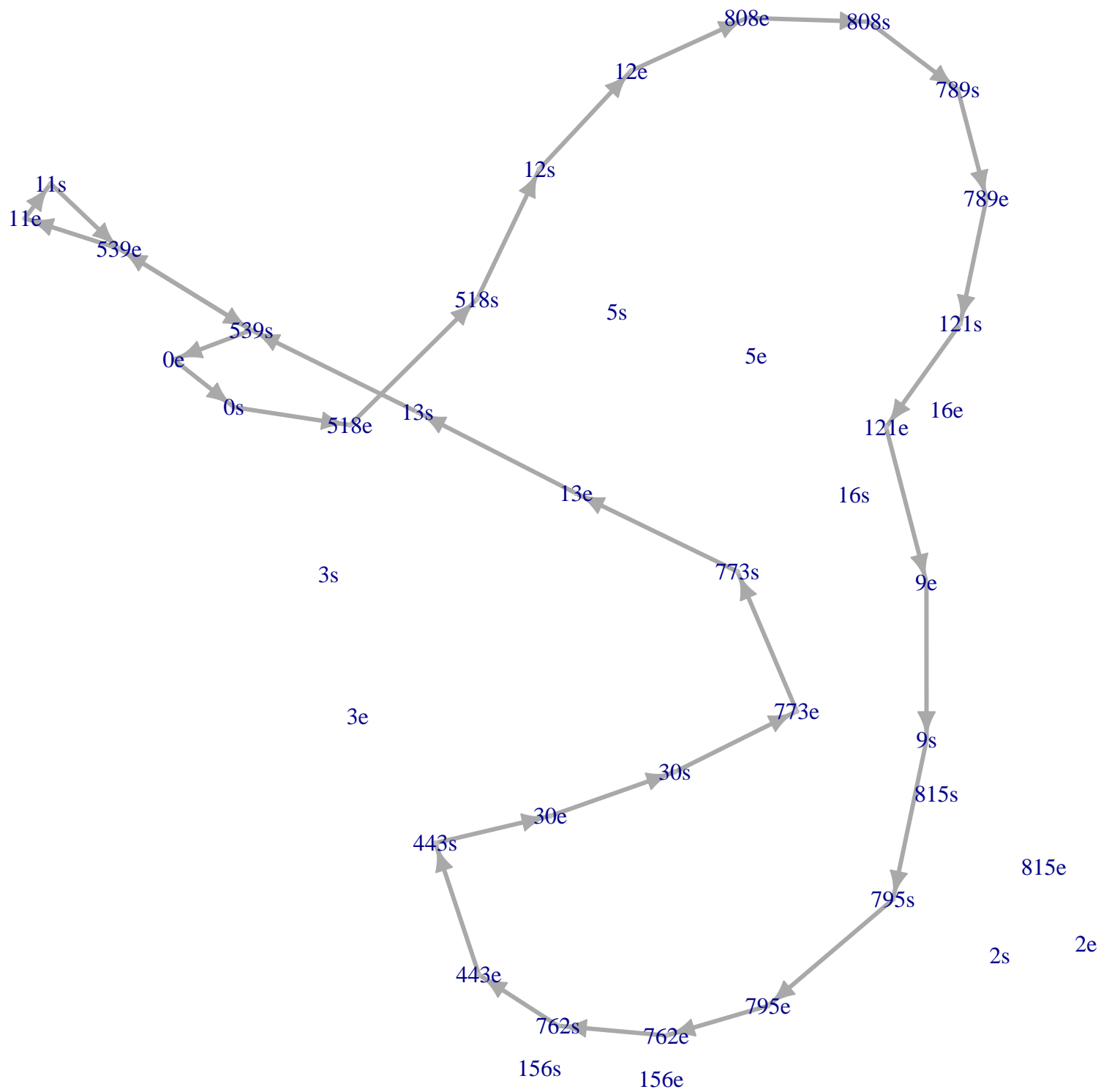

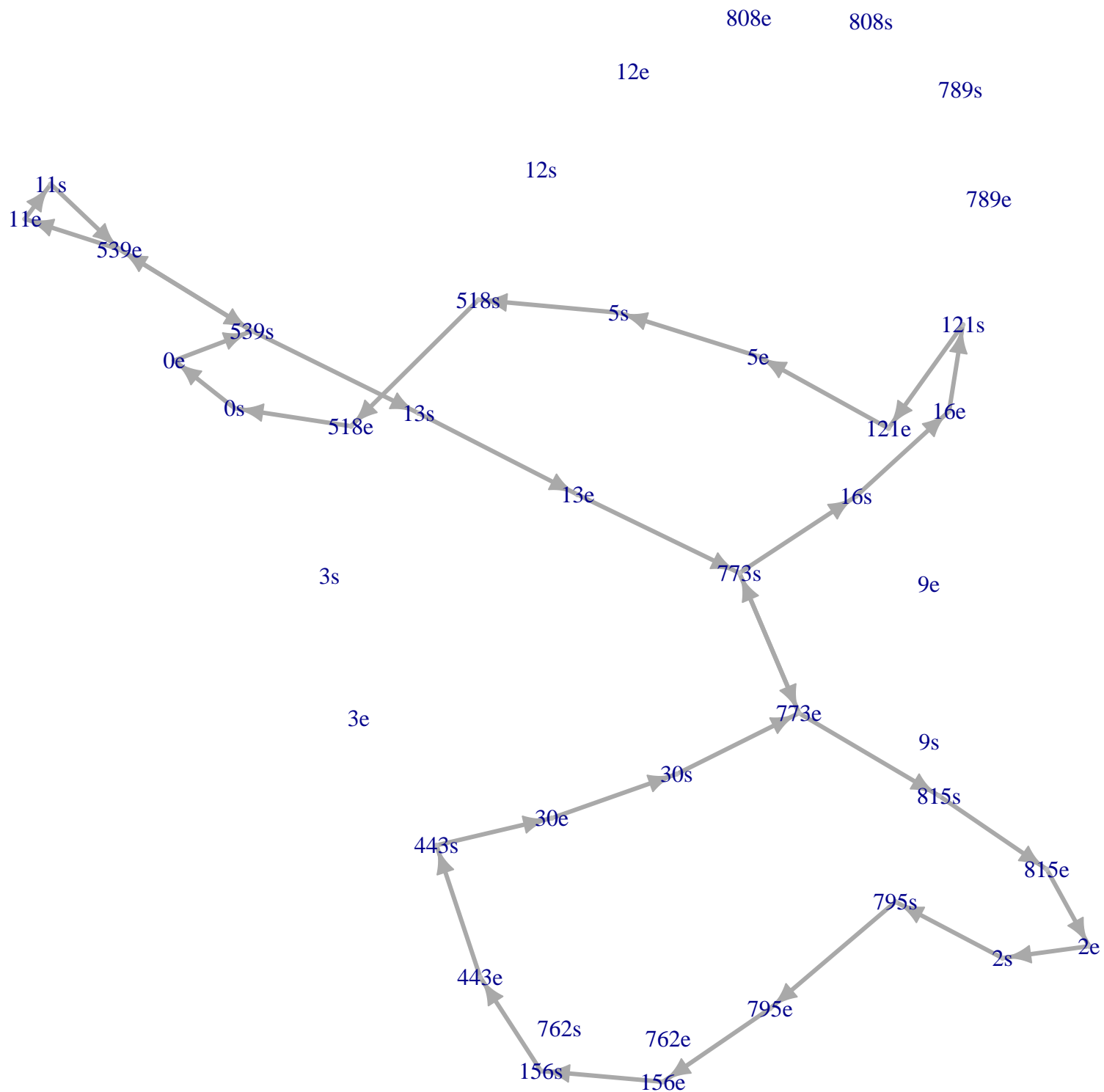

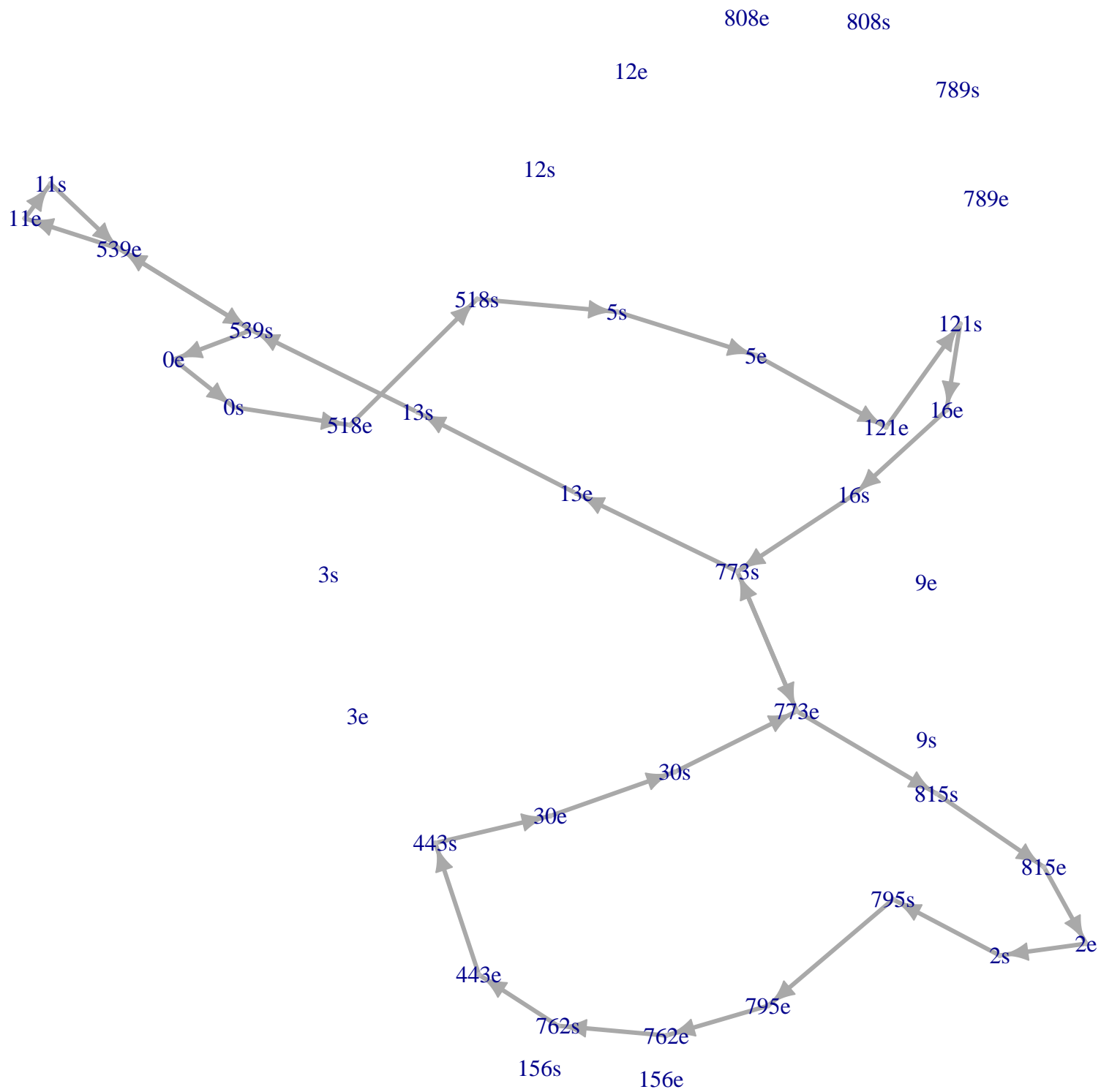

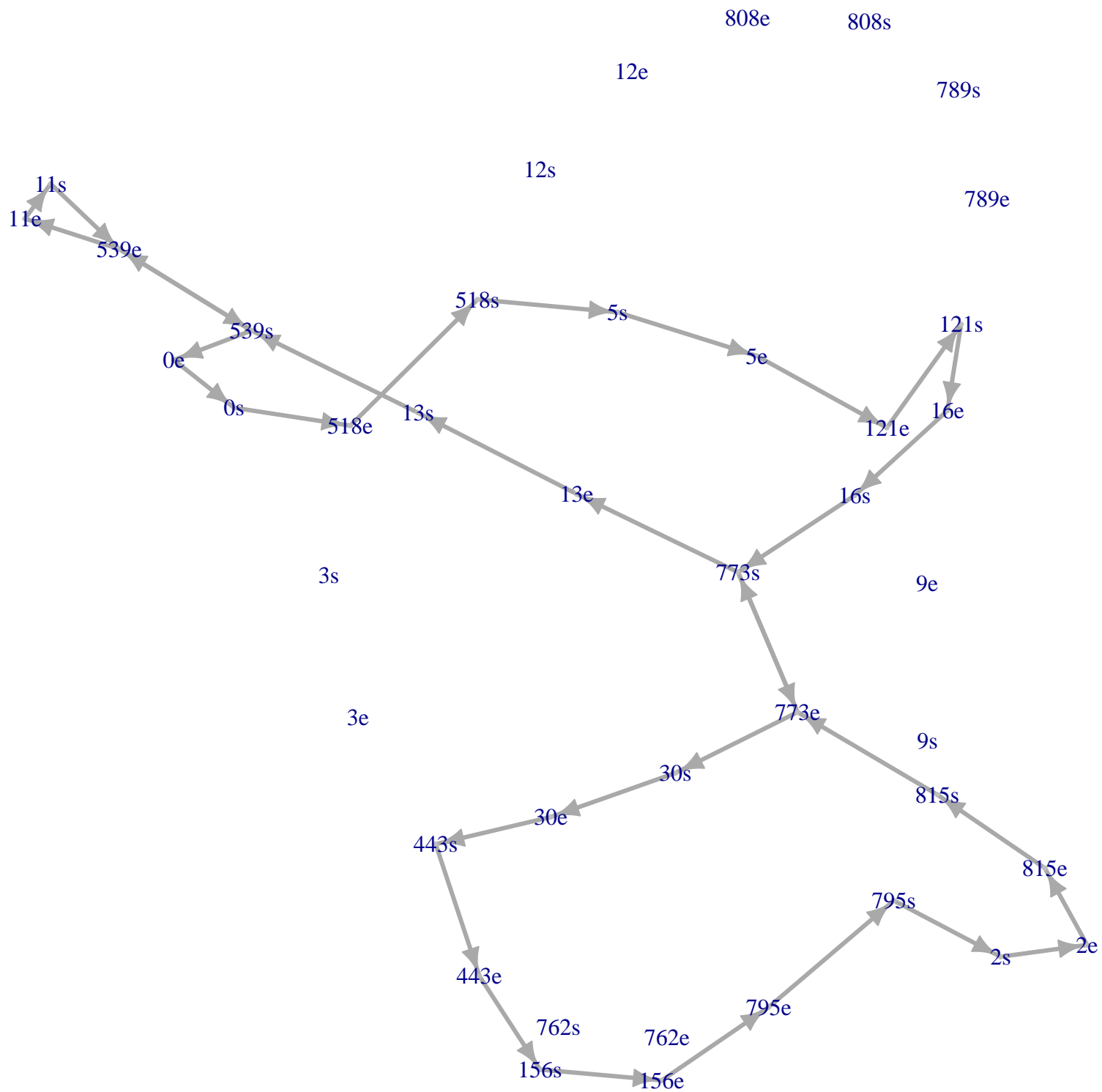

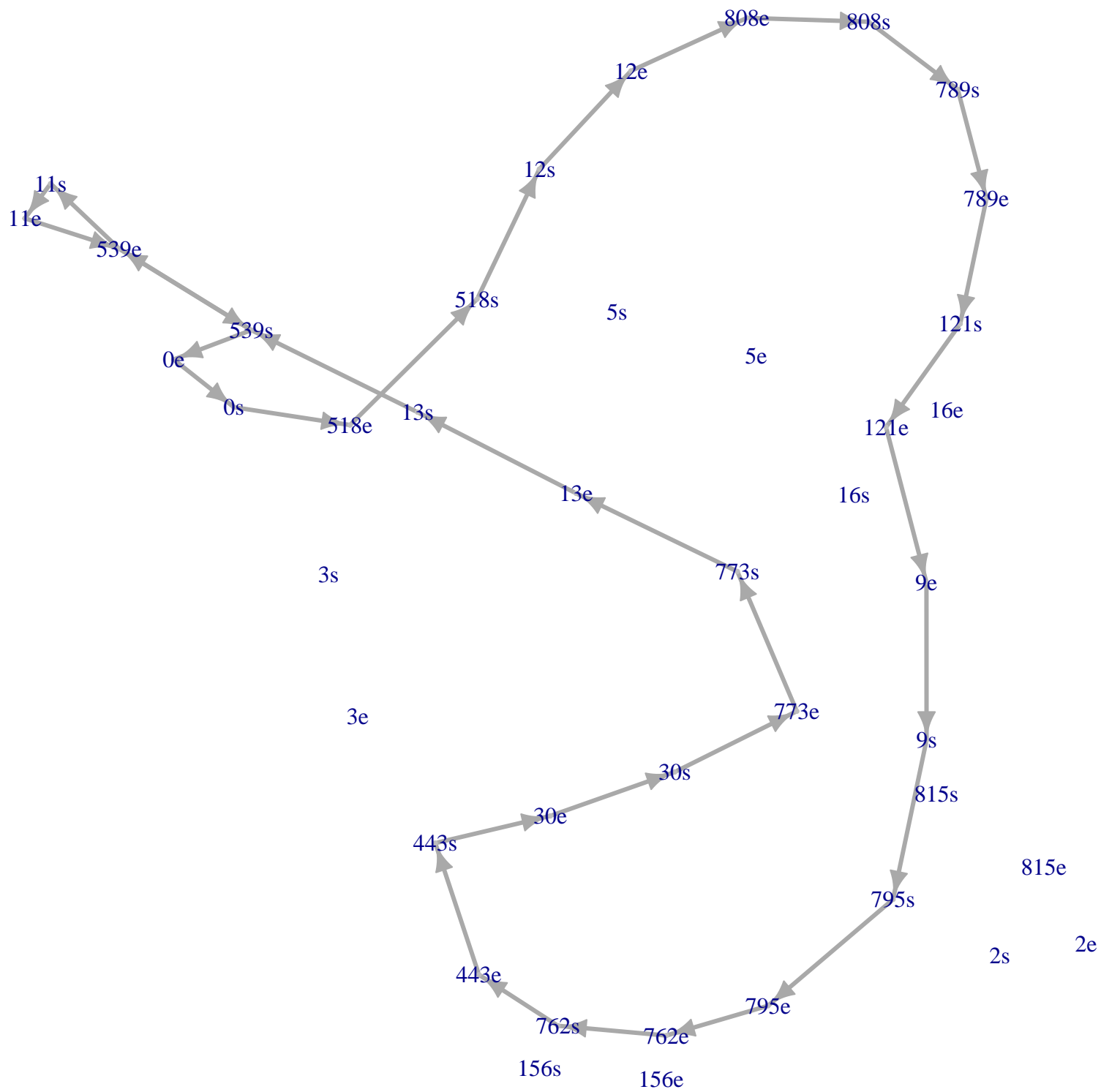

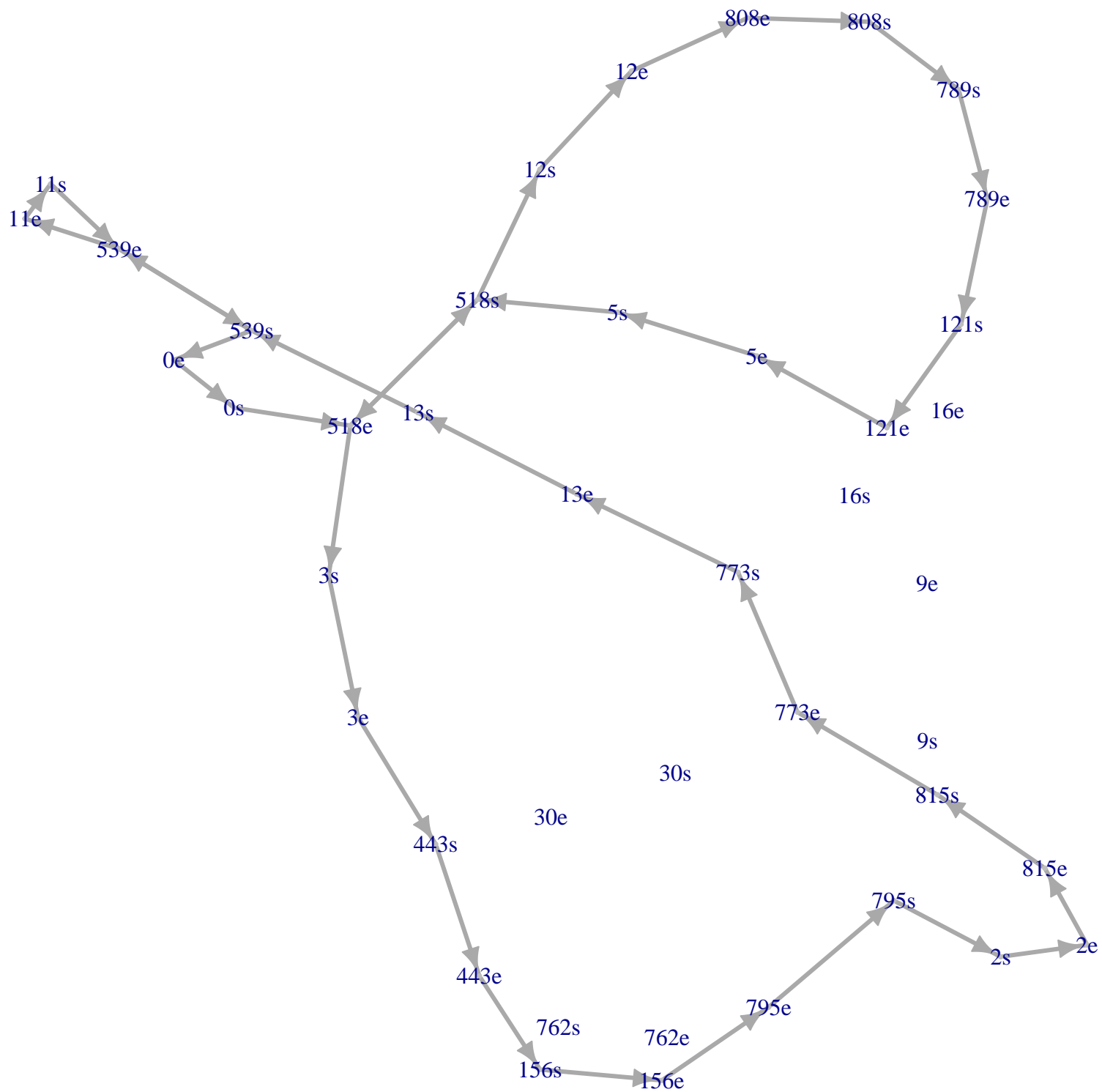

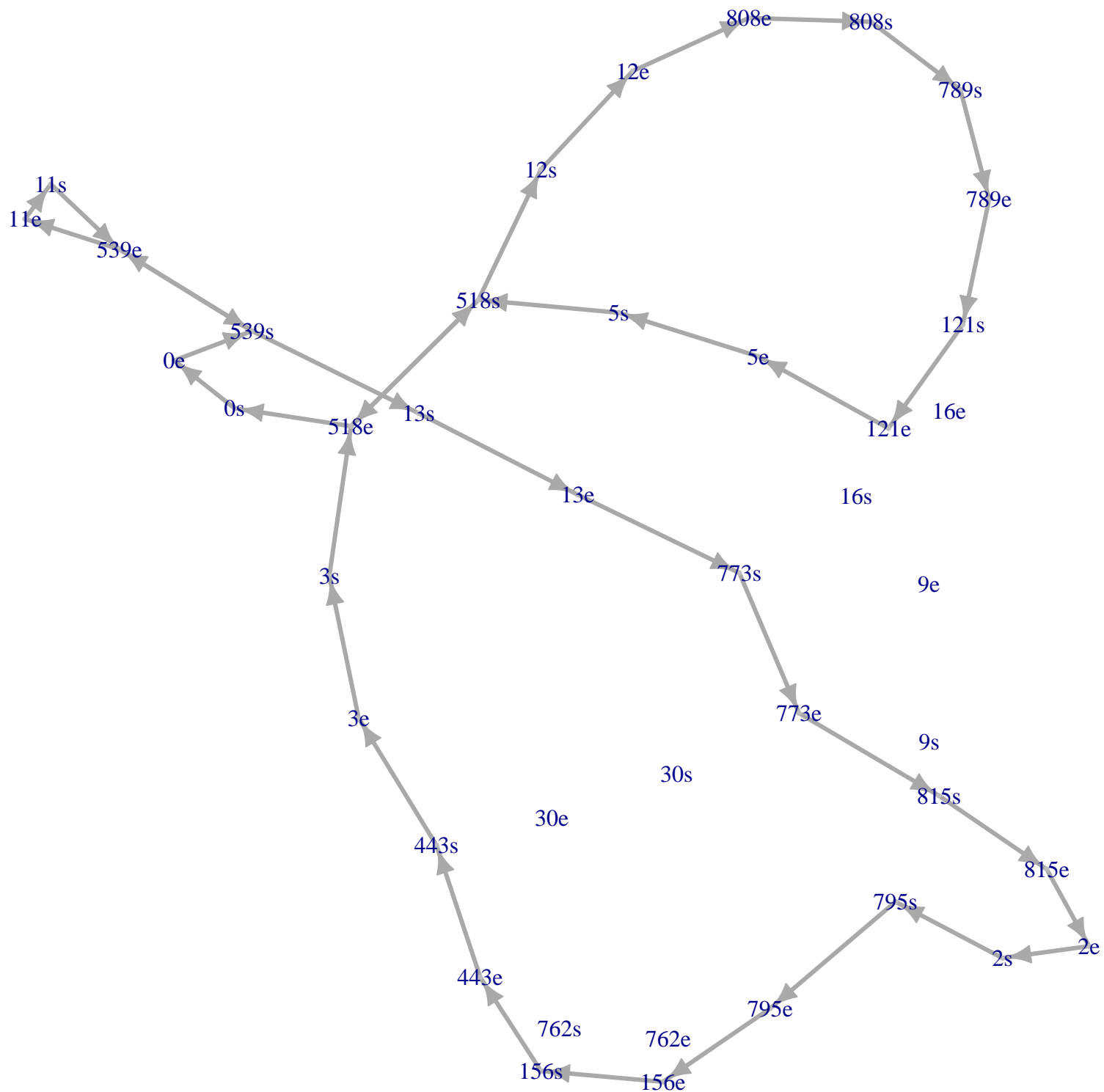

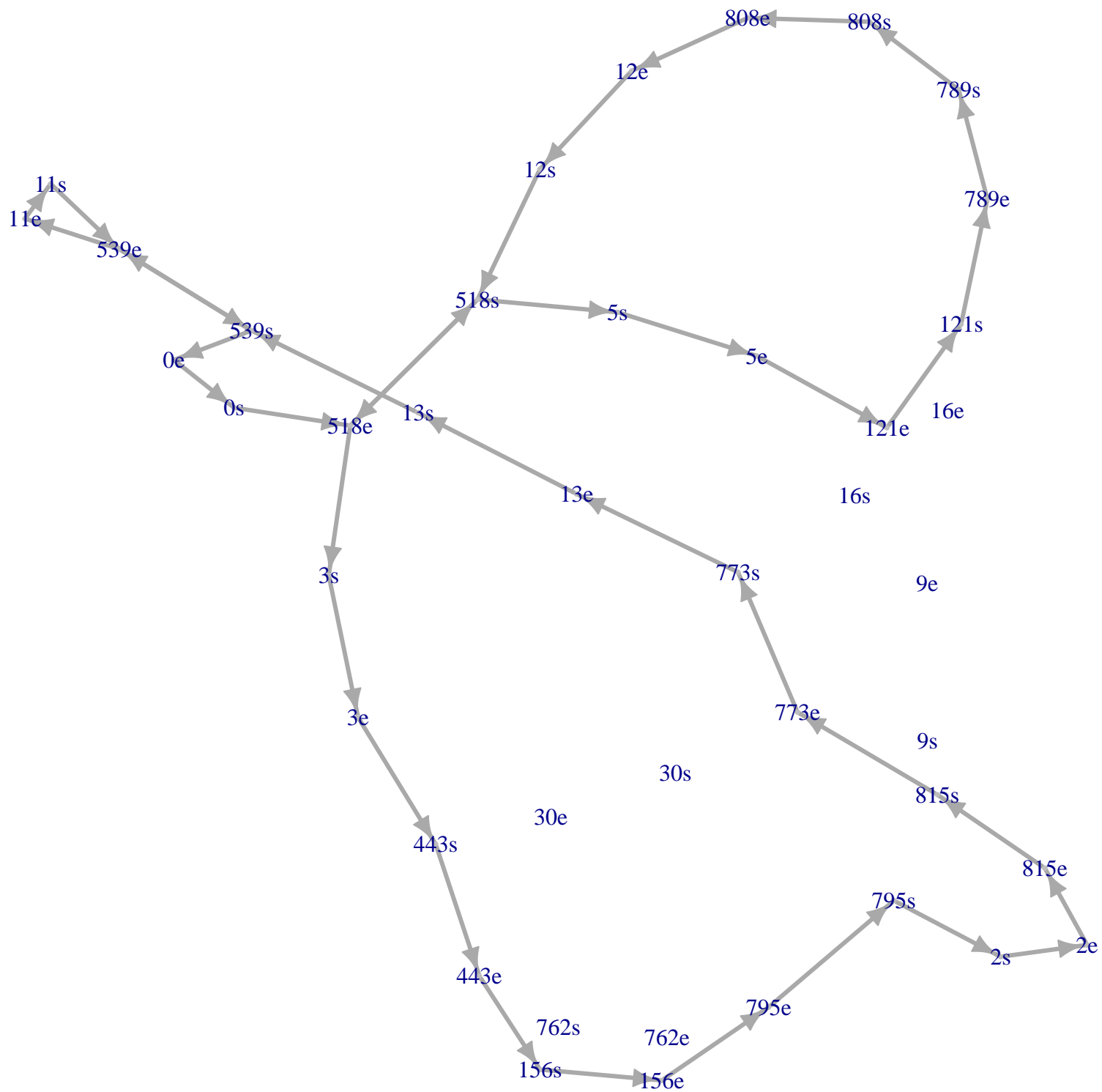

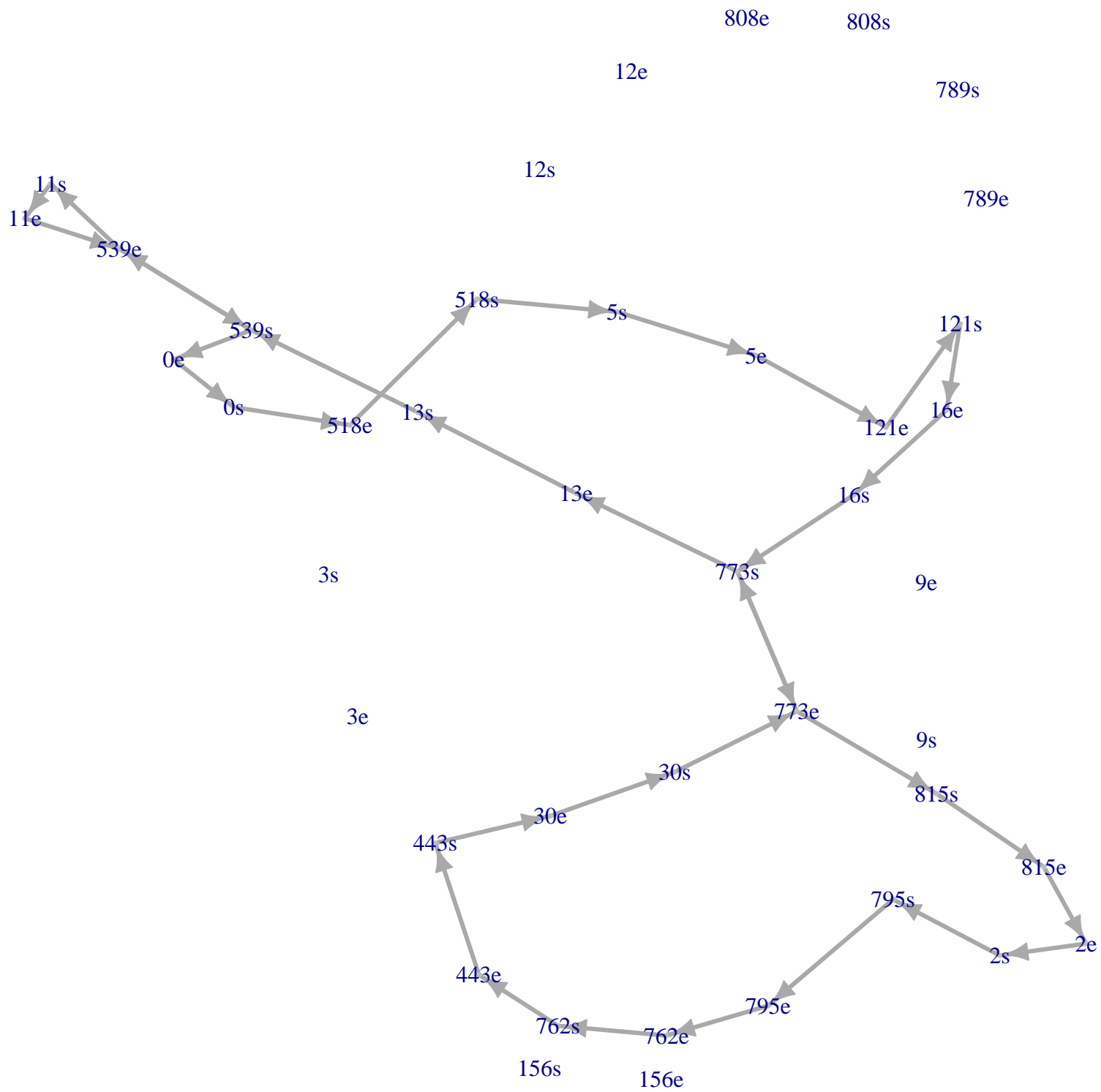

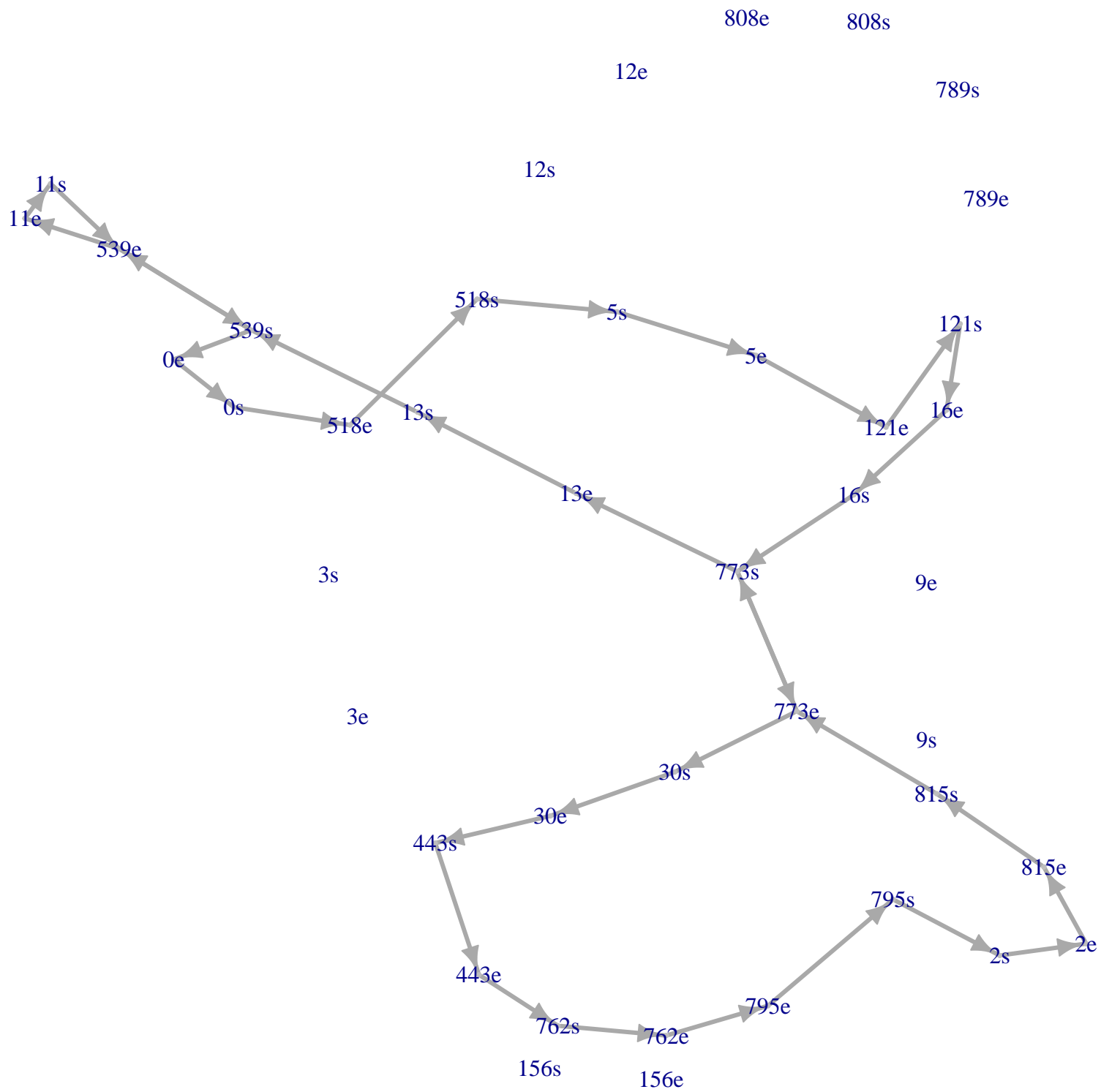

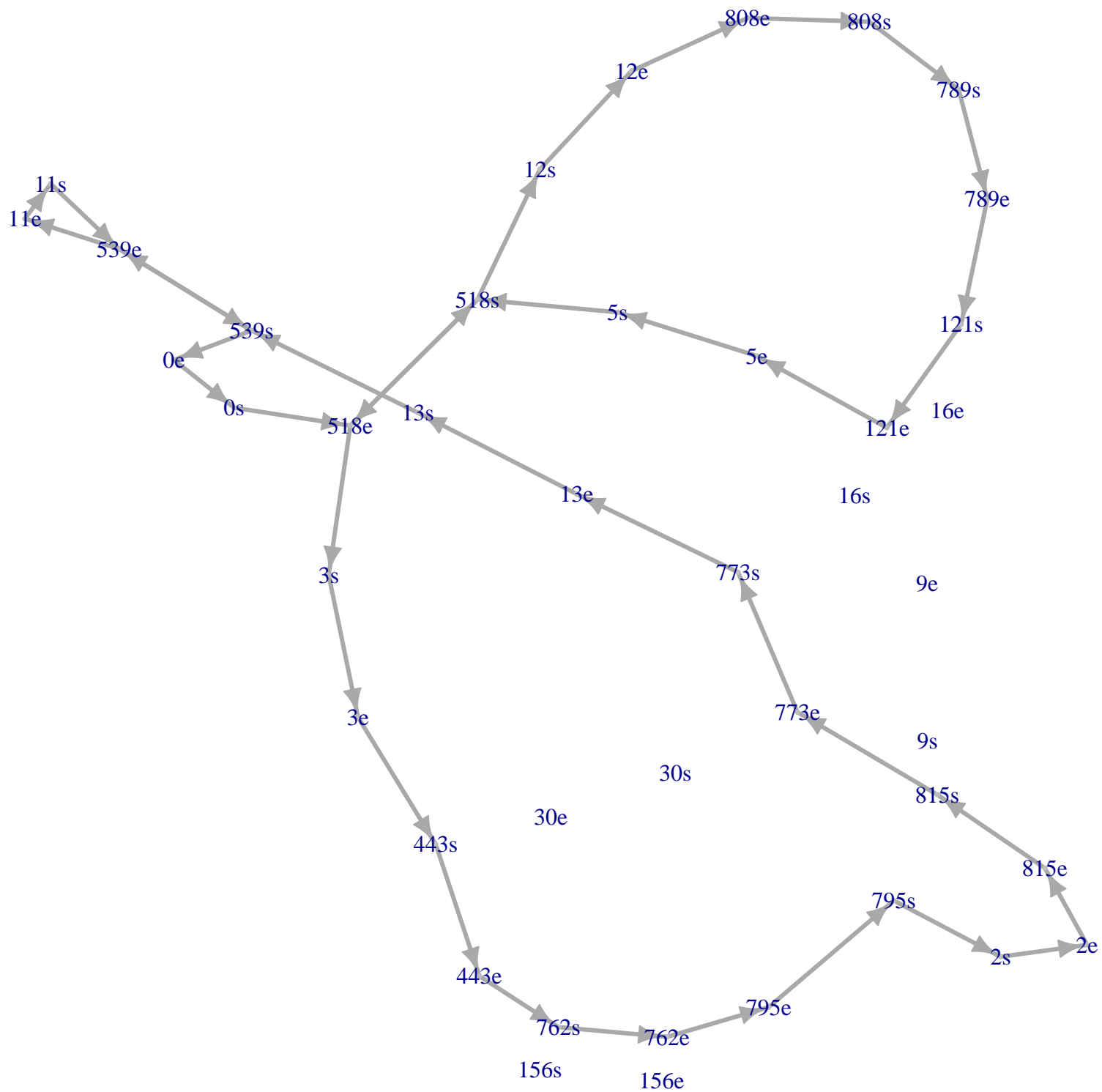

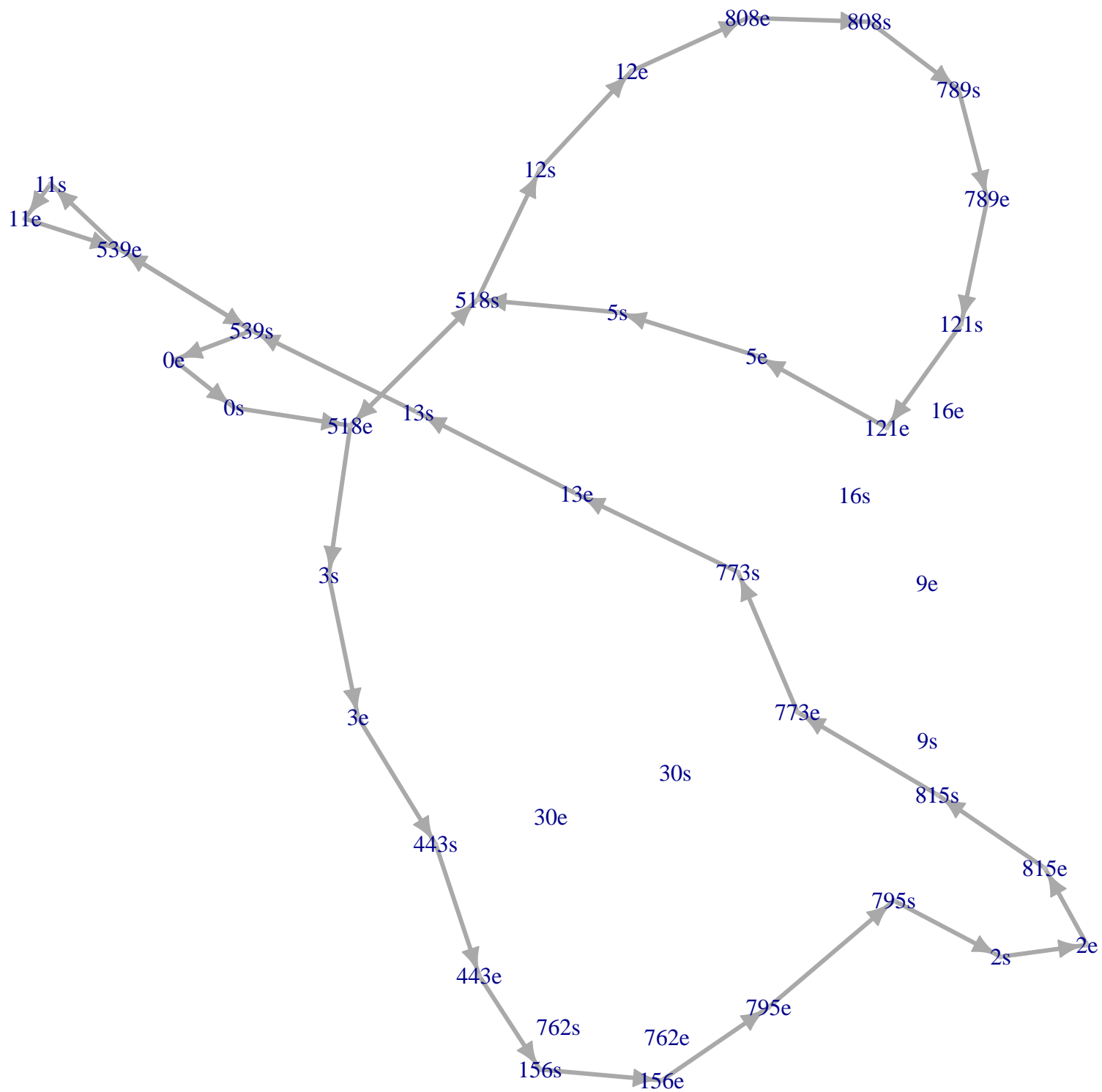

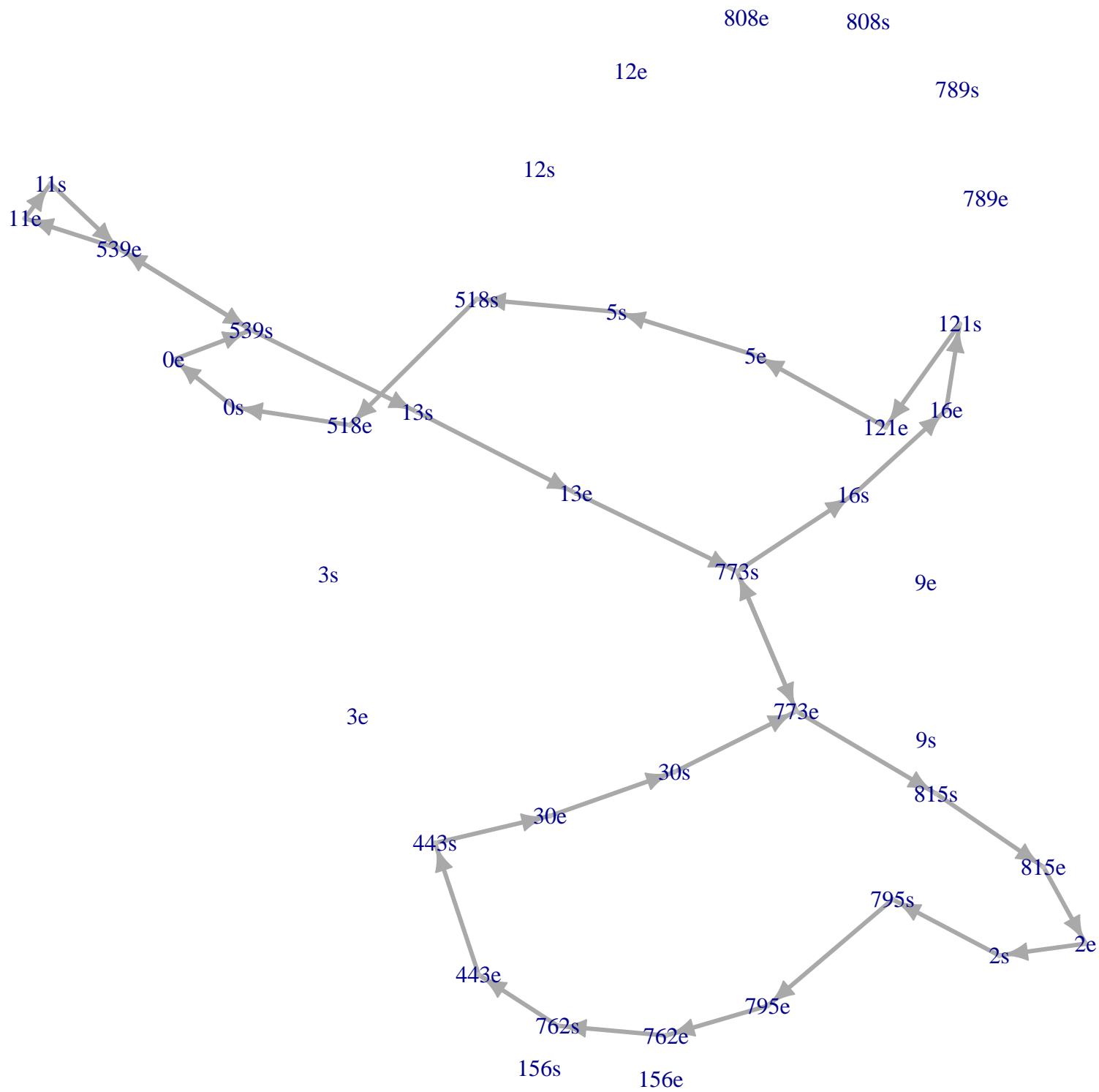

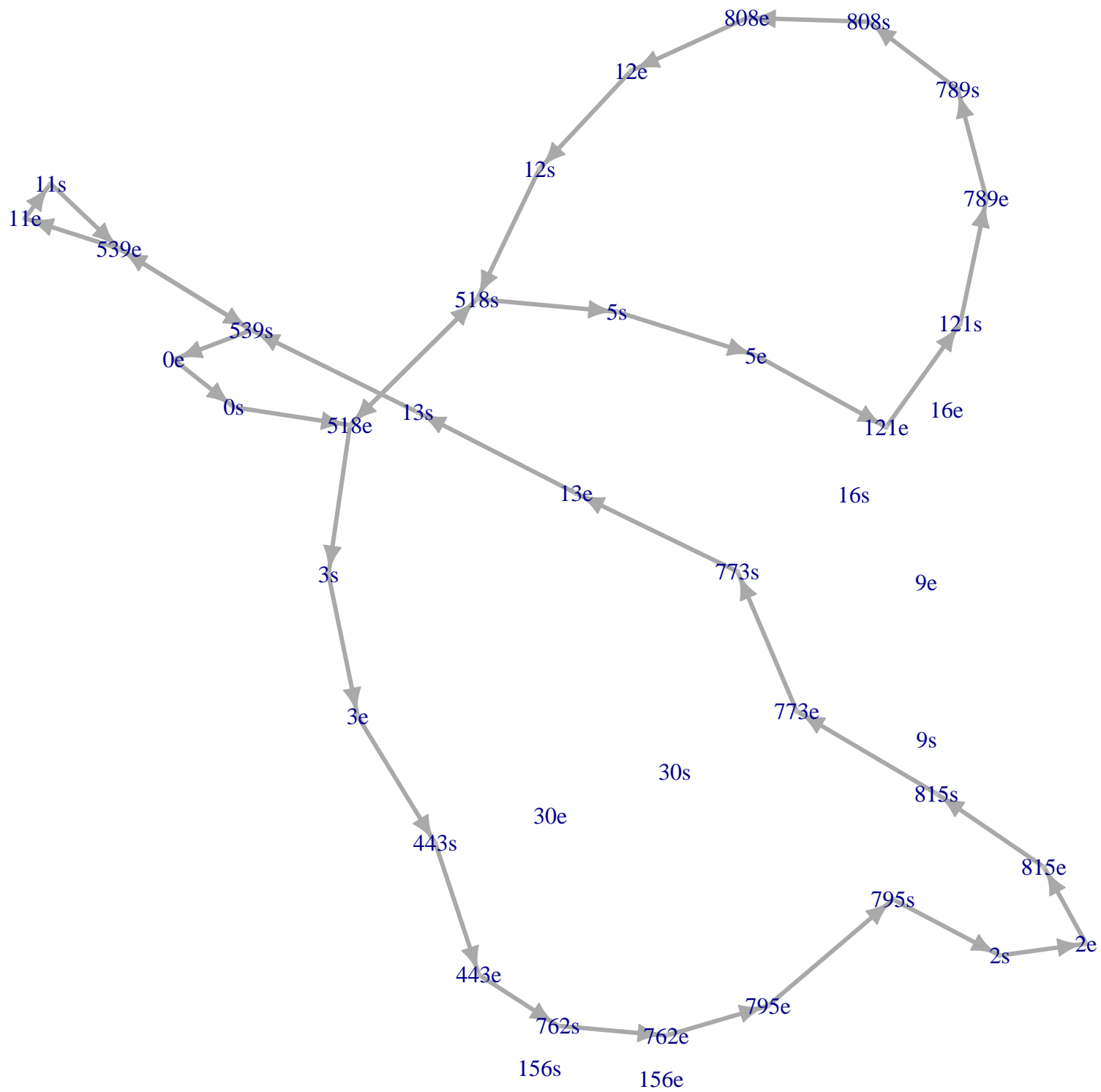

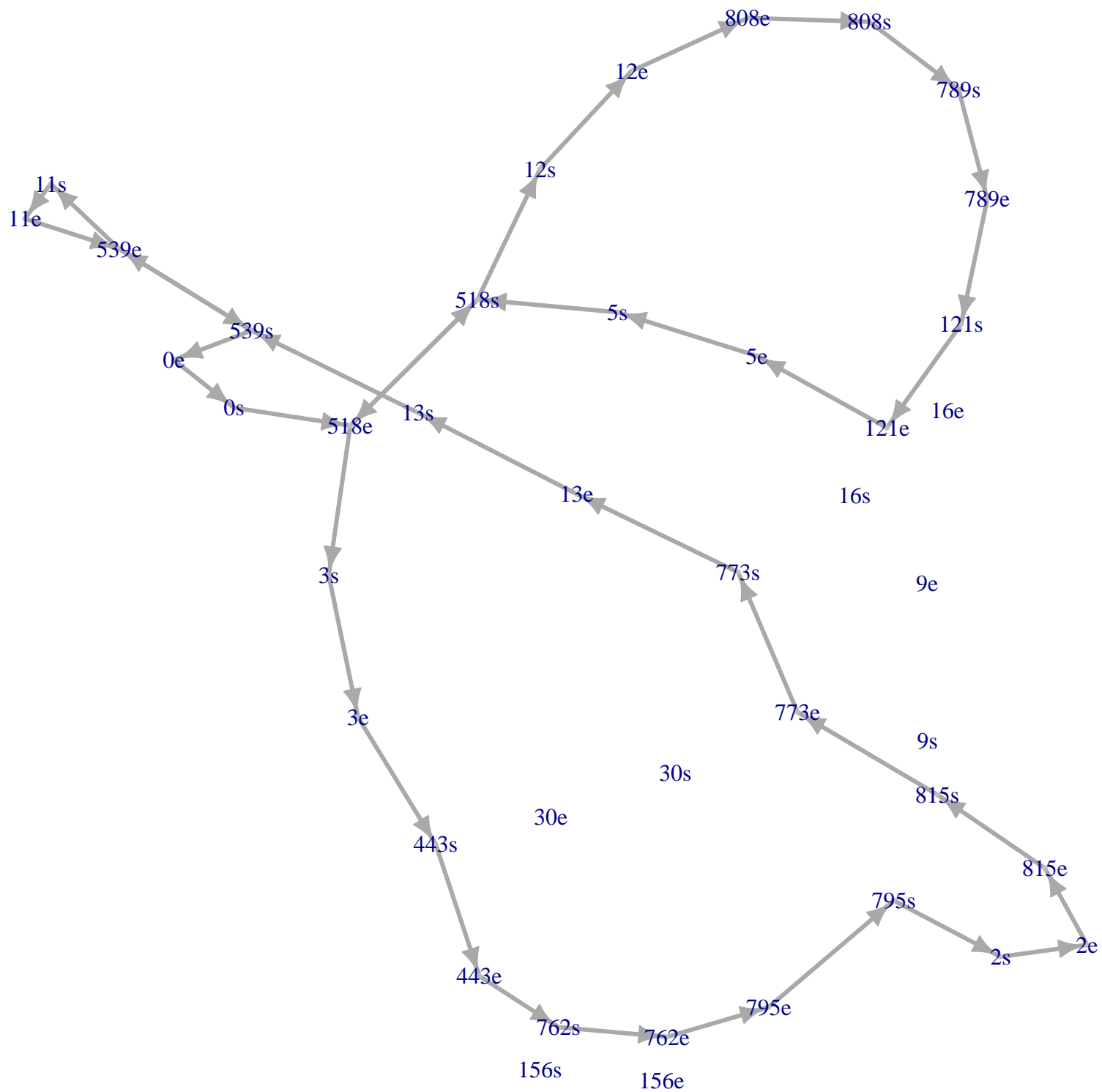

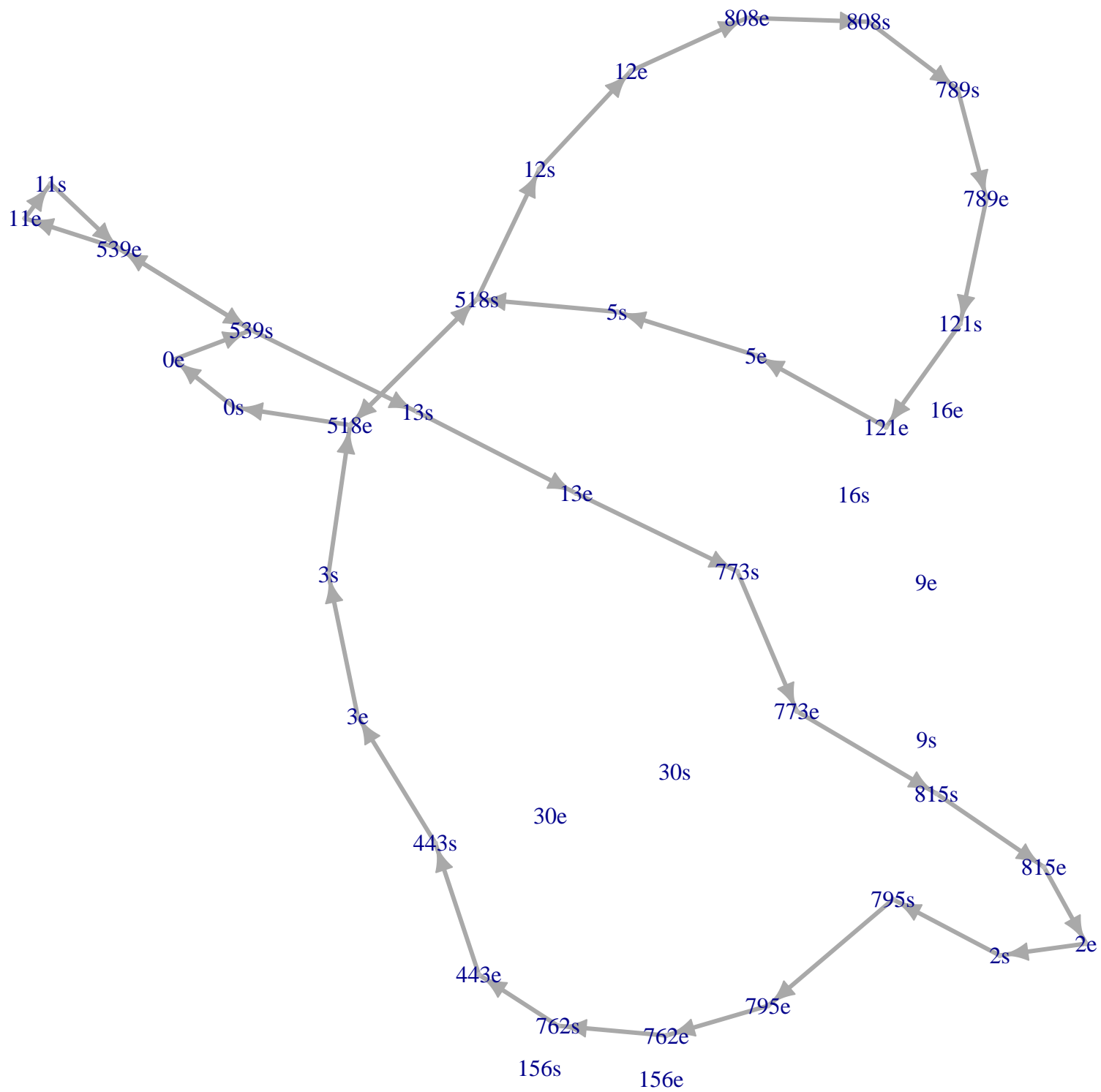

Supplement: lqac027_Supplemental_Files [file lqac027_supplemental_files.zip › Supplementary_Data_1.pdf]
